# Altered Levels of Histone Deacetylase OsHDT1 Affect Differential Gene Expression Patterns in Hybrid Rice

**DOI:** 10.1371/journal.pone.0021789

**Published:** 2011-07-08

**Authors:** Chen Li, Limin Huang, Caiguo Xu, Yu Zhao, Dao-Xiu Zhou

**Affiliations:** 1 National Key Laboratory of Crop Genetic Improvement, Huazhong Agricultural University, Wuhan, China; 2 Institut de Biologie des Plantes, UMR8618, Université Paris sud 11, CNRS, Orsay, France; University of Georgia, United States of America

## Abstract

Hybrids between different inbred varieties display novel patterns of gene expression resulted from parental variation in allelic nucleotide sequences. To study the function of chromatin regulators in hybrid gene expression, the histone deacetylase gene *OsHDT1* whose expression displayed a circadian rhythm was over-expressed or inactivated by RNAi in an elite rice parent. Increased *OsHDT1* expression did not affect plant growth in the parent but led to early flowering in the hybrid. Nonadditive up-regulation of key flowering time genes was found to be related to flowering time of the hybrid. Over-expression of *OsHDT1* repressed the nonadditive expression of the key flowering repressors in the hybrid (i.e. *OsGI* and *Hd1*) inducing early flowering. Analysis of histone acetylation suggested that *OsHDT1* over-expression might promote deacetylation on *OsGI* and *Hd1* chromatin during the peak expression phase. High throughput differential gene expression analysis revealed that altered OsHDT1 levels affected nonadditive expression of many genes in the hybrid. These data demonstrate that nonadditive gene expression was involved in flowering time control in the hybrid rice and that OsHDT1 level was important for nonadditive or differential expression of many genes including the flowering time genes, suggesting that OsHDT1 may be involved in epigenetic control of parental genome interaction for differential gene expression.

## Introduction

Epigenetic programming is suggested to be key mechanisms in the interaction between different genomes in hybrids [1]. Inbred parental genome interaction in hybrids leads to differential expression patterns that could be equal to the mid-parent (additivity), higher or lower than the mid-parent (nonadditivity), above the high-parent or below the low-parent (over- or underdominance) [2]. Novel patterns of gene expression or action in hybrids may result from parental variation in allelic nucleotide sequence and transcript abundance, which is supposed to be an important genetic component of phenotypic diversity [3], [4]. It is suggested that differential accumulation of allelic-specific transcripts in hybrids may contribute to heterosis [5].

Rice (*Oryza sativa*) is one of the most important crops in the world. Rice has become a model plant for plant biology with the availability of the complete genome sequences. Hybrids between two subspecies (i.e. *O. sativa ssp indica cv* and *O. sativa ssp japonica cv*) or different inbred varieties within a subspecies display high growth vigor which has substantially increased rice grain production during the last decades. Recent analysis by using high-throughput DNA sequencing technologies has revealed differential epigenetic modifications that correlate with changes in transcript levels between two rice subspecies and their reciprocal hybrids [6]. It is likely that multiple mechanisms including epigenetic processes are involved in parental genome interaction leading to distinct expression patterns in the hybrid, which are presently not understood.

Chromatin structure and remodelling are important components of genetic and epigenetic regulations of gene expression. Chromatin modification consists of covalent modifications of the N-terminal tails of the nucleosomal histones and DNA cytosine methylation [7]. Histone modifications including acetylation, methylation, phosphorylation, ubiquitinylation and others provide mechanisms to regulate gene expression through changes in chromatin states and by recruiting protein complexes that regulate transcription [8]. Histone lysine acetylation that is generally associated with gene activation is reversible, dynamic and regulated by histone acetyltransferases (HAT) and histone deacetylases (HDAC). Plant HAT and HDAC have been shown to play important roles in plant gene expression [9], [10]. Plant genome contains more than 20 genes encoding HDAC, which can be grouped into 3 classes [11]. Among them the HD2 class is found only in plants [12]. HD2 members have been shown to be involved in developmental and epigenetic pathways [13], [14], [15], [16], [17]. Specifically, an Arabidopsis HD2 protein, AtHDT1, is shown to be involved in nucleolar dominance in allopolyploid hybrids [13], [16]. In this work, we studied the function of a rice HD2 member, OsHDT1 (accession number: AK072845, LOC_Os05g51830), in regulating differential gene expression in hybrid rice. We show that the expression of *OsHDT1* displayed a circadian rhythm and that increased OsHDT1 could suppress overdominance expression of flowering time repressors in the hybrid leading to early flowering under long day condition, providing evidence of overdominance gene action in heterosis. In addition, alteration of OsHDT1 levels affected differential expression patterns of many other genes in the hybrid. These results indicate that OsHDT1 plays an important role in epigenetic processes regulating differential gene expression pattern in the hybrid.

## Results

### *OsHDT1* expression displays a circadian rhythm

**Figure 1:**
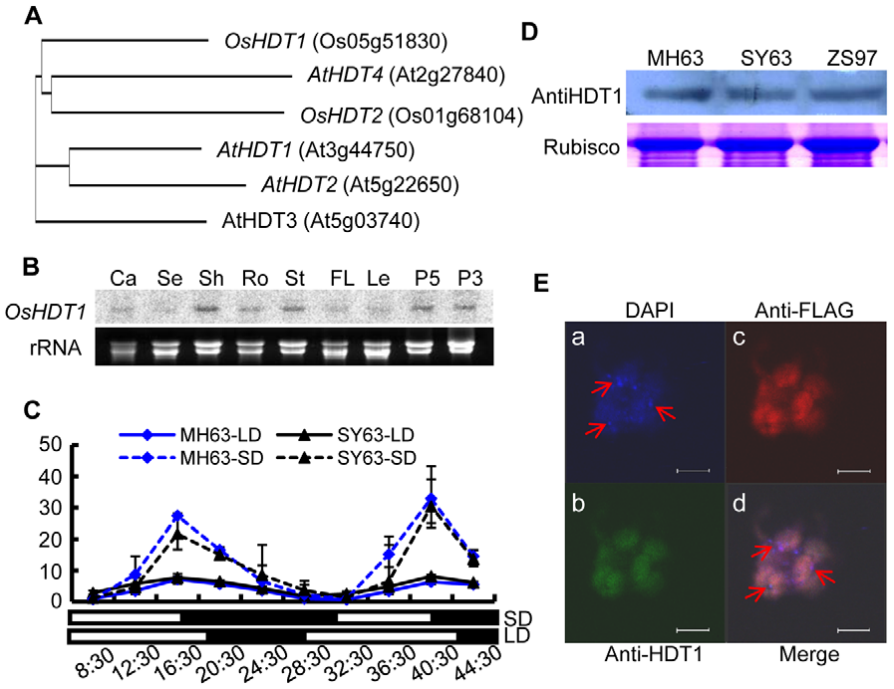
Expression profiles and subcellular localization of OsHDT1. (**A**). Phylogenetic relationship of HD2 subfamily proteins from *O. sativa* (*Os*), and *A. thaliana* (*At*). (**B**). *OsHDT1* transcripts detected by Northern blots in different tissues or developmental stages of MH63. Ca, callus; Se, seedling; Sh, shoot; Ro, root; St, stem; FL, flag leaf; Le, leaf; P5, panicle development stage 5; P3, panicle stage 3. (**C**). Diurnal expression of *OsHDT1* in MH63 and SY63 under long day and short day conditions revealed by qRT-PCR. Expression levels relative to MH63 at long day conditions 8:30 (set as 1) are presented. (**D**). OsHDT1 protein levels in MH63, ZS97 and SY63, detected by Western blots. The large subunit of Rubisco was revealed by gel staining as loading control. (**E**). Euchromatic localization of OsHDT1. Cells from transgenic rice expressing OsHDT1-Flag were detected by immunostaining using the anti-OsHDT1 and anti-Flag antibody simultaneously. **a**, stained with DAPI. **b**, examined by anti-OsHDT1 antibody. **c**, examined by anti-Flag antibody. **d**, merged image. Scale Bars = 2.94 µm. Arrows indicate positions of chromocenters.

Recent results have shown that differential epigenetic modifications correlated with changes in transcript levels among hybrids and parental lines [6]. Rice varieties (*O. sativa ssp indica cv*) Zhenshan 97 (ZS97) and Minghui 63 (MH63) are the parent lines of Shanyou 63 (SY63), one of the most widely cultivated hybrid rice in China. To study whether histone modification enzyme genes were involved in differential expression patterns in the hybrid, we chose to analyze the rice HD2 gene *OsHDT1* (Figure 1A). The expression of this gene was detected in different tissues/organs and developmental stages in MH63 (Figure 1B). Importantly, *OsHDT1* expression displayed a circadian rhythm under short day conditions (9 h light/15 h dark) (Figure 1C). Relatively lower expression levels were detected under long day (15 h light/9 h dark) conditions. There was no clear difference of *OsHDT1* expression between MH63 and SY63. The OsHDT1 protein was readily detectable in rice leaves by Western blots using antibodies raised against *E. coli*-produced OsHDT1 protein. There was no clear difference in OsHDT1 levels between the parent (MH63 and ZS97) and the hybrid (SY63) plants grown under same conditions (Figure 1D). The OsHDT1 protein was found to be distributed all over the nucleus as revealed by immunostaining with anti-Flag on cells isolated from transgenic rice expressing Flag-tagged OsHDT1 (Figure 1E, Figure S1). The same cells tested by anti-OsHDT1 displayed a similar localization pattern (Figure 1E). The immunostained areas did not overlap with the chromocenters revealed by 4′-6-Diamidino-2-phenylindole (DAPI), suggesting that OsHDT1 may be mostly localized in euchromatic regions.

### *OsHDT1* over-expression affects the flowering time of hybrid rice

The expression pattern of *OsHDT1* suggested that it might be involved in circadian regulation of gene expression. Circadian rhythms are shown to confer higher level of fitness in plants [18]. Importantly, it has been shown that altered circadian rhythms regulate growth vigor in *Arabidopsis* hybrids and allopolyploids [19]. To study whether *OsHDT1* played a role in hybrid gene expression, we produced *OsHDT1* over-expression and RNAi plants in the MH63 background (Figure S2A). Most of the transgenic plants had a single T-DNA insertion in the genome (Figure S2B). *OsHDT1* expression in the transgenic plants was tested by Northern blots for over-expression or by qRT-PCR for RNAi (Figure S2C). Over-expression lines (PU) 5, 8 and 9 and RNAi lines (PR) 1, 8 and 9 were selected for further analysis. The over-expression or RNAi of *OsHDT1* did not produce any visible morphological defects. No obvious change in overall histone acetylation was observed (Figure S3). However, examination of yield performance parameters revealed that the seed setting rate (total seed number/total floret number/plant) was reduced in the RNAi plants compared to wild type plants (Table 1). However, the seed setting rate of the RNAi negative plants was even lower, suggesting that the phenotype might be not related to the transgene. The over-expression of *OsHDT1* did not significantly affect the different parameters of the yield performance including panicle number, panicle length, seed setting rate, one-thousand-grain weight etc. (Table 1). Examination of the two next generations confirmed the above observation.

**Table 1:** Performance of wild type and *OsHDT1* transgenic plants under natural long-day conditions.

Genotype	Days to heading	Panicle length	Grain number per panicle	Seed setting rate	1000-grain weight
PU	95.40±2.51	24.39±0.38	79.30±9.33	78.58±1.51	27.37±1.30
PU-	94.80±1.32	24.33±0.35	80.61±1.55	75.62±1.88	27.89±0.42
MH63	96.20±2.2	25.62±0.66	98.71±0.04	82.05±0.87	28.89±0.76
PR-	95.20±1.73	24.29±0.53	68.55±0.41	62.14±2.29	27.36±0.33
PR	93.67±0.75	25.05±0.54	77.78±8.17	69.99±5.57^*^	26.3±0.76
FU	78.87±0.40^*^_#_	27.1±0.56	111.60±4.72	76.56±1.33	28.69±0.28
FU-	90.73±0.31	26.69±0.51	112.28±9.26	74.84±1.09	28.28±0.40
SY63	88.20±1.67	26.31±0.72	118.07±3.81	78.37±3.41	28.12±0.37
FR-	88.47±1.11	25.27±0.27	100.88±0.20	70.58±4.68	28.06±0.93
FR	88.03±0.78	26.51±0.05	100.14±1.87	68.15±3.55^*^	27.53±0.21

Data presented were from a randomized complete block design with three replicas. Every replica included the three lines per transgene (positive or negative) tested in Figure 3A. Every line contained 30 plants. Asterisks indicate ranking by Least Significant Difference (LSD) tests at highly significant (*P<0.01) differences relative to the corresponding wild type plants. Wells indicate highly significant (#P<0.01) differences relative to the corresponding transgene-negative plants.

**Figure 2:**
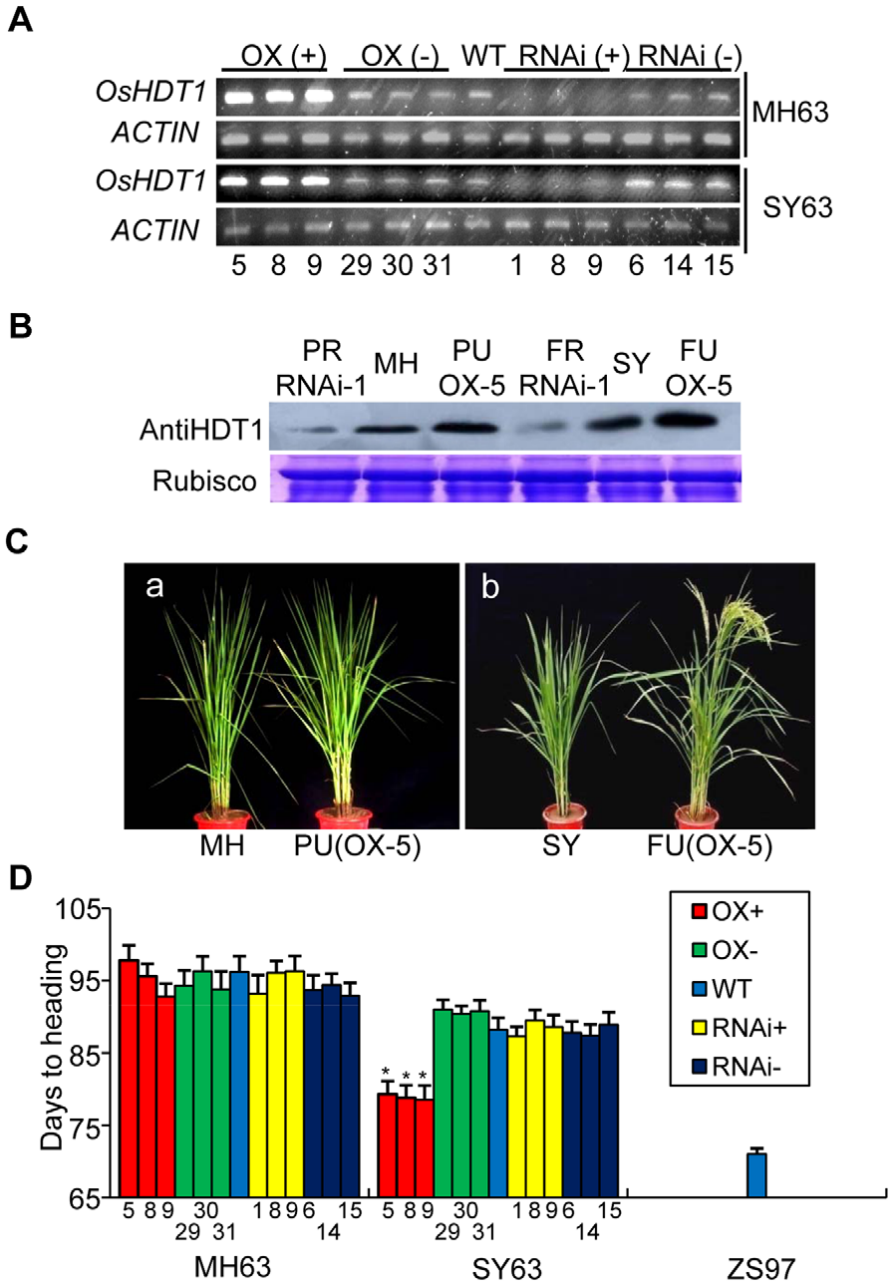
*OsHDT1* over-expression induces early flowering in the hybrid. (**A**). Comparison of *OsHDT1* transcript accumulation in transgenic and wild type (WT) parent MH63 and hybrid SY63 by semi-quantitative (22 cycles) RT-PCR. Three independent over-expression (OX) positive (+, lines 5, 8, and 9) or negative (−, lines 29, 30 and 31) and RNAi positive (+, line 1, 8 and 9) and negative (−, lines 6, 14 and 15) transgenic lines in both MH63 and SY63 backgrounds were analyzed. Rice actin transcripts were detected as controls. (**B**). OsHDT1 protein levels in RNAi (line 1), over-expression (OX, line 5) and wild type MH63 (MH) and SY63 (SY) detected by Western blots using anti-OsHDT1. RNAi plants in MH63 and SY63 were designated as PR and FR, respectively; Over-expression plants in MH63 and SY63 were designated as PU and FU, respectively. (**C**). Over-expression of *OsHDT1* induces an early heading flowering phenotype in SY63 background. **a**, comparison between MH63 and PU (line 5) at 98 days after sowing. **b**, comparison between SY63 and FU (line 5) taken at 88 days after sowing. (**D**). Heading dates of transgenic positive and negative lines in MH63 and SY63 backgrounds in comparison with the respective wild type lines under natural long day conditions. The transgenic lines used are indicated at the bottom. Asterisks indicate ranking by LSD test at highly significant (*P<0.01) differences relative to the corresponding wild type plants.

To test if the altered expression of *OsHDT1* in MH63 could affect hybrid growth, three independent T3 transgenic plants (PU or PR) were used to pollinate ZS97 (as practiced for SY63 seed production in agriculture). Transgene-negative plants (PU- or PR-) were used in the crosses as controls. The respective transgenic-positive hybrids were named FU or FR, the transgenic-negative hybrids as FU- or FR-. RT-PCR analysis of 3 independent transgenic lines (both parent and hybrid) in comparison with the wild type and the negative controls confirmed the down-regulation of *OsHDT1* in PR and FR and the up-regulation of the gene in PU and FU (Figure 2A). Western blot analysis using anti-OsHDT1 detected a decrease of OsHDT1 protein level in the RNAi parent (PR) and hybrid (FR) plants and an increase in the over-expression parent (PU) and hybrid (FU) plants compared to the wild type parent or hybrid (Figure 2B). Growth and yield traits were surveyed for the hybrids. The FU and FR hybrids did not exhibit any visible growth difference from SY63, except that the heading date (flowering time) of the over-expression hybrid (FU) lines was significantly earlier than SY63 and the transgene-negative hybrid controls (FU-) under natural long day conditions (>14 h) (Figure 2C, 2D; Table 1). These data together suggested that increased OsHDT1 level may alter flowering time-related gene expression in the hybrid, while without a clear effect in the parent background.

### *OsHDT1* over-expression suppresses overdominance expression of flowering time genes in hybrid rice

Rice is a short day plant. *Hd1* (*Heading date 1*), the rice orthologue of Arabidopsis *CONSTANS* (*CO*), activates *Hd3a* (*Heading date 3a*, the orthologue of Arabidopsis florigen gene *FLOWERING LOCUS T*, *FT*) under short day conditions but repressed it under long day conditions [20], [21]. Identification of natural variation affecting flowering time has revealed genes such as *Ehd1* (*Early heading date1*) and *Ghd7* (*Grain number*, *plant height and heading date 7*), which encode unique transcription regulators in rice [22], [23]. *Ehd1*, a B-type response regulator, up-regulates *Hd3a* expression and mainly confer short day-dependent flowering promotion in rice [22] . *Ghd7*, a CCT-domain protein, is expressed under long day conditions. It represses *Ehd1* expression and mainly confers long day-dependent flowering repression [23].

**Figure 3:**
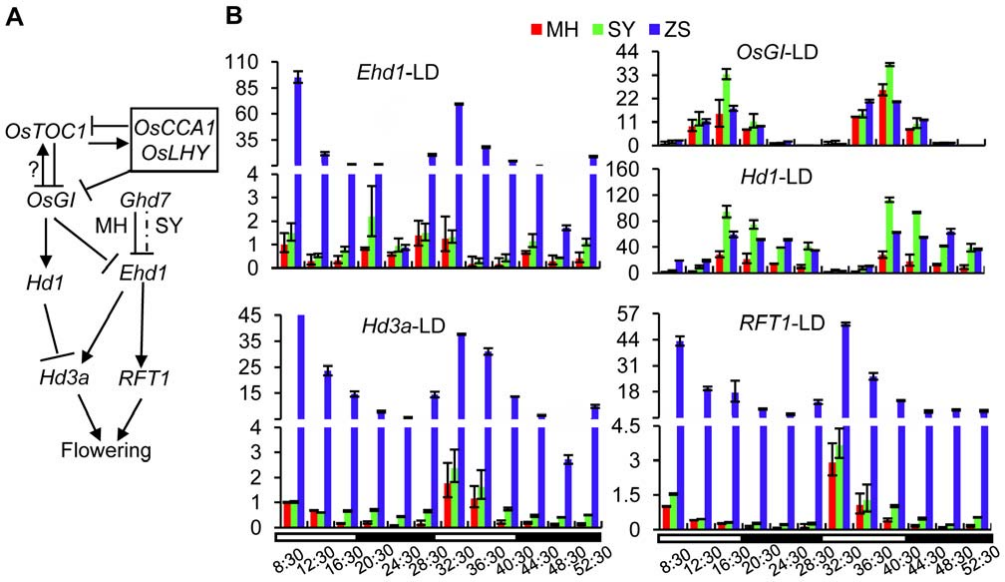
Diurnal expression of rice key flowering time genes under long day conditions. (**A**). Model for heading date genetic control pathways in MH63 and SY63 under long day conditions. Bars, repression; arrows, activation. Dashed lines indicate reduced activity in SY63. (**B**). Diurnal expression patterns of *Ehd1*, *OsGI*, *Hd1*, *Hd3a* and *RFT1* in SY63, MH63 and ZS97 under long day condition. In all panels, the mean values of each point are based on the averages of three biological repeats calculated using the relative quantification method. Values relative to MH63 at 8:30 (set arbitrarily as 1) are presented. Light and dark periods are indicated by white and black, respectively. Time points of the subjective day for sample harvesting are indicated. Error bars, s.e.m. from 3 biological repeats.

**Figure 4:**
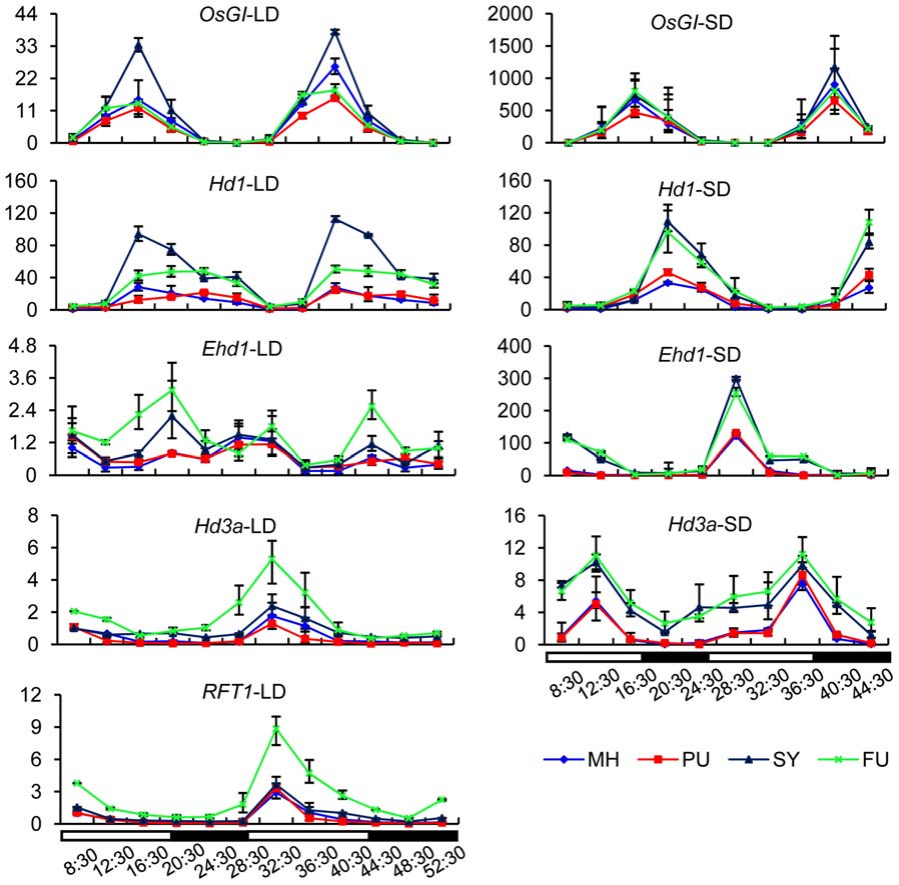
Effects of *OsHDT1* on flowering time gene expression. Diurnal expression of *OsGI*, *Hd1*, *Ehd1*, *Hd3a* and *RFT1* in MH63, *OsHDT1* over-expression in MH63 (PU), SY63 and *OsHDT1* over-expression in SY63 (FU) under long day (left) and short day (right) conditions, revealed by qRT-PCR. Values relative to the transcript levels in MH63 at 8:30 (arbitrarily set as 1) are presented. Error bars, s.e.m. from 3 biological repeats.

Under short day conditions, the expression of *Hd3a* is up-regulated by *Hd1* and *Ehd1*. Under long day conditions, the expression of *Ehd1* is repressed by *Ghd7*, while *Hd1* becomes as a repressor of *Hd3a* (Figure 3A). *RFT1* (*RICE FLOWERING LOCUS T1*) is the florigen gene that can be activated by *Ehd1* [24]. The hybrid parents differ significantly in heading date under natural long day conditions. MH63 flowered at the age of 96 days, while ZS97 flowered at 71 days (Figure 2D). This difference is likely to be mainly due to the repression of the flowering activator *Ehd1* by *Ghd7* in MH63, which is defective in ZS97 [23]. qRT-PCR analysis of RNA isolated from 35-day old leaves revealing higher expression of *Hd3a*, *RFT1* and *Ehd1* in ZS97 than in MH63 and SY63 under long day conditions confirmed this hypothesis (Figure 3B). *Hd1* shows a comparable expression level in MH63 and ZS97 under either long day or short day conditions [23]. However, the rhythmic expression of *Hd1* and *OsGI* (*OsGIGANTEA*, an upstream activator of *Hd1*) was higher in SY63 than the parents under same conditions (Figure 3B), suggesting a nonadditive effect (i.e. overdominance) on the expression of these genes in the hybrid. In contrast, the expression levels of *Hd3a*, *RFT1* and *Ehd1* in SY63 were close to that of MH63 (Figure 3B), which was correlated with the relatively late flowering observed in SY63 compared to ZS97 (Figure 2D). The reduced expression of *Hd3a*, *RFT1* and *Ehd1* may result from a collective action of both the increased expression of *Hd1* and the presence of active *Ghd7* (MH63 allele) in the hybrid. The increased expression of *Hd1* and *OsGI* in SY63 under long day conditions was reduced by *OsHDT1* over-expression (FU), while there was no clear difference for the two genes between the transgenic (PU) and wild type (MH63) parents under long day conditions (Figure 4). This suggested that increased *OsHDT1* expression reduced the overdominance expression of *Hd1* and *OsGI* in the hybrid. In contrast, higher expression of *Hd3a*, *RFT1* and *Ehd1* was observed in the over-expression hybrid (FU) compared to SY63. The higher level of *Hd3a* may be, at least in part, a consequence of the repression of *OsGI* and *Hd1* by *OsHDT1* in the hybrid. The increased expression of *Ehd1* might be essentially due to the repressed expression of *OsGI*, as the expression of *Ghd7* that represses *Ehd1* under long day conditions was not altered by the *OsHDT1* over-expression (Figure S4). In addition, recent results indicate that the mutation of *OsGI* increases *Ehd1* expression under long day condition [25]. Increased *Ehd1* in turn induced *RFT1* that has been shown to be the florigen gene in long day conditions [24], explaining the early flowering phenotype of the over-expression hybrid. Under short day conditions, the expression of *Hd3a*, *Ehd1* and *Hd1* was higher in the hybrid than in MH63 (Figure 4). There was no clear difference for these genes between the transgenic and wild type parents or hybrids (Figure 4). The expression of *OsCCA1*, *OsLHY* and *OsTOC1*, which are putative upstream regulatory genes of *OsGI* was higher in the hybrid than the parent, but there was no clear difference between SY63 and FU or between MH63 and PU (Figure S4). These data suggested that increased *OsHDT1* expression suppressed the overdominance expression of *OsGI* and *Hd1* in the hybrid under long day conditions, which led to early flowering.

### Effect of OsHDT1 on histone acetylation

**Figure 5:**
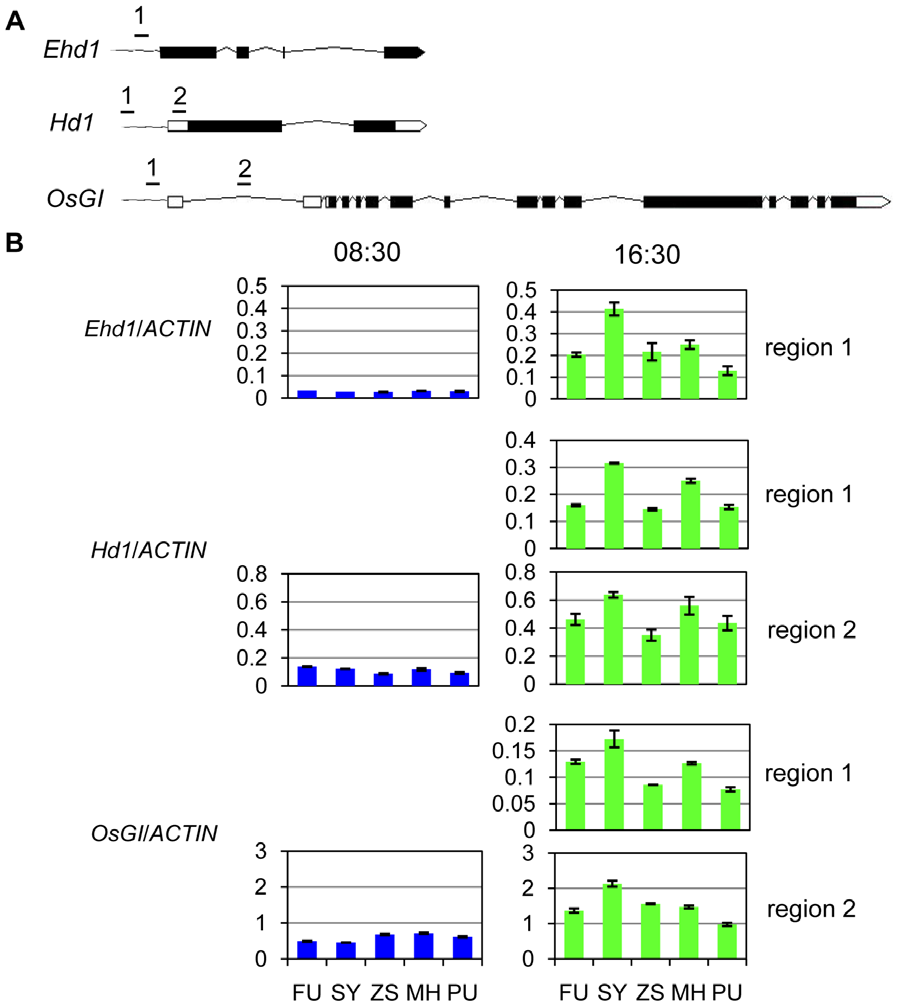
Histone H4 acetylation on flowering time genes. Chromatin isolated from 35 day-old rice leaves of SY63 (SY), MH63 (MH), ZS97 (ZS) and *OsHDT1* over-expression in SY63 (FU) and MH63 (PU) background, harvested at 8:30 and at 16:30 (of the subjective day) under long day conditions were precipitated by antibody of acetylated histone H4. The precipitated DNA was analyzed by real-time PCR using the primer sets corresponding to the 5′ region of the genes (A). The 5′ region of rice actin gene was amplified as internal control. One region for each gene was analyzed at 8:30 and two regions of Hd1 and OsGI were analyzed at 16:30. Values relative to actin gene are shown (B). Error bars, s.e.m. from 3 biological repeats.

To test whether *OsHDT1* over-expression altered histone acetylation on flowering time genes, chromatin fragments isolated from MH63, ZS97, SY63, PU and FU were precipitated by antibodies against acetylated H4. Because the expression of *OsGI* and *Hd1* is at the lowest level at 8:30 and highest at 16:30, samples were harvested at the two time points. Two regions of the 5′-end of *Hd1* and *OsGI* and one region of *Ehd1* were analyzed by qPCR (Figure 5A). The 5′ region of rice actin gene was tested as reference for normalization. At 16:30, histone H4 acetylation on the 3 genes was found to be lower in PU compared to MH63 and in FU compared to SY63 (Figure 5B), suggesting that *OsHDT1* over-expression had a negative impact on histone H4 acetylation on these genes in both parent and hybrid backgrounds. Region 2 of both *OsGI* and *Hd1* displayed higher acetylation than region 1 (Figure 5B). Only Region 2 of the two genes and region 1 of *Ehd1* were tested at 8:30. At this time point acetylation on the three genes was about 3–5 folds lower compared to at 16:30. There was no clear difference observed between the different genotypes at this time point. These observations indicated that acetylation was likely to correlate with the rhythmic expression of *Hd1* and *OsGI* and that *OsHDT1* over-expression had an effect at the time when acetylation was high. The deacetylation promoted by *OsHDT1* over-expression correlated with the repression of *Hd1* and *OsGI* in the hybrid. Although acetylation on *Ehd1* was also reduced, the expression of the gene was low at this time point. The higher expression of the gene in FU than in SY63 might be mainly due to the repression of *OsGI* by elevated OsHDT1 (Figure 3, Figure 4). In addition, SY63 displayed higher H4 acetylation compared to the parents, which was correlated with the increased expression of *Hd1* and *OsGI* in the hybrid (Figure 3).

### Impact of altered OsHDT1 levels on gene expression in rice hybrid

**Figure 6:**
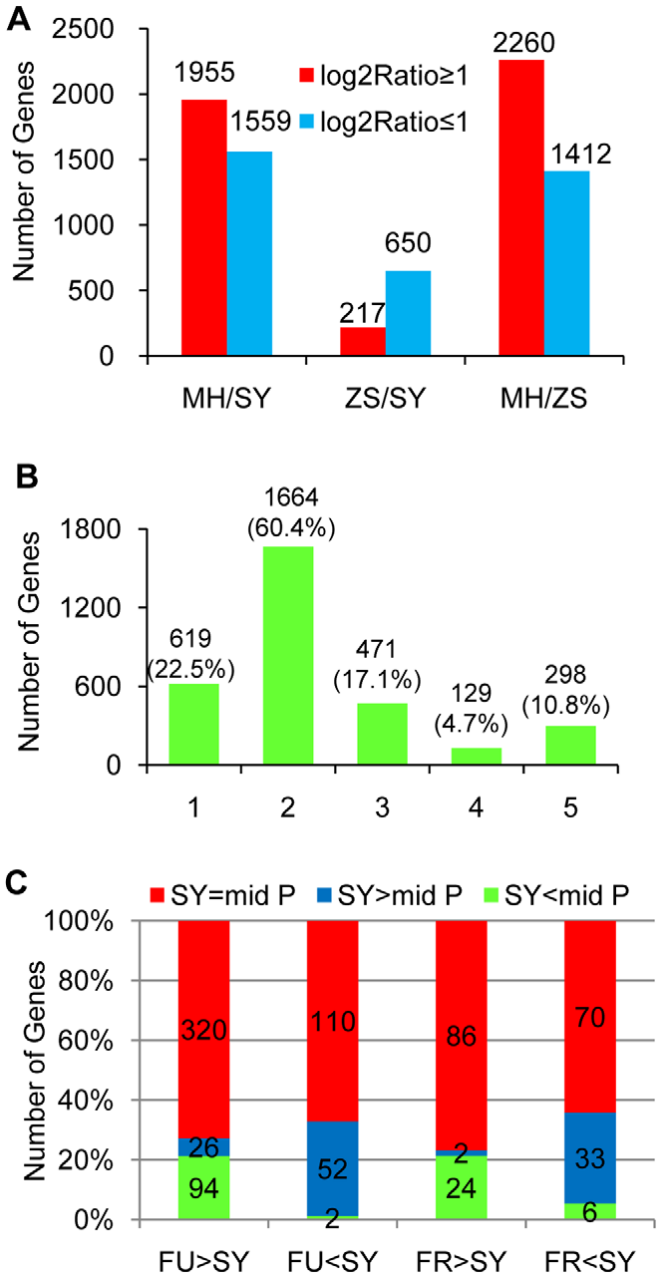
Differential gene expression patterns in hybrid SY63. (**A**). Numbers of differentially expressed genes (FDR≤0.001 and |log_2_Ratio|≥1) in MH63 versus SY63, ZS97 versus SY63 and MH63 versus ZS97. Black bars: up-regulated genes; grey bars, down-regulated genes. (**B**). Numbers of differentially expressed gene in SY63 compared to mid-parent (|log_2_Ratio|≥1). 1, SY<mid-parent; 2, SY = mid-parent; 3, SY>mid-parent; 4, SY<low-parent; 5, SY>high-parent. (**C**). Percentage of nonadditive and additive gene in SY63 affected by *OsHDT1* over-expression (FU) and RNAi (FR).

To study whether altered *OsHDT1* levels affected the expression of other genes in the hybrid, we compared genome-wide transcript abundance between MH63, ZS97, SY63, FR and FU. By using the high throughput digital gene expression analysis that sequence restriction enzyme cut tags and is extremely sensitive for detecting differential gene expression between samples [26], [27]. RNA samples were isolated from 15 day-old seedlings grown under long day conditions and harvested at 16:30 of the subjective day. Sequence reads were aligned with the well annotated rice genome (Japonica) to determine the frequency of reads matching each genomic regions. About 5 million clean reads per sample were obtained which matched perfectly with about 19 000–20 000 genes per sample (Table S1). In SY63, 1955 and 217 genes were up-regulated and 1559 and 650 genes down-regulated compared with MH63 and ZS97 (|log_2_Ratio≥1|), respectively, with False Discovery Rate (FDR)≤0.001 as an empirical cutoff value to provide a conservative assessment of differentially expressed genes (Figure 6A). Gene expression differed also greatly between MH63 and ZS97. This analysis suggests that the gene expression pattern in SY63 is closer to that of ZS97 than MH63. When compared to mid-parent expression, 619 and 471 genes in SY63 showed a lower (log_2_Ratio≤−1) and a higher (log_2_Ratio≥1) expression, respectively (Figure 6B). These differentially expressed genes displayed therefore a nonadditive effect in the hybrid. More detailed analysis revealed that the expression of 129 genes was lower (log_2_Ratio≤−1) than the low-parent, while 298 showed a higher expression (log_2_Ratio≥1) compared to the high-parent (Figure 6B) (Table S2). These genes therefore displayed underdominance and overdominance expression, respectively.

Comparison of transcript abundance between SY63, FR and FU revealed that many genes were up- or downregulated by OsHDT1 in the hybrid (Figure 6C). The *OsHDT1* over-expression (FU) altered more genes than the RNAi (FR). In FU, more genes showed up-regulation (totally 440) than down-regulation (164) compared to SY63, while in FR, 112 and 109 genes were induced and repressed, respectively (Table S3). Analysis of affected genes revealed that relatively higher proportions of genes involved in phenylpropanoid (e.g. flavoinoid) biosynthesis pathway were affected in both FU and FR plants, compared to other metabolic or biochemical pathways (Table 2, Table 3). Recent data have shown that the mutation of *OsGI* affects accumulation of transcripts and metabolites in the phenylpropanoid metabolite pathway [28]. The present data suggested that the effect of OsHDT1 on phenylpropanoid pathway genes might be achieved through regulation of *OsGI*. Few genes were affected in both FU and FR plants. Only 4 genes showed a FU>SY>FR profile, among which one is *OsHDT1* itself, reflecting the effect of transgene expression (Table S4). More than 20% of the affected genes by altered *OsHDT1* expression were nonadditive genes (SY63 > or < mid parent) (Figure 6C, Table S5).

**Table 2:** Pathway classification of differentially expressed genes in *OsHDT1* over-expression hybrid.

Pathway	DEGs with pathway annotation (291)	All genes with pathway annotation (16810)	P-value	Q-value	Pathway ID
Biosynthesis of phenylpropanoids	41 (14.09%)	866 (5.15%)	1.445e-08	1.445e-06	ko01061
Flavonoid biosynthesis	24 (8.25%)	397 (2.36%)	2.475e-07	1.238e-05	ko00941
Nitrogen metabolism	8 (2.75%)	118 (0.7%)	0.001	0.019	ko00910
Biosynthesis of terpenoids and steroids	17 (5.84%)	430 (2.56%)	0.002	0.026	ko01062
Cyanoamino acid metabolism	9 (3.09%)	183 (1.09%)	0.006	0.067	ko00460
Biosynthesis of alkaloids derived from ornithine, lysine and nicotinic acid	10 (3.44%)	264 (1.57%)	0.008	0.086	ko01064
Cysteine and methionine metabolism	10 (3.44%)	276 (1.64%)	0.012	0.090	ko00270
Glycolysis / Gluconeogenesis	8 (2.75%)	185 (1.1%)	0.019	0.120	ko00010
Amino sugar and nucleotide sugar metabolism	8 (2.75%)	189 (1.12%)	0.021	0.126	ko00520
Starch and sucrose metabolism	13 (4.47%)	429 (2.55%)	0.048	0.228	ko00500
Metabolism of xenobiotics by cytochrome P450	5 (1.72%)	149 (0.89%)	0.053	0.236	ko00980
Glycine, serine and threonine metabolism	4 (1.37%)	79 (0.47%)	0.054	0.236	ko00260
Linoleic acid metabolism	3 (1.03%)	103 (0.61%)	0.116	0.397	ko00591
Carbon fixation in photosynthetic organisms	4 (1.37%)	103 (0.61%)	0.116	0.397	ko00710
Steroid biosynthesis	3 (1.03%)	73 (0.43%)	0.145	0.426	ko00100
Spliceosome	10 (3.44%)	511 (3.04%)	0.441	0.689	ko03040
Plant-pathogen interaction	28 (9.62%)	2224 (13.23%)	0.969	0.979	ko04626

**Table 3:** Pathway classification of differentially expressed genes in *OsHDT1* RNAi hybrid.

Pathway	DEGs with pathway annotation (124)	All genes with pathway annotation (16810)	P-value	Q-value	Pathway ID
Linoleic acid metabolism	3 (2.42%)	103 (0.61%)	0.009	0.591	ko00591
Biosynthesis of phenylpropanoids	12 (9.68%)	866 (5.15%)	0.019	0.623	ko01061
Flavonoid biosynthesis	7 (5.65%)	397 (2.36%)	0.037	0.623	ko00941
Nitrogen metabolism	3 (2.42%)	118 (0.7%)	0.066	0.625	ko00910
Glycine, serine and threonine metabolism	2 (1.61%)	79 (0.47%)	0.128	0.625	ko00260
Cysteine and methionine metabolism	4 (3.23%)	276 (1.64%)	0.172	0.625	ko00270
Phenylpropanoid biosynthesis	7 (5.65%)	629 (3.74%)	0.225	0.625	ko00940
Starch and sucrose metabolism	5 (4.03%)	429 (2.55%)	0.248	0.625	ko00500
Biosynthesis of alkaloids derived from ornithine, lysine and nicotinic acid	3 (2.42%)	264 (1.57%)	0.343	0.676	ko01064
Plant-pathogen interaction	13 (10.48%)	2224 (13.23%)	0.685	0.785	ko04626
Spliceosome	3 (2.42%)	511 (3.04%)	0.769	0.826	ko03040

**Figure 7:**
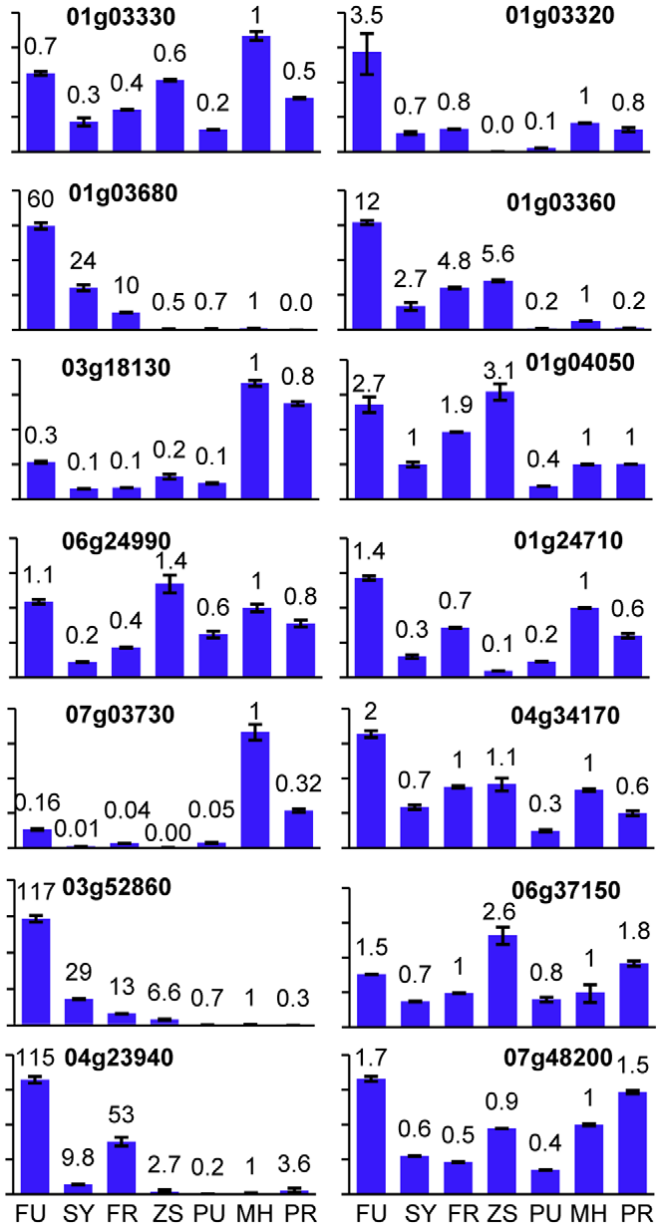
Different effects of *OsHDT1* in hybrid and parent. A selection of 14 genes was analyzed by quantitative RT-PCR in the indicated genotypes: PU, *OsHDT1*-over-expression in MH63; MH, MH63; PR, *OsHDT1* RNAi in MH63; FU, *OsHDT1*-over-expression in SY63; SY, SY63; FR, *OsHDT1* RNAi in SY63. Left panels: genes showing nonadditive expression, right panels, genes showing additive expression in the hybrid.

To check whether altered *OsHDT1* expression affected the additive and nonadditive genes similarly in the parent line, qRT-PCR analysis of a selection of 14 genes (7 showing nonadditive (*SY* > or < mid parent), the others showing additive (SY = mid parent) expression in the hybrid) was performed to compare FU, FR, SY63, ZS97, MH63, PU and PR. The results confirmed the digital expression analysis data and revealed that the effects of altered *OsHDT1* expression on these genes in the hybrid were different in MH63 background (Figure 7). For instance, most of the tested genes were induced by *OsHDT1* over-expression in the hybrid, but they were reduced or not clearly affected in the parent line. These observations indicate that altered OsHDT1 levels differentially regulate gene expression in hybrid than in the parent.

## Discussion

### *OsHDT1* function in histone deacetylation

OsHDT1 belongs to the plant-specific HD2 subfamily of histone deacetylases. The first member of the HD2 family was identified from maize, which is tightly bound to chromatin, located in the nucleolus [12]. Arabidopsis HDT1 is also localized in the nucleolus [13], [16]. Unlike AtHDT1 and the maize HD2, OsHDT1 was found to be localized in euchromatin regions (Figure 1E). As *OsHDT1* phylogenetically diverges from AtHDT1 (Figure 1A), the two proteins may have different functions. The euchromatic localization of OsHDT1 is consistent with the effect of its over-expression and RNAi on gene expression. AtHDT1 has been shown to be required for the post-embryonic establishment of nucleolar dominance that is an epigenetic phenomenon in plant and animal genetic hybrids and describes the expression of ribosomal RNA (rRNA) genes inherited from only one progenitor due to the silencing of the other progenitor's rRNA genes [13], [16]. Although a number of HD2 members have been studied, the biochemical function as histone deacetylases of this small protein family is not determined. Our data suggest that OsHDT1 may be involved in histone deacetylation on a subset of genes, as over-expression of *OsHDT1* led to decreases of histone acetylation on flowering repressor genes in both parent and hybrid backgrounds. Increased OsHDT1 promoted deacetylation that correlated with the repression of *OsGI* and *Hd1* and early flowering in the hybrid. The effect of OsHDT1 seemed to occur at a higher acetylation phase of target genes (Figure 5), suggesting that OsHDT1 might be involved in circadian oscillation of histone acetylation and gene expression. In addition, *OsHDT1* expression exhibited also a circadian oscillation and was sensitive to photoperiods (Figure 1C). These observations suggest a possible function of the protein in circadian regulation of histone deacetylation and gene expression. However, it is not known at this stage whether OsHDT1 was directly involved in the deacetylation or through an indirect mechanism.

### OsHDT1 function as a *trans*-acting regulator of hybrid differential gene expression

Our data show that increased OsHDT1 could repress nonadditive up-regulation of flower repressor genes in the hybrid rice. Nonadditive or differential gene expression may be a basis for the overdominance hypothesis to explain heterosis. The evidence for overdominance gene action, however, has been limited so far. Our observation of overdominance expression of flowering time genes in SY63 provides clear evidence for overdominance gene action (Figure 3, Figure 4). Flowering time is related to the success of hybrid reproduction. In rice and tomato, single genes involved in flowering time control have been demonstrated to be related to heterosis [23], [29]. Under long day conditions, the flowering time differs considerably between the MH63 and ZS97, whereas the flowering time in SY63 is close to that of MH63 (Figure 2D). Thus, the flowering time is a nonadditive phenotype, which is resulted from a complex interaction between a set of flowering regulatory genes in the hybrid (Figure 3A). It is shown that the mutation of *OsGI* increases *Ehd1* expression under long day condition [25]. Therefore, the nonadditive up-regulation (or overdominance) of *OsGI* and *Hd1* observed in the hybrid likely contributed to the below mid-parent expression of *Ehd1*, *RFT1* and *Hd3a* under long day conditions (Figure 3B). Reduction of the nonadditive increase of *Hd1* and *OsGI* by *OsHDT1* over-expression and the early flowering phenotype indicates that the “late flowering overdominance” in SY63 can be suppressed by elevated OsHDT1. These data together with the effects of altered *OsHDT1* expression on other nonadditive genes provide direct evidence of regulation of nonadditive or differential gene expression in hybrid by a *trans*-acting factor.

It is hypothesized that differential gene expression in hybrid may be responsible for heterosis [30], [31]. Gene expression patterns in hybrid plants have been reported [2], [6], [32]. The present study using digital expression analysis revealed characteristic expression patterns in the elite hybrid rice SY63 (Figure 6). From 2754 comparable genes (FDR≤0.001), about 40% (1090) were non-additively expressed (|log_2_Ratio≥1|) in the hybrid. Gene expression in SY63 is less deviated from ZS97 than MH63, suggesting more contribution to the hybrid gene expression from the ZS97 genome than from MH63. Alternatively, the ZS97 genome may mostly influence the expression of MH63 genome in the hybrid. As previous results in rice suggest no significant parent-of-origin effect for the action of parental alleles in hybrid [6], interaction between the two parental genomes for gene expression is most likely to be mediated by *trans*-acting factors that may be predominantly from the ZS97 origin. Differential gene expression in hybrid is suggested to be the result of variation in *cis*-acting elements or *trans*-acting factors between parents [2], [4], [5]. It is suggested that nonadditive expression in hybrid may be controlled by *trans*-acting factors, while additive expression by both *cis*-acting promoter elements and *trans*-acting transcription factors. The observations that *OsHDT1* over-expression and RNAi affected both additive and nonadditive gene expression in the hybrid support the hypothesis that *trans*-acting factors control both additive and nonadditive variations.

In addition, the present data showed that the expression of a significant number of genes displayed over- or underdominance in the hybrid. Over- or underdominance in gene expression has been explained by nonallelic control of transcript accumulation [2]. The effects of altered OsHDT1 levels on overdominance gene expression are in favour of this hypothesis.

Taken together, this work provided evidence of regulation and action of overdominance genes in flowering time control in the hybrid and revealed that OsHDT1 level was important for a subset of differentially expressed genes including flowering time genes in the hybrid. The data suggest that OsHDT1 may have a function in parental genome interaction for gene expression in the hybrid.

## Materials and Methods

### Plant Materials and Growth Conditions

The rice varieties MH63 (male) and ZS97 (female) (*Oryza sativa L.spp.indica*) and the hybrid SY63 were studied in this study. MH63 was used for transgenic plant production. The field conditions for rice cultivation are described previously [23]. For rice cultivation in growth chambers, rice seeds were sown in pots and rice plants were grown in a Versatile Environmental Test Chamber (MLR-351H, SANYO) with light intensity set at 15,000 lx, temperature at 30°C during the light period and 25°C during the dark period, and humidity at 70%, under either long day (15 h light/ 9 h dark) or short day conditions (9 h light /15 h dark). For *in vitro* culture, rice seeds were germinated and the seedlings were grown on 1/2 MS medium under a 15 h/light at 30°C and 9 h/dark cycle at 25°C for 15 days.

### Phylogenetic Analysis

For sequence analysis, the HD2 family protein sequences downloaded from plant ChromDB database (www.chromdb.org/) were used for sequence alignment and phylogeny. Phylogeny reconstruction of HD2 protein sequence alignments was performed by MEGA 3.1 [33] using the neighbor-joining method.

### Vector Construction and Rice Transformation

To make *OsHDT1* RNAi construct, the vector pDS1301 was used [34]. A 448-bp cDNA fragment of *OsHDT1* was amplified using primers RNAi-F and RNAi-R (listed in Table S6). PCR products were digested with *Kpn* I/*Bam*H I and *Sac* I/*Spe* I respectively and inserted downstream to the CaMV 35S promoter in pDS1301.

The over-expression vector was constructed by directionally inserting the full cDNA sequence amplified with the primer set HDT1-F and HDT1-R (Table S6) (digested with *Kpn* I/*Bam*H I) into the binary vector pU1301 under the control of the maize ubiquitin promoter [35].

For OsHDT1-3×FLAG fusion, the *OsHDT1* full-length cDNA without stop codon was inserted downstream the 3×FLAG tag in a modified pU1301 vector. *Agrobacterium tumefaciens* (strain EHA105)-mediated transformation of rice plants was conducted according to a published protocol [36].

### Antibody Production and Affinity Purification

Anti-OsHDT1 antibody was raised against proteins expressed in *E. coli*. Briefly, *OsHDT1* full length cDNA was cloned in pET28a vector with an N-terminal 6×His tag. The plasmid was transformed into BL21-DE3 cells grown at 37°C. When cultures reached an A600 of 0.8, protein expression was induced by addition of 0.1 mM IPTG and cultures were incubated for additional 10 hrs at 20°C. Cells collected by centrifugation at 5,000 g for 10 min at 4°C were resuspended in 10 ml Phosphate Buffered Saline (PBS), sonicated to lyse the cells and centrifuged. Soluble proteins were purified by B-PER® 6×HIS Spin Purification Kit (Thermo). Approximately 2 mg of purified OsHDT1 protein were subjected to SDS-PAGE and excised from the gel. Gel slices were grinded in liquid nitrogen and resuspended in PBS for antibody production in rabbits. The affinity purification of anti-OsHDT1 antibody was performed as described previously [16].

### Nuclear Localisation

For FLAG-tagged OsHDT1 immunostaining, 10 days old rice seedling nuclei were isolated and fixed in 4% paraformaldehyde in PBS as described previously [37]. Nuclei were incubated overnight at 4°C with the following polyclonal antibodies: the mouse anti-FLAG (Sigma, 1∶300 working dilution) and rabbit anti-OsHDT1 (1∶30). Protein-antibody complexes were detected using the Alexa Fluor 594–coupled goat anti-mouse and Fluor 488–coupled goat anti-rabbit second antibodies (Molecular Probes, 1∶200).

### Expression Analysis by Northern Blot, RT-PCR and Quantitative PCR

For flowering time gene expression analysis, after growing under long day conditions (15 h light/9 h dark) for 21 days, half of the plants received a short-day treatment in a different chamber, and the other half remained under long-day conditions. After entraining for 14 days, young leaves were simultaneously harvested from three different plants for each treatment, and stored in liquid nitrogen. The samples were collected in 4 h intervals, starting at 08:30 for a total of 48 h. After RNA samples extracted using TRIzol (Invitrogen) according to standard protocols.

For quantitative PCR (Applied Biosystems 7500), primers were designed by PRIMER EXPRESS 2.0 software (PE Applied Biosystems) to amplify 90- to 150-bp products. Products were measured by SYBR green fluorescence (Takara) in 25 µl reactions, all primers were annealed at 58°C. In all qRT-PCR, rice actin transcripts were measured as internal references. Data analyses with 2^−ΔΔCt^ method were performed as described [38]. For semi-quantitative RT-PCR analyses were performed as described. For Northern blots, an *OsHDT1* cDNA fragment was used as probe. RT and real-time PCR primers were listed in Table S6.

### Western Blot Analysis

For OsHDT1 detection, protein samples were isolated from 40 day-old rice leaves grown under natural long day conditions. Total protein was extracted from rice leaves as described [39]. Western blot analysis was performed with anti-HDT (1∶1000 working dilution) as primary antibody according to standard protocols. For histone modifications, rice leaf histone extraction was performed as described [40]. After blocked with 2% BSA in PBS (pH 7.5), the membrane was incubated overnight with primary antibodies Anti-acetyl-Histone H3 (06-599, Millipore), Anti-acetyl-Histone H4 (06-866, Millipore), Anti-acetyl-Histone H4K16 (07-329, Millipore), Anti-acetyl-Histone H4K5 (ab51997, Abacm) and Anti-H3 (ab1791, Abcam) in a 1∶5000 dilution at room temperature. After three washes (10 min each) the secondary antibody goat anti-rabbit IgG (SouthernBiotech, USA) was used at 1∶10000. Visualization was performed by using the SuperSignal® West Pico Kit (Pierce, USA) according to the manufacturer's instructions.

### Southern Blot Analysis

Genomic DNA was extracted from rice leaves. A total of 4 mg of DNA was digested with *Kpn* I and *Bam*H I overnight, separated on 1% (w/v) agarose gel, then transferred to a nylon membrane and hybridized with hygromycin gene probe according to standard protocols.

### Chromatin Immunoprecipitation (ChIP) Assay

Leaves from 35 days old rice plants cultured in growth chambers were used for chromatin immunoprecipitation assays, and the methods were performed as described [41]. The antibody used for immunoprecipitated was Anti-acetyl-Histone H4 (06-866, Millipore). Precipitated DNA was re-suspended in 100 µl TE (10 mM Tris/1 mM EDTA, pH 8.0) for quantitative PCR with the rice actin gene as control. PCR primers for CHIP were listed in Table S6.

### Digital Expression Analysis

For digital expression analysis, 15 day-old transgenic and wild type seedlings grown on 1/2 MS medium were harvested for RNA extraction using TRIzol (Invitrogen) as described by the manufacturer. The digital expression analysis was performed by Beijing Genomics Institute using the following standardized procedure: The main reagents and supplies are Illumina Gene Expression Sample Prep Kit and Illumina Sequencing Chip (flowcell), and the main instruments are Illumina Cluster Station and Illumina HiSeq™ 2000 System. Six µg of total RNA were to purify mRNA by using oligo(dT) magnetic beads, and the mRNAs were used to synthesize the first and second-strand cDNA using oligo(dT) as primer. The bead-bound cDNAs were subsequently digested with restriction enzyme NlaIII, which recognizes and cuts off the CATG sites. The fragments apart from the 3′ cDNA fragments connected to oligo(dT) beads were washed away and the Illumina adaptor 1 was ligated to the sticky 5′ end of the digested bead-bound cDNA fragments. The junction of Illumina adaptor 1 and CATG site is the recognition site of MmeI, which is a type of endonuclease with separated recognition sites and digestion sites. It cuts at 17 bp downstream of the CATG site, producing tags with adaptor 1. After removing 3′ fragments with magnetic beads precipitation, Illumina adaptor 2 was ligated to the 3′ ends of tags, acquiring tags with different adaptors of both ends to form a tag library. After 15 cycles of linear PCR amplification, 95 bp fragments were purified by 6% TBE PAGE Gel electrophoresis. After denaturation, the single-chain molecules were fixed onto the Illumina Sequencing Chip (flowcell). Each molecule grows into a single-molecule cluster sequencing template through in situ amplification. Then four types of nucleotides which are labeled by four colors were added, and sequencing was performed with the method of sequencing by synthesis (SBS). Each tunnel generated millions of raw reads with sequencing length of 35 bp.

All clean tags were mapped to the reference sequences (ftp://ftp.plantbiology.msu.edu/pub/data/Eukaryotic_Projects/o_sativa/annotation_dbs/pseudomolecules/version_6.1/all.dir/all.cDNA) and only 1 bp mismatch is considered. Clean tags mapped to reference sequences from multiple genes were filtered. Remainder clean tags were designed as unambiguous clean tags. The number of unambiguous clean tags for each gene was calculated and then normalized to TPM (number of transcripts per million clean tags) [26], [27].

The significance of digital gene expression was determined using a published statistical model as descried previously [42]. The significance of gene expression difference was judged by using “FDR≤0.001 and the absolute value of log2Ratio≥1” [43] as the threshold. FDR (False Discovery Rate) is a method to determine the threshold of P-value in multiple test and analysis through manipulating the FDR value. The original data set is deposited in the National Institutes of Health Gene Expression Omnibus database under accession number GSE27240.

### Pathway Enrichment Analysis for Differentially Expressed Genes

All differentially expressed genes (DEGs) were mapped to terms in KEGG public pathway-related database (http://www.genome.jp/kegg/). Pathway enrichment analysis applies hypergeometric test identifying significantly enriched metabolic or signal transduction pathways in differentially expressed genes comparing with the whole genome background.

## Supporting Information

Figure S1 **Detection of OsHDT1-Flag in transgenic lines by Western blots. Arrows indicate positions of the HDT1-Flag protein.**
(TIF)

Figure S2 **Copy number and expression analysis of** ***OsHDT1*** **transgenic lines.** A. Schematic representation of the gene structure and cDNA sequence of *OsHDT1*. The black boxes indicate the exons, the fold lines indicate the introns and the white boxes indicate the UTR. The DNA segment used to construct the RNAi vector is indicated. B. Copy number of *OsHDT1* transgenes detected by Southern blot hybridization. The total DNA was cut by *Kpn* I and *Bam*H I respectively. The blots were probed by the hygromycin gene of the vector. C. *OsHDT1* expression analysis in overexpression and RNAi transgenic plants compared to wild type MH63 by Northern blots (upper) and qRT-PCR respectively.
(TIF)

Figure S3 **Comparison of histone modifications.** Histones isolated from the 75 days old rice leaf using the antibodies of different histone modification modules indicated on the left. Gel staining of loaded histones is shown at the bottom. ZS, ZS97; PU, *OsHDT1*-over-expression in MH63; MH, MH63; PR, *OsHDT1* RNAi in MH63; FU, *OsHDT1*-over-expression in SY63; SY, SY63; FR, *OsHDT1* RNAi in SY63.
(TIF)

Figure S4 **Diurnal genes expression in different genotypes under long day conditions.**
(TIF)

Table S1 **Summary of statistics for the RNA sequencing results.**
(DOCX)

Table S2 **Genes showing SY63>high-parent or SY63<low-parent expression.**
(DOCX)

Table S3 **Differential expressed genes between transgenic and wild type hybrids.**
(DOCX)

Table S4 **Genes showing FU>SY63>FR expression.**
(DOCX)

Table S5 **Differentially expressed genes affected by** ***OsHDT1*** **in SY63 background.**
(DOCX)

Table S6 **Primers used in this study.**
(DOCX)
