# Supplementary figures and images for: Altered Levels of Histone Deacetylase OsHDT1 Affect Differential Gene Expression Patterns in Hybrid Rice
Source: PLoS One. 2011 Jul 8;6(7):e21789. doi: 10.1371/journal.pone.0021789 (PMC3132746; doi:10.1371/journal.pone.0021789)

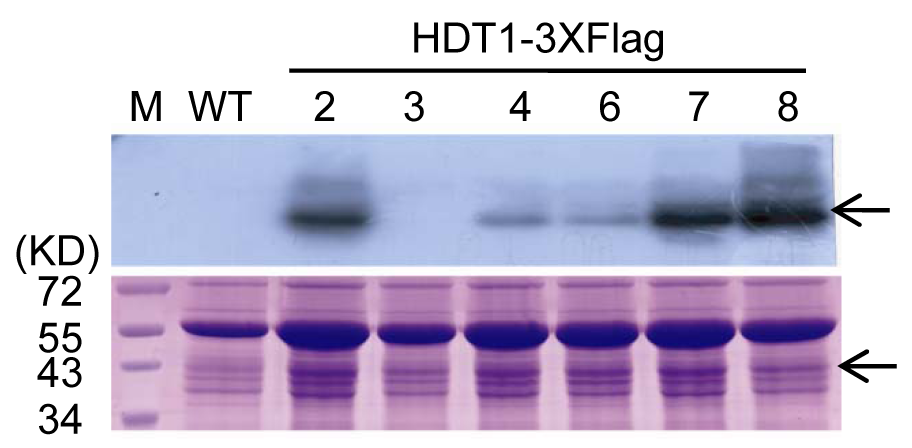

Supplement: Figure S1 — Detection of OsHDT1-Flag in transgenic lines by Western blots. Arrows indicate positions of the HDT1-Flag protein. (TIF) [file pone.0021789.s001.tif]

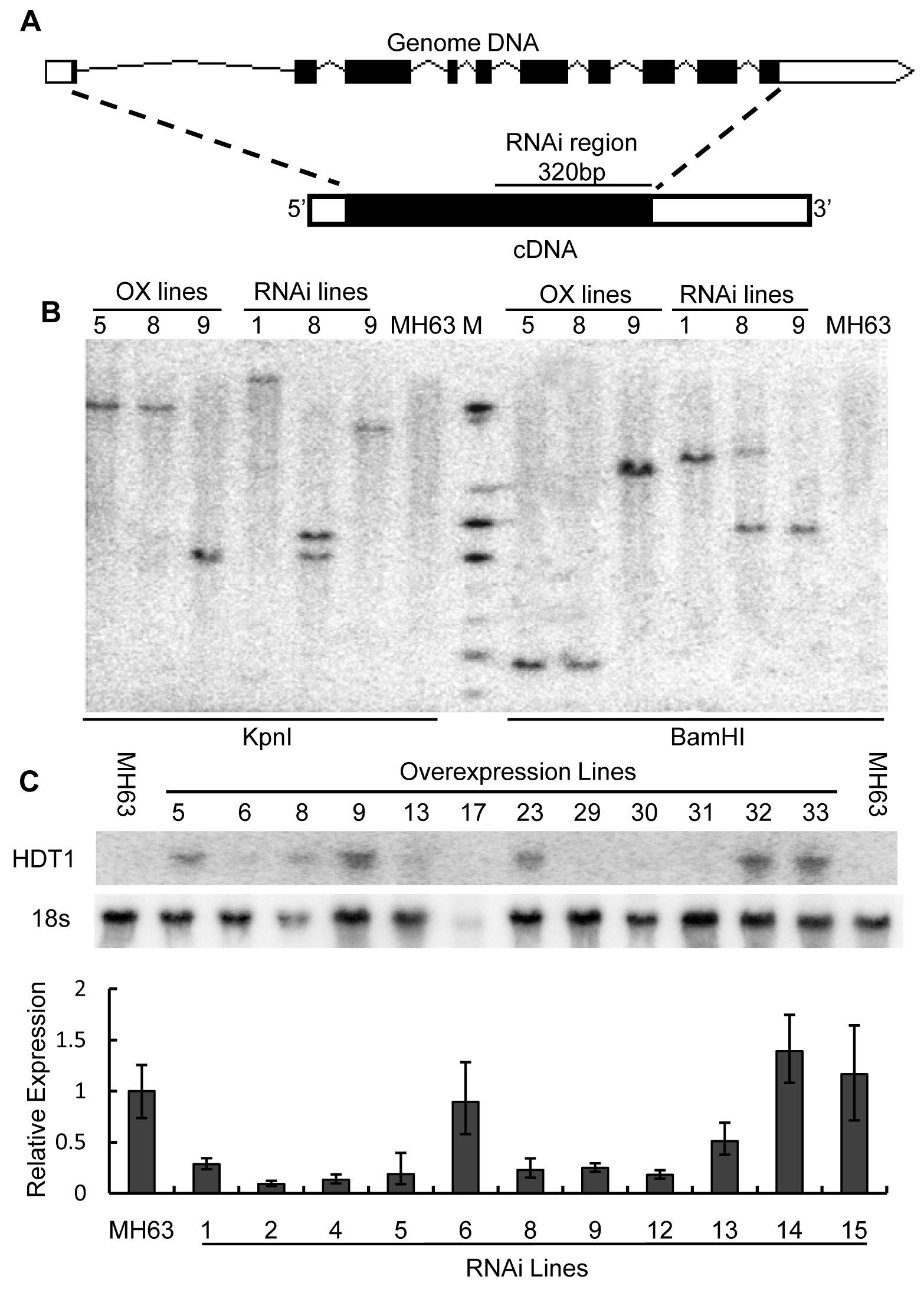

Supplement: Figure S2 — Copy number and expression analysis of OsHDT1 transgenic lines. A. Schematic representation of the gene structure and cDNA sequence of OsHDT1. The black boxes indicate the exons, the fold lines indicate the introns and the white boxes indicate the UTR. The DNA segment used to construct the RNAi vector is indicated. B. Copy number of OsHDT1 transgenes detected by Southern blot hybridization. The total DNA was cut by Kpn I and BamH I respectively. The blots were probed by the hygromycin gene of the vector. C. OsHDT1 expression analysis in overexpression and RNAi transgenic plants compared to wild type MH63 by Northern blots (upper) and qRT-PCR respectively. (TIF) [file pone.0021789.s002.tif]

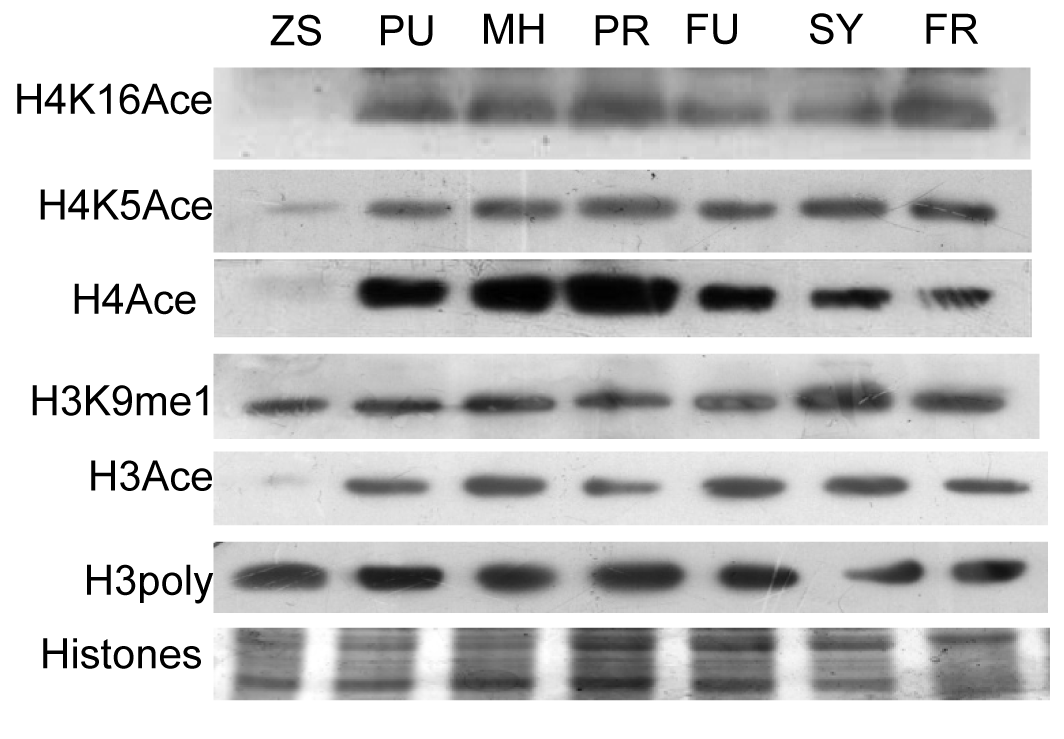

Supplement: Figure S3 — Comparison of histone modifications. Histones isolated from the 75 days old rice leaf using the antibodies of different histone modification modules indicated on the left. Gel staining of loaded histones is shown at the bottom. ZS, ZS97; PU, OsHDT1-over-expression in MH63; MH, MH63; PR, OsHDT1 RNAi in MH63; FU, OsHDT1-over-expression in SY63; SY, SY63; FR, OsHDT1 RNAi in SY63. (TIF) [file pone.0021789.s003.tif]

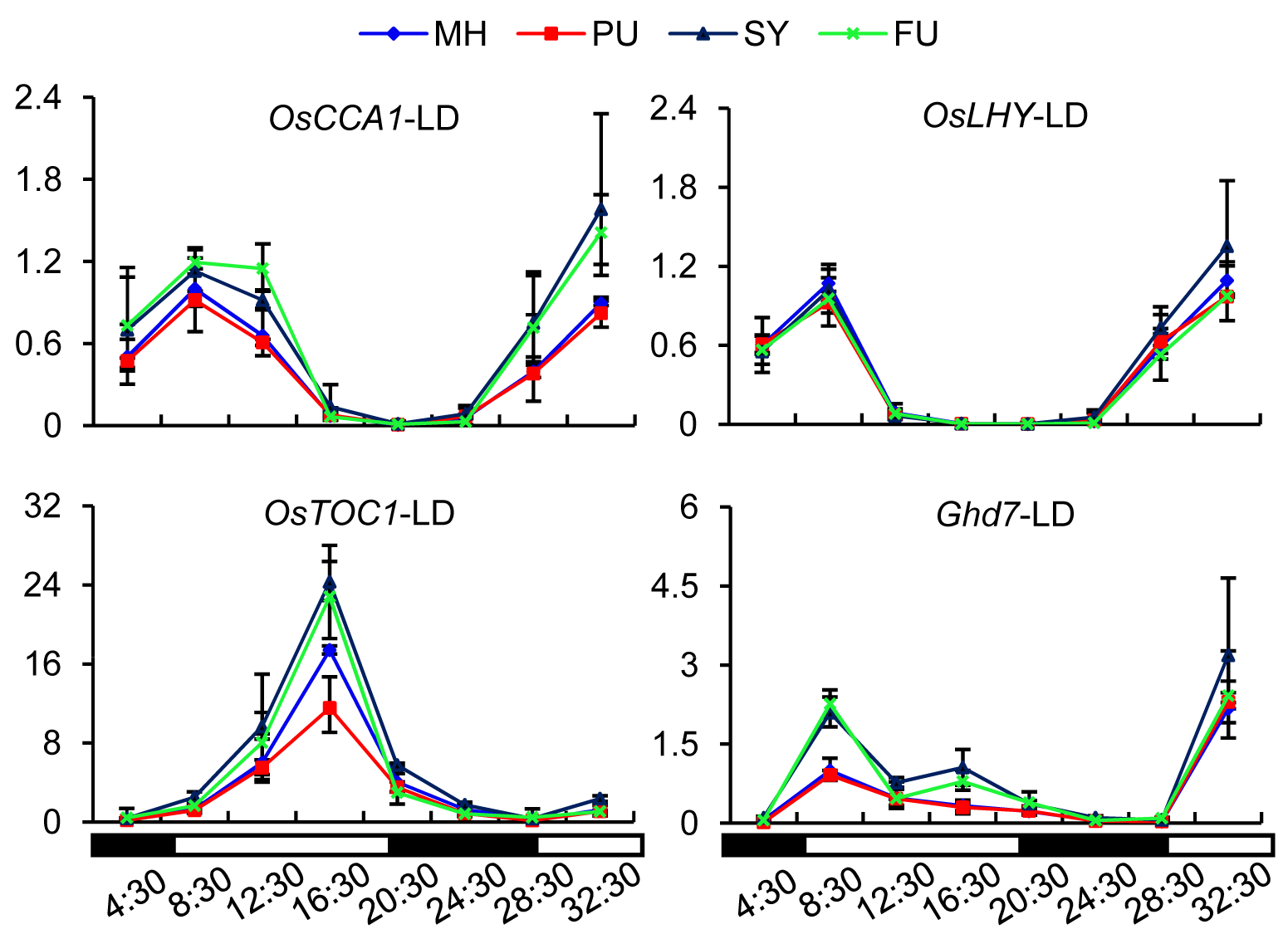

Supplement: Figure S4 — Diurnal genes expression in different genotypes under long day conditions. (TIF) [file pone.0021789.s004.tif]
