# Supplementary material for: Altered Levels of Histone Deacetylase OsHDT1 Affect Differential Gene Expression Patterns in Hybrid Rice
Source: PLoS One. 2011 Jul 8;6(7):e21789. doi: 10.1371/journal.pone.0021789 (PMC3132746; doi:10.1371/journal.pone.0021789)
Supplement: Table S1 — Summary of statistics for the RNA sequencing results. (DOCX) [file pone.0021789.s005.docx]

**Table S1.** Summary of statistics for the RNA sequencing results

|  | FU | FR | SY | MH | ZS |
| --- | --- | --- | --- | --- | --- |
| Total clean tags | 4,821,637 | 5,011,951 | 4,857,063 | 5,022,081 | 4,957,343 |
| Distinct clean tags | 178,230 | 164,891 | 166,174 | 119,015 | 145,771 |
| Perfect match genes#* | 20,170 | 19,782 | 19,832 | 19,041 | 19,081 |

#From a total of 56797 reference genes, 52845(93.04%) have at least a CATG Site.

* Tags matching to more than one gene were deleted
