# Supplementary material for: Altered Levels of Histone Deacetylase OsHDT1 Affect Differential Gene Expression Patterns in Hybrid Rice
Source: PLoS One. 2011 Jul 8;6(7):e21789. doi: 10.1371/journal.pone.0021789 (PMC3132746; doi:10.1371/journal.pone.0021789)
Supplement: Table S2 — Genes showing SY63>high-parent or SY63<low-parent expression. (DOCX) [file pone.0021789.s006.docx]

**Table S2.** Genes showing SY63>high-parent or SY63<low-parent expression

| **Gene**  **(LOC_Os)** | **TPM**  **-SY** | **TPM**  **-ZS** | **TPM**  **-MH** | **log_2_**  **(SY/mid)** | **Description** |  |
| --- | --- | --- | --- | --- | --- | --- |
| 09g38450 | 5.76 | 0.01 | 0.01 | 9.17 | expressed protein |  |
| 04g01690 | 5.56 | 0.01 | 0.01 | 9.12 | pyridoxal-dependent decarboxylase protein |  |
| 05g43780 | 4.74 | 0.01 | 0.01 | 8.89 | ESP1 |  |
| 09g38710 | 4.32 | 0.01 | 0.01 | 8.75 | HEAT repeat family protein |  |
| 10g36390 | 4.12 | 0.01 | 0.01 | 8.69 | monocopper oxidase |  |
| 01g42070 | 3.91 | 0.01 | 0.01 | 8.61 | kinesin motor domain containing protein |  |
| 12g13950 | 3.71 | 0.01 | 0.01 | 8.54 | POLA2 -DNA polymerase alpha complex subunit |  |
| 12g27220 | 3.71 | 0.01 | 0.01 | 8.54 | transferase family protein |  |
| 03g53580 | 3.5 | 0.01 | 0.01 | 8.45 | expressed protein |  |
| 05g06840 | 3.5 | 0.01 | 0.01 | 8.45 | POLE2A - DNA polymerase epsilon complex subunit |  |
| 09g29210 | 3.29 | 0.01 | 0.01 | 8.36 | purine permease |  |
| 01g67100 | 3.09 | 0.01 | 0.01 | 8.27 | expressed protein |  |
| 03g07250 | 3.09 | 0.01 | 0.01 | 8.27 | cytochrome P450 |  |
| 02g28850 | 2.88 | 0.01 | 0.01 | 8.17 | Kinesin motor domain containing protein |  |
| 03g52794 | 2.88 | 0.01 | 0.01 | 8.17 | phosphatidylinositol kinase |  |
| 08g04190 | 2.88 | 0.01 | 0.01 | 8.17 | homeobox and START domains containing protein |  |
| 08g04240 | 2.88 | 0.01 | 0.01 | 8.17 | cysteine-rich repeat secretory protein 55 precursor |  |
| 02g51640 | 2.68 | 0.01 | 0.01 | 8.07 | expressed protein |  |
| 03g11680 | 2.68 | 0.01 | 0.01 | 8.07 | ZOS3-05 - C2H2 zinc finger protein |  |
| 11g01200 | 2.68 | 0.01 | 0.01 | 8.07 | senescence-induced receptor-like serine/threonine-protein kinase precursor |  |
| 03g52860 | 28 | 0.01 | 1 | 5.81 | lipoxygenase |  |
| 11g10590 | 8.65 | 0.4 | 0.01 | 5.43 | hypothetical protein |  |
| 01g67740 | 7.62 | 0.4 | 0.01 | 5.25 | chromosome segregation protein |  |
| 12g14440 | 424.7 | 15.33 | 9.56 | 5.09 | Jacalin-like lectin domain containing protein |  |
| 03g43910 | 13.38 | 0.01 | 0.8 | 5.06 | expressed protein |  |
| 01g01890 | 6.59 | 0.01 | 0.4 | 5.04 | expressed protein |  |
| 05g34170 | 9.47 | 0.61 | 0.01 | 4.98 | tubulin/FtsZ domain containing protein |  |
| 03g17164 | 5.56 | 0.4 | 0.01 | 4.80 | kinesin-related protein |  |
| 01g03680 | 27.38 | 0.61 | 1.39 | 4.78 | BBTI8 - Bowman-Birk type bran trypsin inhibitor precursor |  |
| 08g32600 | 5.35 | 0.4 | 0.01 | 4.74 | protein kinase domain containing protein |  |
| 01g64900 | 4.74 | 0.01 | 0.4 | 4.57 | HEAT repeat family protein |  |
| 03g46920 | 28.41 | 1.21 | 1.39 | 4.45 | expressed protein |  |
| 04g34240 | 10.91 | 1.01 | 0.01 | 4.45 | histone H3 |  |
| 12g39210 | 4.32 | 0.01 | 0.4 | 4.43 | G2/mitotic specific cyclin C13-1 |  |
| 06g06510 | 87.3 | 3.43 | 4.78 | 4.41 | histone H3 |  |
| 01g49200 | 29.03 | 2.22 | 0.6 | 4.37 | microtubule associated protein |  |
| 10g35580 | 8.24 | 0.81 | 0.01 | 4.36 | ATEB1A-like microtubule associated protein |  |
| 04g15840 | 4.12 | 0.4 | 0.01 | 4.36 | expansin precursor |  |
| 11g45990 | 18.32 | 0.81 | 1 | 4.35 | von Willebrand factor type A domain containing protein |  |
| 06g03710 | 8.03 | 0.4 | 0.4 | 4.33 | DELLA protein SLR1 |  |
| **Table S2. (Continued)** | | | | | | |
| **Gene**  **(LOC_Os)** | **TPM**  **-SY** | **TPM**  **-ZS** | **TPM**  **-MH** | **log_2_**  **(SY/mid)** | **Description** |  |
| 09g25380 | 11.32 | 0.4 | 0.8 | 4.24 | kinesin motor protein-related |  |
| 02g15810 | 12.56 | 0.4 | 1 | 4.17 | HMG1/2 |  |
| 04g15800 | 10.71 | 0.4 | 0.8 | 4.16 | expressed protein |  |
| 05g49920 | 3.5 | 0.01 | 0.4 | 4.13 | pentatricopeptide |  |
| 03g03630 | 3.5 | 0.4 | 0.01 | 4.13 | expressed protein |  |
| 09g36280 | 5.15 | 0.61 | 0.01 | 4.10 | glycosyl hydrolases family 17 |  |
| 03g07100 | 20.38 | 1.01 | 1.39 | 4.09 | Protease inhibitor/seed storage/LTP family protein precursor |  |
| 01g63500 | 4.94 | 0.01 | 0.6 | 4.04 | expressed protein |  |
| 06g03150 | 4.74 | 0.01 | 0.6 | 3.98 | expressed protein |  |
| 08g01100 | 26.77 | 2.42 | 1 | 3.98 | HMG1/2 |  |
| 04g40940 | 4.53 | 0.01 | 0.6 | 3.92 | mitotic spindle checkpoint protein MAD2 |  |
| 07g46540 | 5.97 | 0.81 | 0.01 | 3.90 | condensin complex subunit 1 |  |
| 02g08310 | 4.32 | 0.61 | 0.01 | 3.85 | tubby |  |
| 01g74146 | 4.32 | 0.01 | 0.6 | 3.85 | WD repeat-containing protein |  |
| 04g48760 | 4.32 | 0.01 | 0.6 | 3.85 | leucine-rich repeat family protein |  |
| 03g02290 | 18.53 | 1.61 | 1 | 3.83 | kinesin motor domain containing protein |  |
| 10g01570 | 12.76 | 0.61 | 1.19 | 3.83 | C-5 cytosine-specific DNA methylase |  |
| 04g59260 | 8.44 | 0.81 | 0.4 | 3.81 | peroxidase precursor |  |
| 05g39850 | 11.12 | 0.4 | 1.19 | 3.80 | MCM3 -Minichromosome maintenance complex subunit 3 |  |
| 11g35090 | 9.68 | 1.01 | 0.4 | 3.79 | kinesin motor domain containing protein |  |
| 03g52300 | 8.85 | 0.81 | 0.6 | 3.66 | SCP-like extracellular protein |  |
| 06g43220 | 4.94 | 0.01 | 0.8 | 3.63 | AP2 domain containing protein |  |
| 05g02300 | 30.68 | 5.04 | 0.01 | 3.61 | Histone H2A |  |
| 03g11540 | 19.35 | 1.21 | 1.99 | 3.60 | RPA1B -Single-stranded DNA binding complex subunit 1 |  |
| 09g37650 | 8.44 | 0.81 | 0.6 | 3.59 | flavin-containing monooxygenase family protein |  |
| 02g47150 | 15.65 | 0.81 | 1.79 | 3.59 | DNA topoisomerase 2 |  |
| 03g06670 | 22.65 | 1.61 | 2.19 | 3.58 | Histone H2A.Z |  |
| 10g42660 | 4.74 | 0.81 | 0.01 | 3.57 | expressed protein |  |
| 12g09700 | 8.03 | 1.01 | 0.4 | 3.52 | Jacalin-like lectin domain containing protein |  |
| 08g07080 | 4.53 | 0.81 | 0.01 | 3.5 | terpene synthase |  |
| 01g52610 | 3.29 | 0.61 | 0.01 | 3.46 | membrane associated DUF588 domain containing protein |  |
| 12g36220 | 127.0 | 20.78 | 2.79 | 3.44 | inhibitor I family protein |  |
| 04g47580 | 8.65 | 0.4 | 1.19 | 3.43 | G2/mitotic specific cyclin 2 |  |
| 03g51970 | 4.32 | 0.01 | 0.8 | 3.43 | growth-regulating factor |  |
| 12g06980 | 4.32 | 0.4 | 0.4 | 3.43 | SAP domain containing protein |  |
| 01g33040 | 7.41 | 1.41 | 0.01 | 3.40 | kinesin motor domain containing protein |  |
| 03g38010 | 4.12 | 0.4 | 0.4 | 3.36 | nuf2 family protein |  |
| 12g27350 | 19.56 | 1.01 | 2.79 | 3.36 | 10-deacetylbaccatin III 10-O-acetyltransferase |  |
| 07g23520 | 88.32 | 10.09 | 7.17 | 3.36 | expressed protein |  |
| 03g45760 | 9.68 | 0.81 | 1.19 | 3.28 | expressed protein |  |
| 04g50960 | 4.74 | 0.61 | 0.4 | 3.24 | expressed protein |  |
| **Table S2. (Continued)** | | | | | | |
| **Gene**  **(LOC_Os)** | **TPM**  **-SY** | **TPM**  **-ZS** | **TPM**  **-MH** | **log_2_**  **(SY/mid)** | **Description** |  |
| 07g04200 | 6.59 | 0.4 | 1 | 3.23 | bacterial transferase hexapeptide domain containing protein |  |
| 11g29380 | 12.15 | 1.41 | 1.19 | 3.22 | MCM2 - Minichromosome maintenance complex subunit 2 |  |
| 02g50910 | 6.38 | 0.01 | 1.39 | 3.19 | expressed protein |  |
| 12g39980 | 6.18 | 0.01 | 1.39 | 3.14 | kinesin motor domain containing protein |  |
| 12g02470 | 15.85 | 3.03 | 0.6 | 3.14 | OsWRKY65 |  |
| 04g43300 | 7 | 0.4 | 1.19 | 3.13 | BRCA1 C Terminus domain containing protein |  |
| 12g44090 | 7 | 0.81 | 0.8 | 3.13 | leucine-rich repeat family protein |  |
| 05g06140 | 12.97 | 1.01 | 1.99 | 3.11 | lipase |  |
| 05g29010 | 4.32 | 0.01 | 1 | 3.11 | POLA4 -DNA polymerase alpha complex subunit |  |
| 01g70560 | 10.91 | 1.61 | 1 | 3.07 | expressed protein |  |
| 08g36570 | 4.12 | 0.4 | 0.6 | 3.04 | F-box protein |  |
| 06g02530 | 13.79 | 0.01 | 3.39 | 3.02 | expressed protein |  |
| 08g40170 | 12.76 | 1.21 | 1.99 | 3.00 | cyclin-dependent kinase B2-1 |  |
| 06g39780 | 4.74 | 1.21 | 0.01 | 2.98 | cytochrome P450 |  |
| 08g44420 | 12.56 | 1.82 | 1.39 | 2.97 | kinesin-related protein |  |
| 05g31040 | 3.91 | 0.4 | 0.6 | 2.97 | cytokinin dehydrogenase precursor |  |
| 05g38480 | 3.91 | 0.4 | 0.6 | 2.97 | kinesin motor domain containing protein |  |
| 05g33030 | 11.53 | 1.41 | 1.59 | 2.94 | kinesin motor domain containing protein |  |
| 11g46000 | 30.68 | 4.84 | 3.19 | 2.94 | von Willebrand factor type A domain containing protein |  |
| 06g43770 | 5.35 | 0.01 | 1.39 | 2.93 | expressed protein |  |
| 02g05930 | 4.53 | 1.21 | 0.01 | 2.92 | phytosulfokine receptor precursor |  |
| 02g41904 | 67.12 | 2.42 | 15.33 | 2.91 | DEF7 - Defensin and Defensin-like DEFL family |  |
| 05g41390 | 5.97 | 0.61 | 1 | 2.90 | G2/mitotic specific cyclin S13-6 |  |
| 07g32660 | 5.15 | 0.4 | 1 | 2.88 | monocopper oxidase |  |
| 03g39020 | 8.03 | 1.41 | 0.8 | 2.87 | Kinesin motor domain containing protein |  |
| 01g12860 | 4.32 | 1.21 | 0.01 | 2.85 | MYB family transcription factor |  |
| 03g44890 | 4.32 | 0.01 | 1.19 | 2.85 | anthranilate phosphoribosyltransferase |  |
| 04g41900 | 34.18 | 4.44 | 5.58 | 2.77 | expressed protein |  |
| 07g32390 | 10.91 | 1.82 | 1.39 | 2.77 | targeting protein-related |  |
| 10g40810 | 11.53 | 1.82 | 1.59 | 2.76 | GATA zinc finger domain containing protein |  |
| 10g04620 | 4.74 | 1.41 | 0.01 | 2.76 | OsPOP20 - Putative Prolyl Oligopeptidase homologue |  |
| 09g31430 | 9.26 | 2.82 | 0.01 | 2.73 | Os9bglu30 - beta-glucosidase |  |
| 01g67110 | 13.79 | 1.41 | 2.79 | 2.72 | expressed protein |  |
| 04g27670 | 7.82 | 1.61 | 0.8 | 2.70 | terpene synthase family, metal binding domain containing |  |
| 10g37850 | 19.56 | 3.63 | 2.39 | 2.70 | armadillo |  |
| 07g44830 | 4.53 | 1.41 | 0.01 | 2.69 | proline-rich family protein |  |
| 06g45990 | 44.68 | 8.88 | 4.98 | 2.69 | patellin-5 |  |
| 07g05190 | 7 | 0.81 | 1.39 | 2.67 | leucine-rich repeat family protein |  |
| 07g08500 | 17.09 | 2.82 | 2.59 | 2.66 | C-5 cytosine-specific DNA methylase |  |
| 01g36390 | 10.09 | 0.61 | 2.59 | 2.66 | MCM4 - Minichromosome maintenance complex subunit 4 |  |
| 03g44900 | 18.74 | 4.64 | 1.39 | 2.64 | CCR4-NOT transcription factor |  |
| **Table S2. (Continued)** | | | | | | |
| **Gene**  **(LOC_Os)** | **TPM**  **-SY** | **TPM**  **-ZS** | **TPM**  **-MH** | **log_2_**  **(SY/mid)** | **Description** |  |
| 12g36210 | 114.9 | 35.1 | 2.19 | 2.63 | inhibitor I family protein |  |
| 02g56490 | 4.94 | 1.01 | 0.6 | 2.63 | expressed protein |  |
| 03g53920 | 7.82 | 1.61 | 1 | 2.59 | kinesin motor domain containing protein |  |
| 03g06950 | 5.97 | 2.02 | 0.01 | 2.58 | ubiquitin carboxyl-terminal hydrolase domain containing protein |  |
| 06g14460 | 10.71 | 1.01 | 2.59 | 2.57 | chromosome condensation protein like |  |
| 05g34700 | 31.91 | 1.21 | 9.56 | 2.56 | GDSL-like lipase/acylhydrolase |  |
| 03g04530 | 11.94 | 2.42 | 1.79 | 2.51 | cytochrome P450 |  |
| 01g09580 | 18.74 | 2.22 | 4.38 | 2.50 | serine/threonine-protein kinase Eg2 |  |
| 06g03080 | 6.59 | 1.01 | 1.39 | 2.46 | aspartic protease |  |
| 01g64640 | 39.32 | 4.24 | 10.35 | 2.43 | histone H3 |  |
| 03g62670 | 30.06 | 8.47 | 2.79 | 2.42 | expressed protein |  |
| 02g56540 | 7.41 | 1.41 | 1.39 | 2.40 | kinesin motor domain containing protein |  |
| 12g38140 | 17.5 | 1.41 | 5.18 | 2.40 | expressed protein |  |
| 05g08770 | 24.09 | 5.45 | 3.78 | 2.39 | uncharacterized protein At4g06744 precursor |  |
| 04g21350 | 48.59 | 12.1 | 6.57 | 2.38 | flowering promoting factor-like 1 |  |
| 12g35350 | 8.24 | 1.41 | 1.79 | 2.36 | kelch repeat protein |  |
| 01g64820 | 7.62 | 0.81 | 2.19 | 2.34 | POLA1 - DNA polymerase alpha catalytic subunit |  |
| 02g40430 | 5.56 | 2.22 | 0.01 | 2.34 | HEAT repeat family protein |  |
| 04g42540 | 5.97 | 0.4 | 1.99 | 2.31 | expressed protein |  |
| 03g52650 | 15.85 | 3.63 | 2.79 | 2.30 | syntaxin-related protein |  |
| 01g68930 | 6.18 | 0.81 | 1.79 | 2.25 | expressed protein |  |
| 05g05810 | 6.59 | 2.22 | 0.6 | 2.23 | DEAD-box ATP-dependent RNA helicase |  |
| 04g52630 | 7.41 | 2.02 | 1.19 | 2.21 | leucine-rich repeat-containing protein kinase protein |  |
| 06g40630 | 5.97 | 1.61 | 1 | 2.20 | SFT2 |  |
| 09g36790 | 6.38 | 1.21 | 1.59 | 2.19 | expressed protein |  |
| 02g52040 | 13.59 | 1.61 | 4.38 | 2.17 | phosphate-induced protein 1 domain containing protein |  |
| 01g60670 | 7.21 | 0.01 | 3.19 | 2.17 | receptor-like protein kinase precursor |  |
| 04g02680 | 14.82 | 6.66 | 0.01 | 2.16 | expressed protein |  |
| 03g47940 | 29.44 | 3.03 | 10.16 | 2.16 | GDSL-like lipase/acylhydrolase |  |
| 05g30500 | 57.85 | 14.52 | 11.55 | 2.15 | expressed protein |  |
| 03g10240 | 6.18 | 1.21 | 1.59 | 2.14 | DUF677 domain containing protein |  |
| 09g26900 | 6.59 | 2.22 | 0.8 | 2.14 | ctr copper transporter family protein |  |
| 08g38300 | 105.2 | 11.3 | 37.04 | 2.12 | Histone H2B |  |
| 01g72700 | 17.5 | 8.27 | 0.01 | 2.09 | expressed protein |  |
| 02g55410 | 6.38 | 1.21 | 1.79 | 2.09 | MCM5 - Minichromosome maintenance complex subunit 5 |  |
| 01g33080 | 8.44 | 1.41 | 2.59 | 2.08 | fimbrin-like protein 2 |  |
| 01g29230 | 8.44 | 2.02 | 1.99 | 2.08 | expressed protein |  |
| 12g36890 | 9.68 | 1.21 | 3.39 | 2.07 | CSLD4 - cellulose synthase-like family D |  |
| 04g58720 | 9.68 | 2.42 | 2.19 | 2.07 | anthranilate phosphoribosyltransferase |  |
| 12g39830 | 19.35 | 4.44 | 4.78 | 2.07 | cyclin delta-3 |  |
| **Table S2. (Continued)** | | | | | | |
| **Gene**  **(LOC_Os)** | **TPM**  **-SY** | **TPM**  **-ZS** | **TPM**  **-MH** | **log_2_**  **(SY/mid)** | **Description** |  |
| 02g01140 | 10.5 | 3.83 | 1.19 | 2.06 | GDSL-like lipase/acylhydrolase |  |
| 08g33660 | 24.71 | 7.87 | 3.98 | 2.06 | MYB family transcription factor |  |
| 01g60740 | 49 | 5.45 | 17.92 | 2.06 | Protease inhibitor/seed storage/LTP family protein precursor |  |
| 02g22020 | 11.74 | 2.22 | 3.39 | 2.06 | MYB family transcription factor |  |
| 03g46640 | 21.62 | 7.67 | 2.79 | 2.05 | deoxyuridine 5-triphosphate nucleotidohydrolase |  |
| 08g33100 | 58.47 | 15.33 | 12.94 | 2.05 | Histone H2A |  |
| 05g34820 | 8.24 | 2.02 | 1.99 | 2.04 | expressed protein |  |
| 02g07060 | 99.03 | 29.45 | 18.72 | 2.04 | tubulin/FtsZ domain containing protein |  |
| 02g10490 | 7.82 | 2.42 | 1.39 | 2.04 | expressed protein |  |
| 01g65440 | 16.88 | 5.04 | 3.19 | 2.04 | universal stress protein domain containing protein |  |
| 07g49140 | 28.41 | 6.46 | 7.57 | 2.02 | expressed protein |  |
| 01g11550 | 11.74 | 1.21 | 4.58 | 2.01 | TCP family transcription factor |  |
| 05g43140 | 9.26 | 1.41 | 3.19 | 2.01 | expressed protein |  |
| 02g58220 | 10.09 | 2.02 | 2.99 | 2.01 | RPA2A - Single-stranded DNA binding complex subunit 2 |  |
| 06g51110 | 19.35 | 2.82 | 6.97 | 1.98 | G2/mitotic specific cyclin 2 |  |
| 03g40830 | 8.24 | 2.82 | 1.39 | 1.97 | OsSub30 - Putative Subtilisin homologue |  |
| 12g36640 | 7 | 2.02 | 1.59 | 1.96 | universal stress protein domain containing protein |  |
| 06g14810 | 32.12 | 10.69 | 6.37 | 1.91 | 3-ketoacyl-CoA synthase |  |
| 05g33890 | 13.18 | 0.81 | 6.17 | 1.91 | microtubule associated protein |  |
| 01g68650 | 8.44 | 2.82 | 1.79 | 1.88 | plant-specific domain TIGR01615 family protein |  |
| 01g14850 | 322.6 | 56.28 | 119.9 | 1.87 | MFS18 protein precursor |  |
| 02g44940 | 9.88 | 3.43 | 1.99 | 1.87 | RALFL8-Rapid ALkalinization Factor protein precursor |  |
| 11g05290 | 19.15 | 5.85 | 4.78 | 1.85 | stress responsive A/B Barrel domain containing protein |  |
| 11g13750 | 7.21 | 1.61 | 2.39 | 1.85 | expressed protein |  |
| 03g14500 | 10.09 | 2.62 | 2.99 | 1.84 | expressed protein |  |
| 06g43170 | 22.65 | 7.46 | 5.18 | 1.84 | transposon protein |  |
| 05g38000 | 122.7 | 49.22 | 20.11 | 1.83 | ATROPGEF7/ROPGEF7 |  |
| 01g06580 | 52.91 | 15.94 | 13.94 | 1.83 | fasciclin domain containing protein |  |
| 08g39550 | 26.97 | 8.27 | 6.97 | 1.82 | polygalacturonase inhibitor 2 precursor |  |
| 11g37950 | 25.12 | 8.67 | 5.58 | 1.82 | WIP3 - Wound-induced protein precursor |  |
| 02g22820 | 11.32 | 4.44 | 1.99 | 1.82 | expressed protein |  |
| 06g03682 | 9.88 | 1.61 | 3.98 | 1.81 | calcium-dependent protein kinase isoform AK1 |  |
| 04g55570 | 8.03 | 1.41 | 3.19 | 1.80 | DUF869 domain containing protein |  |
| 01g65986 | 10.09 | 3.83 | 1.99 | 1.79 | DUF803 domain containing protein |  |
| 07g10600 | 166.6 | 75.65 | 21.31 | 1.79 | cycloartenol-C-24-methyltransferase 1 |  |
| 01g12880 | 7.21 | 2.22 | 1.99 | 1.78 | VHS and GAT domain containing protein |  |
| 03g13130 | 21.62 | 8.07 | 4.58 | 1.78 | ternary complex factor MIP1 |  |
| 02g13660 | 45.91 | 7.67 | 19.12 | 1.77 | meiosis 5 |  |
| 02g49720 | 68.97 | 17.15 | 23.5 | 1.76 | aldehyde dehydrogenase |  |
| 03g44290 | 12.56 | 2.82 | 4.58 | 1.76 | expansin precursor |  |
| 01g46510 | 40.35 | 12.3 | 11.55 | 1.76 | peptidase S8 and S53, subtilisin, kexin, sedolisin |  |
| **Table S2. (Continued)** | | | | | | |
| **Gene**  **(LOC_Os)** | **TPM**  **-SY** | **TPM**  **-ZS** | **TPM**  **-MH** | **log_2_**  **(SY/mid)** | **Description** |  |
| 07g40910 | 10.5 | 2.42 | 3.78 | 1.76 | expressed protein |  |
| 01g13530 | 9.47 | 3.83 | 1.79 | 1.75 | ABIL2 |  |
| 05g37160 | 18.12 | 7.26 | 3.58 | 1.74 | tubulin/FtsZ domain containing protein |  |
| 04g39110 | 29.85 | 5.24 | 12.54 | 1.74 | GASR4 - Gibberellin-regulated family protein precursor |  |
| 11g08569 | 8.03 | 2.02 | 2.79 | 1.74 | cycloartenol synthase |  |
| 06g46995 | 9.68 | 3.63 | 2.19 | 1.73 | armadillo/beta-catenin repeat family protein |  |
| 12g29580 | 12.35 | 4.84 | 2.79 | 1.70 | protein kinase PVRK-1 |  |
| 04g39410 | 8.44 | 2.82 | 2.39 | 1.69 | pentatricopeptide |  |
| 01g67870 | 22.65 | 7.26 | 6.77 | 1.69 | expressed protein |  |
| 12g34350 | 24.29 | 8.88 | 6.17 | 1.69 | expressed protein |  |
| 11g32100 | 17.71 | 5.45 | 5.58 | 1.68 | inducer of CBF expression 1 |  |
| 09g24900 | 16.47 | 6.05 | 4.58 | 1.63 | methyltransferase |  |
| 10g39740 | 52.5 | 17.95 | 16.33 | 1.61 | glutathione S-transferase |  |
| 01g11350 | 8.85 | 2.62 | 3.19 | 1.60 | bZIP transcription factor domain containing protein |  |
| 01g51300 | 35.41 | 16.74 | 6.77 | 1.60 | WD domain, G-beta repeat domain containing protein |  |
| 04g52320 | 12.97 | 5.04 | 3.58 | 1.59 | QRT3 |  |
| 01g61350 | 11.12 | 4.24 | 3.19 | 1.58 | glutaredoxin |  |
| 05g49780 | 10.5 | 3.43 | 3.58 | 1.58 | histone-like transcription factor and archaeal histone |  |
| 01g72890 | 70.21 | 26.02 | 21.11 | 1.58 | transposon protein, CACTA, En/Spm sub-class |  |
| 10g25780 | 111.4 | 39.74 | 35.05 | 1.58 | FAD-linked oxidoreductase protein |  |
| 03g05520 | 80.71 | 33.28 | 21.31 | 1.57 | nicotiana lesion-inducing like |  |
| 02g50820 | 16.26 | 5.85 | 5.18 | 1.56 | nucleobase-ascorbate transporter |  |
| 03g59480 | 11.53 | 5.45 | 2.39 | 1.56 | expressed protein |  |
| 02g48730 | 9.06 | 3.03 | 3.19 | 1.54 | rho GDP-dissociation inhibitor 1 |  |
| 07g19530 | 19.35 | 8.67 | 4.78 | 1.53 | peptidase |  |
| 10g42230 | 10.09 | 2.62 | 4.38 | 1.52 | AT hook motif domain containing protein |  |
| 04g34170 | 42.62 | 17.55 | 12.35 | 1.51 | retrotransposon protein |  |
| 07g31840 | 14 | 4.44 | 5.38 | 1.51 | leucine-rich repeat family protein |  |
| 01g69120 | 10.29 | 4.44 | 2.79 | 1.51 | uncharacterized RNA methyltransferase pc1998 |  |
| 07g02200 | 21.41 | 5.24 | 9.76 | 1.51 | plastocyanin-like domain containing protein |  |
| 02g40784 | 11.94 | 2.82 | 5.58 | 1.50 | WAX2 |  |
| 09g27830 | 41.18 | 13.31 | 15.73 | 1.50 | OsPDIL2-3 protein disulfide isomerase |  |
| 02g41800 | 14.21 | 5.45 | 4.78 | 1.48 | auxin response factor |  |
| 05g42330 | 28.41 | 11.9 | 8.56 | 1.48 | secretory carrier-associated membrane protein |  |
| 03g49990 | 23.68 | 10.49 | 6.57 | 1.47 | GRAS domain containing protein |  |
| 05g11810 | 78.24 | 37.32 | 19.31 | 1.47 | gibberellin 2-beta-dioxygenase 1 |  |
| 06g06490 | 39.12 | 12.3 | 16.13 | 1.46 | U-box domain containing heat shock protein |  |
| 05g20050 | 34.38 | 16.34 | 8.76 | 1.46 | ras-related protein |  |
| 04g40850 | 29.65 | 14.12 | 7.57 | 1.45 | 26S proteasome non-ATPase regulatory subunit 6 |  |
| 06g41110 | 16.06 | 7.67 | 4.18 | 1.44 | tubulin binding cofactor C |  |
| 03g14900 | 22.85 | 8.67 | 8.16 | 1.44 | NB-ARC/LRR disease resistance protein |  |
| **Table S2. (Continued)** | | | | | | |
| **Gene**  **(LOC_Os)** | **TPM**  **-SY** | **TPM**  **-ZS** | **TPM**  **-MH** | **log_2_**  **(SY/mid)** | **Description** |  |
| 08g17160 | 15.44 | 5.04 | 6.37 | 1.44 | plastocyanin-like domain containing protein |  |
| 04g56720 | 46.53 | 22.79 | 12.15 | 1.42 | regulator of chromosome condensation family protein |  |
| 04g58504 | 10.91 | 5.45 | 2.79 | 1.41 | choline transporter-related |  |
| 03g06510 | 25.53 | 8.67 | 10.55 | 1.41 | KIP1 |  |
| 07g03120 | 12.76 | 5.85 | 3.78 | 1.41 | expressed protein |  |
| 01g71200 | 31.71 | 14.52 | 9.56 | 1.40 | RNA recognition motif containing protein |  |
| 03g41060 | 17.91 | 6.86 | 6.77 | 1.40 | GASR2-Gibberellin-regulated family protein precursor |  |
| 02g01332 | 63.41 | 23 | 25.29 | 1.39 | ribosomal protein L6 |  |
| 08g10510 | 37.06 | 16.94 | 11.35 | 1.39 | aminotransferase |  |
| 03g02750 | 17.5 | 8.27 | 5.18 | 1.38 | OsSub25 - Subtilisin homologue |  |
| 01g68598 | 12.97 | 3.83 | 6.17 | 1.37 | expressed protein |  |
| 03g49430 | 17.09 | 6.25 | 6.97 | 1.37 | pre-mRNA-splicing factor |  |
| 04g46740 | 55.18 | 21.18 | 21.7 | 1.36 | pectinesterase |  |
| 03g15560 | 20.59 | 9.68 | 6.37 | 1.36 | wibg |  |
| 08g35090 | 11.74 | 4.44 | 4.78 | 1.35 | OsSub56 - Subtilisin homologue |  |
| 02g08220 | 15.24 | 6.46 | 5.58 | 1.34 | expressed protein |  |
| 06g48160 | 88.32 | 40.55 | 29.27 | 1.34 | glycosyl hydrolases family 16 |  |
| 05g48810 | 12.15 | 5.04 | 4.58 | 1.34 | dnaJ domain containing protein |  |
| 02g47180 | 13.38 | 5.85 | 4.78 | 1.33 | WD repeat-containing protein |  |
| 01g70100 | 13.38 | 4.84 | 5.77 | 1.33 | zinc finger DHHC domain-containing protein |  |
| 09g31970 | 85.24 | 38.13 | 29.67 | 1.33 | 3-hydroxy-3-methylglutaryl-coenzyme A reductase |  |
| 09g39410 | 66.91 | 23.4 | 29.87 | 1.33 | male sterility protein |  |
| 03g43860 | 27.59 | 11.09 | 10.95 | 1.32 | SNF7 domain containing protein |  |
| 04g48140 | 11.53 | 4.64 | 4.58 | 1.32 | methyltransferase |  |
| 04g43670 | 23.68 | 7.46 | 11.55 | 1.31 | expressed protein |  |
| 02g54060 | 35.82 | 17.55 | 11.35 | 1.31 | chaperonin |  |
| 02g56130 | 22.85 | 8.88 | 9.56 | 1.31 | PCNA - Putative DNA replicative polymerase clamp |  |
| 03g48600 | 24.5 | 11.3 | 8.56 | 1.30 | DUF966 domain containing protein |  |
| 01g18050 | 123.74 | 43.37 | 57.15 | 1.30 | tubulin/FtsZ domain containing protein |  |
| 08g43440 | 47.97 | 22.19 | 16.93 | 1.30 | cytochrome P450 |  |
| 02g10690 | 76.38 | 31.67 | 30.66 | 1.29 | targeting protein for Xklp2 |  |
| 05g15510 | 21.82 | 10.09 | 7.77 | 1.29 | cellulase |  |
| 05g51470 | 19.77 | 9.48 | 6.77 | 1.28 | 2-aminoethanethiol dioxygenase |  |
| 02g08300 | 13.59 | 5.85 | 5.38 | 1.28 | RAD23 DNA repair protein |  |
| 08g39270 | 25.94 | 10.69 | 10.75 | 1.27 | fasciclin domain containing protein |  |
| 08g12430 | 15.24 | 5.65 | 6.97 | 1.27 | VERNALIZATION-INSENSITIVE |  |
| 06g40150 | 39.94 | 16.94 | 16.93 | 1.24 | AP2 domain containing protein, expressed |  |
| 07g37350 | 23.27 | 10.49 | 9.36 | 1.23 | armadillo/beta-catenin repeat family protein |  |
| 02g25060 | 120.24 | 50.63 | 52.77 | 1.22 | NTMC2Type1.2 protein |  |
| 11g36940 | 21.41 | 8.47 | 10.16 | 1.20 | glucan endo-1,3-beta-glucosidase precursor |  |
| 04g44650 | 24.5 | 11.7 | 9.76 | 1.19 | ferredoxin-thioredoxin reductase, variable chain |  |
| **Table S2. (Continued)** | | | | | | |
| **Gene**  **(LOC_Os)** | **TPM**  **-SY** | **TPM**  **-ZS** | **TPM**  **-MH** | **log_2_**  **(SY/mid)** | **Description** |  |
| 08g16830 | 49.41 | 22.19 | 21.11 | 1.19 | retrotransposon protein, SINE subclass |  |
| 01g44110 | 25.94 | 11.5 | 11.35 | 1.18 | serine/threonine-protein kinase |  |
| 03g44420 | 26.35 | 12.71 | 10.75 | 1.17 | tubulin/FtsZ domain containing protein |  |
| 07g40550 | 51.68 | 24.21 | 21.9 | 1.16 | IBS1 |  |
| 05g44400 | 39.32 | 17.35 | 17.92 | 1.16 | GATA zinc finger domain containing protein |  |
| 05g02530 | 17.29 | 8.07 | 7.57 | 1.14 | glutathione S-transferase |  |
| 11g07680 | 17.71 | 8.07 | 7.96 | 1.14 | dirigent |  |
| 05g39050 | 23.47 | 11.09 | 10.35 | 1.13 | Histone H4 |  |
| 04g45190 | 17.29 | 8.07 | 7.77 | 1.13 | MSP domain containing protein |  |
| 06g05950 | 68.35 | 33.28 | 29.87 | 1.11 | expressed protein |  |
| 01g03070 | 30.88 | 14.93 | 14.34 | 1.08 | transposon protein |  |
| 01g42294 | 71.24 | 33.49 | 34.45 | 1.07 | inactive receptor kinase At2g26730 precursor |  |
| 03g21080 | 57.24 | 28.44 | 27.88 | 1.02 | guanine nucleotide exchange factor |  |
| 04g27060 | 0.01 | 2.02 | 11.15 | -9.37 | oxidoreductase, aldo/keto reductase family protein |  |
| 11g37700 | 0.01 | 0.4 | 12.54 | -9.35 | pleiotropic drug resistance protein |  |
| 09g27820 | 0.01 | 0.4 | 11.15 | -9.18 | 1-aminocyclopropane-1-carboxylate oxidase protein |  |
| 07g32710 | 0.01 | 0.4 | 9.56 | -8.97 | retrotransposon protein |  |
| 04g28234 | 0.01 | 0.81 | 7.57 | -8.72 | Rf1, mitochondrial precursor |  |
| 10g40360 | 0.01 | 2.42 | 5.38 | -8.61 | proline oxidase, mitochondrial precursor |  |
| 09g29310 | 0.01 | 2.22 | 5.58 | -8.61 | zinc finger, C3HC4 type domain containing protein |  |
| 03g08880 | 0.01 | 1.01 | 6.57 | -8.57 | purine permease |  |
| 05g24780 | 0.01 | 0.81 | 6.77 | -8.57 | OsCML21 - Calmodulin-related calcium sensor protein |  |
| 01g55940 | 0.01 | 0.81 | 6.17 | -8.46 | OsGH3.2 - Probable indole-3-acetic acid-amido synthetase |  |
| 07g05365 | 0.01 | 1.82 | 4.98 | -8.41 | photosystem II 10 kDa polypeptide, chloroplast precursor |  |
| 11g47630 | 0.01 | 0.61 | 6.17 | -8.41 | ZOS11-10 - C2H2 zinc finger protein |  |
| 06g41100 | 0.01 | 3.03 | 3.58 | -8.37 | transcription factor |  |
| 10g21310 | 0.01 | 1.21 | 5.18 | -8.33 | photosystem II P680 chlorophyll A apoprotein |  |
| 08g26350 | 0.01 | 0.4 | 5.97 | -8.33 | expressed protein |  |
| 04g32740 | 0.01 | 0.4 | 5.58 | -8.23 | hydrolase, NUDIX family, domain containing protein |  |
| 12g25660 | 0.01 | 2.62 | 2.99 | -8.13 | cytochrome P450 |  |
| 07g23430 | 0.41 | 1.01 | 28.47 | -5.18 | fatty acid desaturase |  |
| 10g11500 | 0.41 | 1.82 | 9.96 | -3.85 | SCP-like extracellular protein |  |
| 01g03130 | 0.41 | 3.83 | 7.37 | -3.77 | expressed protein |  |
| 09g39770 | 0.62 | 7.06 | 6.57 | -3.46 | C2 domain containing protein |  |
| 01g70490 | 0.62 | 2.82 | 10.55 | -3.44 | potassium transporter |  |
| 01g16170 | 1.44 | 4.44 | 24.49 | -3.33 | PQ loop repeat domain containing protein |  |
| 10g31330 | 2.88 | 13.72 | 39.23 | -3.20 | retrotransposon protein |  |
| 05g30350 | 1.44 | 4.03 | 20.51 | -3.10 | Os5bglu22 - beta-glucosidase homologue |  |
| 01g52790 | 1.03 | 3.83 | 11.75 | -2.92 | cytochrome P450 72A1 |  |
| 06g30370 | 1.24 | 5.45 | 12.74 | -2.88 | osMFT1 homologous to Mother of FT and TFL1 gene |  |
| 06g07030 | 3.5 | 28.64 | 15.53 | -2.66 | AP2 domain containing protein |  |
| **Table S2. (Continued)** | | | | | | |
| **Gene**  **(LOC_Os)** | **TPM**  **-SY** | **TPM**  **-ZS** | **TPM**  **-MH** | **log_2_**  **(SY/mid)** | **Description** |  |
| 03g12500 | 2.88 | 7.87 | 26.88 | -2.60 | cytochrome P450 |  |
| 05g34854 | 1.24 | 3.03 | 11.35 | -2.54 | gibberellin 20 oxidase 2 |  |
| 05g43390 | 1.03 | 6.86 | 4.78 | -2.50 | signal recognition particle 54 kDa protein |  |
| 02g53130 | 1.03 | 6.46 | 4.78 | -2.45 | nitrate reductase |  |
| 03g10320 | 1.65 | 3.83 | 13.14 | -2.37 | expressed protein |  |
| 08g35630 | 1.65 | 4.64 | 11.75 | -2.32 | MTD1 |  |
| 01g38610 | 5.15 | 18.36 | 30.47 | -2.25 | helix-loop-helix DNA-binding domain containing protein |  |
| 02g35490 | 4.32 | 10.49 | 30.47 | -2.25 | MLO domain containing protein |  |
| 05g05390 | 3.09 | 7.67 | 21.11 | -2.22 | expressed protein |  |
| 01g67770 | 1.44 | 4.84 | 8.36 | -2.20 | two-component response regulator |  |
| 09g18360 | 3.09 | 7.06 | 21.31 | -2.20 | expressed protein |  |
| 01g62490 | 2.26 | 4.84 | 15.93 | -2.20 | laccase precursor protein |  |
| 05g34830 | 3.71 | 12.71 | 19.91 | -2.14 | No apical meristem protein |  |
| 06g09310 | 2.47 | 6.25 | 15.33 | -2.13 | zinc finger, C3HC4 type domain containing protein |  |
| 03g45770 | 11.12 | 36.11 | 59.94 | -2.11 | expressed protein |  |
| 06g07070 | 3.09 | 15.73 | 10.55 | -2.09 | BR1-associated receptor kinase 1 precursor |  |
| 05g27340 | 2.47 | 9.28 | 11.75 | -2.09 | expressed protein |  |
| 03g20600 | 1.65 | 5.24 | 8.56 | -2.07 | expressed protein |  |
| 09g01290 | 1.85 | 6.05 | 9.36 | -2.06 | hhH-GPD superfamily base excision DNA repair protein |  |
| 08g17060 | 1.65 | 6.05 | 7.57 | -2.05 | expressed protein |  |
| 09g33530 | 1.85 | 5.04 | 10.16 | -2.04 | expressed protein |  |
| 08g10430 | 2.06 | 7.67 | 8.96 | -2.01 | NBS-LRR disease resistance protein |  |
| 04g27190 | 2.06 | 5.24 | 11.15 | -2.00 | terpene synthase |  |
| 04g31790 | 1.85 | 4.03 | 10.75 | -2.00 | expressed protein |  |
| 07g42900 | 4.74 | 17.55 | 19.51 | -1.97 | helicase domain-containing protein |  |
| 06g40110 | 1.85 | 5.04 | 9.36 | -1.96 | RNA polymerase Rpc34 subunit family protein |  |
| 09g23570 | 2.06 | 7.26 | 8.56 | -1.94 | inactive receptor kinase At2g26730 precursor |  |
| 02g54860 | 2.88 | 7.26 | 14.34 | -1.91 | ankyrin repeat-rich protein |  |
| 01g70730 | 2.06 | 4.24 | 10.95 | -1.89 | flowering promoting factor-like 1 |  |
| 05g12400 | 8.24 | 25.01 | 35.44 | -1.88 | BURP domain containing protein |  |
| 12g40180 | 20.79 | 47.81 | 102.75 | -1.86 | expressed protein |  |
| 08g37670 | 48.79 | 103.5 | 250.3 | -1.86 | plastocyanin-like domain containing protein |  |
| 11g06320 | 2.26 | 4.84 | 11.35 | -1.85 | expressed protein |  |
| 09g19970 | 3.09 | 9.48 | 12.54 | -1.83 | expressed protein |  |
| 03g43510 | 3.71 | 10.69 | 14.73 | -1.78 | expressed protein |  |
| 10g12050 | 2.47 | 7.67 | 8.96 | -1.75 | expressed protein |  |
| 12g43640 | 4.74 | 11.09 | 20.11 | -1.72 | receptor-like protein kinase HAIKU2 precursor |  |
| 08g02180 | 7.62 | 16.14 | 34.05 | -1.72 | expressed protein |  |
| 05g05600 | 9.06 | 22.79 | 36.24 | -1.71 | ATA15 protein |  |
| 10g30610 | 7.82 | 17.15 | 34.05 | -1.71 | white-brown complex homolog protein |  |
| 03g58980 | 3.91 | 10.09 | 15.53 | -1.71 | Cupin domain containing protein |  |
| **Table S2. (Continued)** | | | | | | |
| **Gene**  **(LOC_Os)** | **TPM**  **-SY** | **TPM**  **-ZS** | **TPM**  **-MH** | **log_2_**  **(SY/mid)** | **Description** |  |
| 10g34170 | 6.59 | 16.14 | 25.49 | -1.66 | glutaredoxin domain containing protein |  |
| 04g40630 | 2.88 | 9.48 | 8.56 | -1.65 | TAZ zinc finger family protein |  |
| 04g51150 | 4.12 | 11.7 | 14.14 | -1.65 | transposon protein |  |
| 12g36910 | 16.26 | 35.7 | 64.52 | -1.63 | calmodulin binding protein |  |
| 04g38720 | 5.15 | 12.3 | 19.51 | -1.63 | no apical meristem protein |  |
| 06g10210 | 4.12 | 9.68 | 15.53 | -1.62 | expressed protein |  |
| 01g49820 | 3.5 | 7.26 | 13.74 | -1.59 | lipid phosphatase protein |  |
| 08g34790 | 8.85 | 20.98 | 31.66 | -1.57 | AMP-binding domain containing protein |  |
| 07g38830 | 4.74 | 14.52 | 13.34 | -1.56 | hydrolase, alpha/beta fold family protein |  |
| 01g04280 | 5.35 | 11.3 | 20.11 | -1.56 | calmodulin binding protein |  |
| 06g51360 | 6.38 | 13.31 | 23.7 | -1.54 | lysM domain containing protein |  |
| 01g67190 | 3.71 | 7.67 | 13.94 | -1.54 | ribonuclease T2 family domain containing protein |  |
| 03g04770 | 8.44 | 25.82 | 22.3 | -1.51 | beta-amylase |  |
| 05g06890 | 3.71 | 8.47 | 12.54 | -1.50 | relA-SpoT like protein RSH4 |  |
| 06g06080 | 5.97 | 14.12 | 19.71 | -1.50 | serine esterase family protein |  |
| 05g48940 | 4.74 | 11.3 | 15.33 | -1.49 | expressed protein |  |
| 03g50380 | 4.32 | 9.08 | 14.93 | -1.48 | DNA-directed RNA polymerase I subunit RPA12 |  |
| 01g42410 | 13.38 | 27.64 | 45.6 | -1.45 | pleiotropic drug resistance protein |  |
| 03g61490 | 9.68 | 20.37 | 31.66 | -1.43 | expressed protein |  |
| 01g09570 | 13.18 | 27.23 | 43.81 | -1.43 | 6-phosphofructokinase |  |
| 12g07550 | 8.24 | 22.19 | 21.51 | -1.41 | expressed protein |  |
| 02g06570 | 4.74 | 10.49 | 14.14 | -1.38 | CBL-interacting serine/threonine-protein kinase 15 |  |
| 06g08140 | 5.15 | 11.9 | 14.93 | -1.38 | protein phosphatase 2C |  |
| 10g29470 | 5.35 | 11.3 | 16.33 | -1.37 | dehydrogenase |  |
| 03g13274 | 11.32 | 23.2 | 34.65 | -1.36 | peptide transporter PTR2 |  |
| 02g43700 | 6.79 | 15.33 | 19.31 | -1.35 | triacylglycerol lipase like protein |  |
| 01g65890 | 7.41 | 20.58 | 17.32 | -1.35 | DNA repair metallo-beta-lactamase |  |
| 03g50210 | 10.71 | 23.2 | 30.86 | -1.34 | DUF292 domain containing protein |  |
| 06g44140 | 7.62 | 18.15 | 20.51 | -1.34 | transmembrane 9 superfamily member |  |
| 11g10470 | 10.09 | 28.64 | 22.3 | -1.33 | expressed protein |  |
| 01g67810 | 16.06 | 45.19 | 34.85 | -1.32 | transposon protein |  |
| 05g48930 | 36.85 | 85.53 | 94.18 | -1.29 | OsGrx_S2 - glutaredoxin subgroup III |  |
| 03g22620 | 9.06 | 21.38 | 22.9 | -1.29 | terpene synthase family, metal binding domain containing |  |
| 03g08330 | 30.27 | 64.15 | 83.83 | -1.29 | ZIM domain containing protein |  |
| 04g48290 | 5.15 | 12.71 | 12.54 | -1.29 | MATE efflux family protein |  |
| 02g30230 | 5.15 | 10.49 | 14.54 | -1.28 | exo70 exocyst complex subunit domain containing protein |  |
| 02g04750 | 4.94 | 10.89 | 13.14 | -1.28 | cycloartenol synthase |  |
| 02g39730 | 12.97 | 35.5 | 26.68 | -1.26 | expressed protein |  |
| 05g41610 | 10.71 | 28.24 | 23.1 | -1.26 | glycosyl hydrolases family 17 |  |
| 09g31130 | 11.32 | 27.23 | 26.48 | -1.25 | citrate transporter |  |
| 02g14480 | 5.76 | 15.13 | 12.15 | -1.24 | receptor-like kinase |  |
| **Table S2. (Continued)** | | | | | | |
| **Gene**  **(LOC_Os)** | **TPM**  **-SY** | **TPM**  **-ZS** | **TPM**  **-MH** | **log_2_**  **(SY/mid)** | **Description** |  |
| 03g10140 | 11.12 | 23.4 | 29.27 | -1.24 | ZOS3-04 - C2H2 zinc finger protein |  |
| 11g08100 | 12.97 | 27.03 | 33.65 | -1.23 | eukaryotic aspartyl protease domain containing protein |  |
| 06g49700 | 5.56 | 11.5 | 14.54 | -1.23 | expressed protein |  |
| 02g54600 | 35.62 | 74.03 | 89.8 | -1.20 | OsMKK4-MAPKK protein |  |
| 10g39430 | 9.26 | 23 | 19.31 | -1.19 | expressed protein |  |
| 01g17050 | 5.97 | 14.73 | 12.54 | -1.19 | VQ domain containing protein |  |
| 11g04390 | 8.85 | 18.56 | 21.51 | -1.18 | RNA recognition motif containing protein |  |
| 01g55510 | 23.47 | 58.1 | 48.39 | -1.18 | dynein light chain type 1 domain containing protein |  |
| 07g44160 | 20.79 | 45.19 | 47.39 | -1.16 | retrotransposon protein |  |
| 01g70240 | 7.62 | 18.56 | 15.53 | -1.16 | expressed protein |  |
| 10g11354 | 7.41 | 16.94 | 15.93 | -1.15 | MATE efflux family protein |  |
| 01g70010 | 16.68 | 36.51 | 36.84 | -1.14 | ribosomal protein L7Ae |  |
| 02g47470 | 42.21 | 97.63 | 88.61 | -1.14 | cytochrome P450 |  |
| 05g46040 | 9.26 | 19.37 | 21.11 | -1.13 | protein phosphatase 2C |  |
| 05g31620 | 7.41 | 16.74 | 15.33 | -1.11 | OsCML15 - Calmodulin-related calcium sensor protein |  |
| 05g41780 | 100.06 | 213.02 | 211.67 | -1.09 | AP2 domain containing protein |  |
| 03g21380 | 12.97 | 27.23 | 27.08 | -1.07 | OsCML27 - Calmodulin-related calcium sensor protein |  |
| 07g12340 | 9.47 | 20.37 | 19.31 | -1.07 | NAC domain-containing protein 67 |  |
| 04g58850 | 35.62 | 71.81 | 71.68 | -1.01 | harpin-induced protein 1 domain containing protein |  |

TPM (Transcripts Per Million clean tags) is a standardized indicator, pointing out number of transcript copies in every 1 million clean tags.Mid means the average TPM value of MH and ZS
