# Supplementary material for: Altered Levels of Histone Deacetylase OsHDT1 Affect Differential Gene Expression Patterns in Hybrid Rice
Source: PLoS One. 2011 Jul 8;6(7):e21789. doi: 10.1371/journal.pone.0021789 (PMC3132746; doi:10.1371/journal.pone.0021789)
Supplement: Table S3 — Differential expressed genes between transgenic and wild type hybrids. (DOCX) [file pone.0021789.s007.docx]

**Table S3.** Differential expressed genes between transgenetic (FU and FR) and wild type SY63 hybrid lines

| **Gene** | **TPM-SY** | **TPM-FU** | **log_2_(FU/SY)** | **Description** |
| --- | --- | --- | --- | --- |
| 07g24830 | 0.62 | 45.84 | 6.21 | thionin-like peptide, putative, expressed |
| 12g27254 | 1.65 | 34.22 | 4.37 | transferase family protein, putative, expressed |
| 05g51830 | 88.12 | 1574.2 | 4.16 | ZOS5-12 - C2H2 zinc finger protein, expressed |
| 10g42040 | 1.03 | 13.07 | 3.67 | expressed protein |
| 05g09410 | 0.62 | 7.67 | 3.63 | histidine-containing phosphotransfer protein 4, putative, expressed |
| 03g43100 | 1.85 | 22.4 | 3.60 | expressed protein |
| 07g03730 | 8.65 | 99.14 | 3.52 | SCP-like extracellular protein, expressed |
| 11g31470 | 1.24 | 14.1 | 3.51 | expressed protein |
| 07g19000 | 0.62 | 6.84 | 3.46 | LTPL41 - Protease inhibitor/seed storage/LTP family protein precursor |
| 10g37160 | 1.65 | 16.18 | 3.29 | transposon protein, putative, unclassified, expressed |
| 08g37210 | 0.62 | 6.01 | 3.28 | patatin, putative, expressed |
| 05g30510 | 0.62 | 5.81 | 3.23 | expressed protein |
| 06g11210 | 1.44 | 13.48 | 3.23 | 12-oxophytodienoate reductase, putative, expressed |
| 07g34520 | 1.65 | 15.14 | 3.20 | isocitrate lyase, putative, expressed |
| 01g03320 | 53.53 | 466.85 | 3.12 | BBTI2 - Bowman-Birk type bran trypsin inhibitor precursor, expressed |
| 01g50940 | 1.65 | 14.1 | 3.10 | helix-loop-helix DNA-binding domain containing protein, expressed |
| 10g35950 | 0.62 | 5.18 | 3.06 | transferase family protein, putative, expressed |
| 01g07850 | 0.82 | 6.84 | 3.06 | glyoxalase family protein, putative, expressed |
| 12g37260 | 58.27 | 474.74 | 3.03 | lipoxygenase 2.1, chloroplast precursor, putative, expressed |
| 09g29690 | 1.65 | 13.27 | 3.01 | beta-expansin precursor, putative, expressed |
| 01g37000 | 1.24 | 9.54 | 2.94 | carboxyl-terminal peptidase, putative, expressed |
| 06g20960 | 0.82 | 6.22 | 2.92 | SAM dependent carboxyl methyltransferase, putative |
| 02g29210 | 1.65 | 12.44 | 2.91 | ankyrin, putative, expressed |
| 01g56810 | 1.85 | 13.69 | 2.89 | cytokinin dehydrogenase precursor, putative, expressed |
| 03g50960 | 0.82 | 6.01 | 2.87 | LTPL118-Protease inhibitor/seed storage/LTP family protein precursor |
| 03g20600 | 1.65 | 11.61 | 2.81 | expressed protein |
| 09g01000 | 145.97 | 1024.1 | 2.81 | expressed protein |
| 12g27220 | 3.71 | 25.92 | 2.80 | transferase family protein, putative, expressed |
| 01g16170 | 1.44 | 9.96 | 2.79 | PQ loop repeat domain containing protein, expressed |
| 06g24990 | 11.74 | 79.43 | 2.76 | xylanase inhibitor protein 1 precursor, putative, expressed |
| 04g52440 | 1.24 | 8.3 | 2.74 | aminotransferase, putative, expressed |
| 07g13810 | 0.82 | 5.39 | 2.72 | cytokinin-N-glucosyltransferase 1, putative, expressed |
| 03g52860 | 28 | 176.91 | 2.66 | lipoxygenase, putative, expressed |
| 03g10320 | 1.65 | 10.37 | 2.65 | expressed protein |
| 12g27350 | 19.56 | 121.12 | 2.63 | 10-deacetylbaccatin III 10-O-acetyltransferase, putative |
| 10g24004 | 3.71 | 22.61 | 2.61 | hypothetical protein |
| 01g38610 | 5.15 | 31.32 | 2.60 | helix-loop-helix DNA-binding domain containing protein, expressed |
| 04g44110 | 1.03 | 6.22 | 2.59 | hydrolase, putative, expressed |
| 12g09700 | 8.03 | 48.12 | 2.58 | Jacalin-like lectin domain containing protein, putative, expressed |
| 02g43970 | 1.44 | 8.5 | 2.56 | AP2 domain containing protein, expressed |
| **Table S3. (Continued)** | | | | |
| **Gene** | **TPM-SY** | **TPM-FU** | **log_2_(FU/SY)** | **Description** |
| 12g01350 | 1.03 | 6.01 | 2.54 | csAtPR5, putative, expressed |
| 09g23530 | 1.65 | 9.54 | 2.53 | dehydrogenase, putative, expressed |
| 01g53090 | 1.44 | 8.3 | 2.53 | pathogen-related protein, putative, expressed |
| 04g58890 | 1.85 | 10.58 | 2.52 | expressed protein |
| 05g06320 | 1.03 | 5.81 | 2.50 | ethylene receptor, putative, expressed |
| 04g43200 | 3.5 | 19.08 | 2.45 | caleosin related protein, putative, expressed |
| 03g18130 | 12.35 | 66.78 | 2.43 | asparagine synthetase, putative, expressed |
| 02g54860 | 2.88 | 15.35 | 2.41 | ankyrin repeat-rich protein, putative, expressed |
| 08g25060 | 1.44 | 7.67 | 2.41 | BSD domain-containing protein, putative, expressed |
| 03g08900 | 1.44 | 7.47 | 2.38 | MATE efflux family protein, putative, expressed |
| 05g33820 | 1.24 | 6.43 | 2.37 | lipase, putative, expressed |
| 04g34250 | 1.24 | 6.22 | 2.33 | serine/threonine-protein kinase receptor precursor, putative, expressed |
| 01g03330 | 6.38 | 30.9 | 2.28 | BBTI3 - Bowman-Birk type bran trypsin inhibitor precursor, expressed |
| 01g10340 | 2.88 | 13.9 | 2.27 | expressed protein |
| 01g24710 | 28.21 | 134.39 | 2.25 | jacalin-like lectin domain containing protein, expressed |
| 12g36735 | 1.44 | 6.84 | 2.25 | expressed protein |
| 03g29340 | 1.85 | 8.71 | 2.24 | domain of unknown function domain containing protein, expressed |
| 02g02780 | 2.47 | 11.61 | 2.23 | protein kinase family protein, putative, expressed |
| 05g47750 | 2.47 | 11.61 | 2.23 | Ser/Thr protein kinase, putative, expressed |
| 06g37150 | 15.03 | 70.1 | 2.22 | L-ascorbate oxidase precursor, putative, expressed |
| 02g41670 | 2.47 | 11.41 | 2.21 | phenylalanine ammonia-lyase, putative, expressed |
| 01g03360 | 72.47 | 333.91 | 2.20 | BBTI5 - Bowman-Birk type bran trypsin inhibitor precursor, expressed |
| 05g37700 | 4.12 | 18.87 | 2.20 | periplasmic beta-glucosidase precursor, putative, expressed |
| 08g07080 | 4.53 | 20.74 | 2.19 | terpene synthase, putative, expressed |
| 11g34910 | 2.47 | 11.2 | 2.18 | expressed protein |
| 02g32980 | 4.53 | 20.33 | 2.17 | Cupin domain containing protein, expressed |
| 08g35750 | 1.85 | 8.3 | 2.17 | Cupin domain containing protein, expressed |
| 04g34170 | 42.62 | 191.01 | 2.16 | retrotransposon protein, putative, unclassified, expressed |
| 01g67770 | 1.44 | 6.43 | 2.16 | two-component response regulator, putative, expressed |
| 06g49190 | 2.88 | 12.86 | 2.16 | LTPL154-Protease inhibitor/seed storage/LTP family protein precursor |
| 12g26940 | 1.44 | 6.43 | 2.16 | CHASE domain containing protein, expressed |
| 07g03710 | 2.68 | 11.82 | 2.14 | SCP-like extracellular protein, expressed |
| 12g43440 | 2.26 | 9.75 | 2.11 | thaumatin, putative, expressed |
| 01g11730 | 5.76 | 24.68 | 2.10 | GDSL-like lipase/acylhydrolase, putative, expressed |
| 09g38320 | 2.88 | 12.03 | 2.06 | phytoene synthase, chloroplast precursor, putative, expressed |
| 06g07030 | 3.5 | 14.52 | 2.05 | AP2 domain containing protein, expressed |
| 07g31884 | 3.5 | 14.52 | 2.05 | MATE efflux family protein, putative, expressed |
| 08g43040 | 3.91 | 16.18 | 2.05 | transferase family protein, putative, expressed |
| 07g34260 | 29.24 | 120.91 | 2.05 | chalcone and stilbene synthases, putative, expressed |
| 04g41510 | 7.82 | 31.94 | 2.03 | serine/threonine-protein kinase GCN2, putative, expressed |
| 06g20920 | 2.47 | 9.96 | 2.01 | SAM dependent carboxyl methyltransferase, putative, expressed |
| **Table S3. (Continued)** | | | | |
| **Gene** | **TPM-SY** | **TPM-FU** | **log_2_(FU/SY)** | **Description** |
| 03g43510 | 3.71 | 14.93 | 2.01 | expressed protein |
| 01g04050 | 74.94 | 299.69 | 2.00 | BBTI12 - Bowman-Birk type bran trypsin inhibitor precursor |
| 12g07210 | 5.15 | 20.33 | 1.98 | expressed protein |
| 02g27870 | 2.06 | 8.09 | 1.97 | expressed protein |
| 02g06090 | 3.09 | 12.03 | 1.96 | phytosulfokine receptor precursor, putative, expressed |
| 07g01560 | 3.29 | 12.65 | 1.94 | transporter family protein, putative, expressed |
| 06g07070 | 3.09 | 11.82 | 1.94 | BR INSENSITIVE 1-associated receptor kinase 1 precursor |
| 01g62760 | 4.53 | 17.21 | 1.93 | protein phosphatase 2C, putative, expressed |
| 12g07580 | 4.32 | 16.38 | 1.92 | dirigent, putative, expressed |
| 04g49194 | 5.76 | 21.78 | 1.92 | naringenin,2-oxoglutarate 3-dioxygenase, putative, expressed |
| 01g64370 | 3.09 | 11.61 | 1.91 | expressed protein |
| 06g32600 | 2.88 | 10.78 | 1.90 | THION15 - Plant thionin family protein precursor, expressed |
| 07g14150 | 40.15 | 150.16 | 1.90 | cytidine deaminase, putative, expressed |
| 04g27670 | 7.82 | 29.24 | 1.90 | terpene synthase family, metal binding domain containing protein |
| 11g09350 | 2.06 | 7.67 | 1.90 | expressed protein |
| 12g14440 | 424.74 | 1573.1 | 1.89 | Jacalin-like lectin domain containing protein, putative, expressed |
| 10g42020 | 17.5 | 64.71 | 1.89 | RALFL29-Rapid ALkalinization Factor family protein precursor |
| 06g03486 | 11.12 | 41.06 | 1.88 | expressed protein |
| 11g45990 | 18.32 | 67.4 | 1.88 | von Willebrand factor type A domain containing protein |
| 04g52060 | 2.26 | 8.3 | 1.88 | transposable element protein, PF03004, Transposase_24 |
| 12g31880 | 3.09 | 11.2 | 1.86 | translation initiation factor, putative, expressed |
| 04g23940 | 2.47 | 8.92 | 1.85 | chalcone synthase, putative |
| 12g07310 | 2.88 | 10.37 | 1.85 | citrate-binding protein precursor, putative, expressed |
| 12g31820 | 2.88 | 10.37 | 1.85 | phosphoserine phosphatase, chloroplast precursor, putative, expressed |
| 03g12230 | 2.88 | 10.16 | 1.82 | caleosin related protein, putative, expressed |
| 11g17540 | 5.35 | 18.67 | 1.80 | retrotransposon protein, putative, Ty1-copia subclass, expressed |
| 09g31478 | 2.26 | 7.88 | 1.80 | auxin efflux carrier component, putative, expressed |
| 10g42630 | 3.71 | 12.86 | 1.79 | expressed protein |
| 07g01904 | 18.53 | 63.88 | 1.79 | expressed protein |
| 01g61230 | 6.38 | 21.98 | 1.78 | dihydroflavonol-4-reductase, putative, expressed |
| 06g49660 | 4.12 | 14.1 | 1.77 | transferase family protein, putative, expressed |
| 04g59260 | 8.44 | 28.83 | 1.77 | peroxidase precursor, putative, expressed |
| 11g08210 | 7.41 | 25.3 | 1.77 | no apical meristem protein, putative, expressed |
| 01g13690 | 40.35 | 137.71 | 1.77 | ligA, putative, expressed |
| 05g18470 | 4.32 | 14.73 | 1.77 | CRAL/TRIO domain containing protein, expressed |
| 01g15270 | 156.06 | 531.77 | 1.77 | expressed protein |
| 09g26780 | 9.68 | 32.98 | 1.77 | zinc-finger protein, putative, expressed |
| 02g46560 | 2.68 | 9.13 | 1.77 | helix-loop-helix DNA-binding protein, putative, expressed |
| 12g43664 | 2.68 | 9.13 | 1.77 | FGGY family of carbohydrate kinases, putative, expressed |
| 01g70240 | 7.62 | 25.92 | 1.77 | expressed protein |
| 02g37190 | 13.79 | 46.46 | 1.75 | expressed protein |
| **Table S3. (Continued)** | | | | |
| **Gene** | **TPM-SY** | **TPM-FU** | **log_2_(FU/SY)** | **Description** |
| 02g10040 | 2.68 | 8.92 | 1.73 | guanine nucleotide-exchange protein like, putative, expressed |
| 12g34802 | 2.68 | 8.92 | 1.73 | expressed protein |
| 05g02530 | 17.29 | 57.24 | 1.73 | glutathione S-transferase, N-terminal domain containing protein |
| 06g36560 | 11.74 | 38.78 | 1.72 | inositol oxygenase, putative, expressed |
| 12g38180 | 10.71 | 35.05 | 1.71 | heat shock cognate 70 kDa protein 2, putative, expressed |
| 11g37890 | 23.47 | 76.74 | 1.71 | NAD dependent epimerase/dehydratase family protein |
| 01g03310 | 169.65 | 553.75 | 1.71 | BBTI1 - Bowman-Birk type bran trypsin inhibitor precursor |
| 03g50970 | 5.35 | 17.21 | 1.69 | retrotransposon protein, putative, Ty1-copia subclass, expressed |
| 09g36700 | 21.41 | 68.65 | 1.68 | ribonuclease T2 family domain containing protein, expressed |
| 03g58980 | 3.91 | 12.44 | 1.67 | Cupin domain containing protein, expressed |
| 05g45210 | 6.79 | 21.57 | 1.67 | respiratory burst oxidase, putative, expressed |
| 08g12830 | 4.12 | 13.07 | 1.67 | cytidylyltransferase domain containing protein, expressed |
| 10g28080 | 2.88 | 9.13 | 1.66 | glycosyl hydrolase, putative, expressed |
| 03g58300 | 5.76 | 18.25 | 1.66 | indole-3-glycerol phosphate lyase, chloroplast precursor |
| 03g55460 | 3.09 | 9.75 | 1.66 | expressed protein |
| 03g57420 | 3.09 | 9.75 | 1.66 | ML domain protein, putative, expressed |
| 05g05390 | 3.09 | 9.75 | 1.66 | expressed protein |
| 01g74110 | 4.74 | 14.93 | 1.66 | metal cation transporter, putative, expressed |
| 03g20550 | 3.5 | 10.99 | 1.65 | WRKY55-Superfamily of TFs having WRKY and zinc finger domains |
| 02g07160 | 5.35 | 16.59 | 1.63 | glyoxalase family protein, putative, expressed |
| 01g13420 | 5.76 | 17.84 | 1.63 | SOUL heme-binding protein, putative, expressed |
| 01g03390 | 91.21 | 281.85 | 1.63 | BBTI7 - Bowman-Birk type bran trypsin inhibitor precursor, expressed |
| 03g57450 | 3.09 | 9.54 | 1.63 | CAMK_like.2-calcium/calmodulin depedent protein kinases |
| 08g33150 | 3.09 | 9.54 | 1.63 | MYB family transcription factor, putative, expressed |
| 08g14570 | 6.59 | 20.33 | 1.63 | NADPH reductase, putative, expressed |
| 02g32840 | 3.91 | 12.03 | 1.62 | zinc finger A20 and AN1 domain-containing stress-associated protein |
| 01g65992 | 3.71 | 11.41 | 1.62 | expressed protein |
| 10g40990 | 3.71 | 11.41 | 1.62 | flavonol synthase/flavanone 3-hydroxylase, putative, expressed |
| 07g46920 | 25.32 | 77.57 | 1.62 | sex determination protein tasselseed-2, putative, expressed |
| 12g38170 | 122.71 | 373.11 | 1.60 | osmotin, putative, expressed |
| 04g39170 | 3.71 | 11.2 | 1.59 | expressed protein |
| 09g34920 | 10.5 | 31.32 | 1.58 | glycosyl hydrolase family 29, putative, expressed |
| 03g25960 | 25.32 | 75.49 | 1.58 | RNA recognition motif containing protein, putative, expressed |
| 04g42340 | 15.03 | 44.8 | 1.58 | expressed protein |
| 05g25080 | 4.53 | 13.48 | 1.57 | transposon protein, putative, unclassified, expressed |
| 07g34280 | 4.74 | 14.1 | 1.57 | CXE carboxylesterase, putative, expressed |
| 08g14195 | 15.85 | 47.08 | 1.57 | expressed protein |
| 08g39860 | 19.56 | 57.86 | 1.56 | Os8bglu27 - beta-glucosidase homologue |
| 07g14514 | 5.76 | 17.01 | 1.56 | retrotransposon protein, putative, unclassified, expressed |
| 11g44870 | 5.76 | 17.01 | 1.56 | expressed protein |
| 01g67540 | 10.71 | 31.52 | 1.56 | AMP-binding domain containing protein, expressed |
| **Table S3. (Continued)** | | | | |
| **Gene** | **TPM-SY** | **TPM-FU** | **log_2_(FU/SY)** | **Description** |
| 06g08140 | 5.15 | 15.14 | 1.56 | protein phosphatase 2C, putative, expressed |
| 06g12455 | 11.74 | 34.43 | 1.55 | expressed protein |
| 02g09490 | 10.71 | 31.32 | 1.55 | dehydrogenase, putative, expressed |
| 01g68810 | 5.35 | 15.55 | 1.54 | expressed protein |
| 01g67190 | 3.71 | 10.78 | 1.54 | ribonuclease T2 family domain containing protein, expressed |
| 12g36220 | 127.03 | 368.96 | 1.54 | inhibitor I family protein, putative, expressed |
| 03g11290 | 7 | 20.33 | 1.54 | expressed protein |
| 05g36270 | 6.79 | 19.7 | 1.54 | fructose-1,6-bisphosphatase, putative, expressed |
| 04g35840 | 6.18 | 17.84 | 1.53 | T-complex protein 11, putative, expressed |
| 05g49420 | 4.53 | 13.07 | 1.53 | transcription factor, putative, expressed |
| 07g30120 | 5.97 | 17.21 | 1.53 | expressed protein |
| 01g51540 | 4.32 | 12.44 | 1.53 | cytidine/deoxycytidylate deaminase, putative, expressed |
| 06g51060 | 4.32 | 12.44 | 1.53 | CHIT8 - Chitinase family protein precursor, expressed |
| 11g10590 | 8.65 | 24.89 | 1.52 | hypothetical protein |
| 06g16640 | 16.26 | 46.66 | 1.52 | carboxyl-terminal peptidase, putative, expressed |
| 02g01740 | 4.12 | 11.82 | 1.52 | U5 small nuclear ribonucleoprotein 200 kDa helicase |
| 02g55000 | 4.12 | 11.82 | 1.52 | zinc finger CCCH-type with G patch domain-containing protein |
| 01g35789 | 13.59 | 38.37 | 1.50 | expressed protein |
| 03g27840 | 12.35 | 34.84 | 1.50 | splicing factor, arginine/serine-rich 16, putative, expressed |
| 04g17100 | 6.18 | 17.42 | 1.50 | heavy metal-associated domain containing protein, expressed |
| 09g10010 | 23.27 | 65.54 | 1.49 | expressed protein |
| 06g50930 | 8.85 | 24.89 | 1.49 | rhodanese-like domain containing protein, putative, expressed |
| 01g71670 | 7.82 | 21.98 | 1.49 | glycosyl hydrolases family 17, putative, expressed |
| 03g52680 | 24.71 | 69.27 | 1.49 | expressed protein |
| 01g63620 | 30.88 | 86.49 | 1.49 | expressed protein |
| 11g09979 | 4.53 | 12.65 | 1.48 | expressed protein |
| 04g43800 | 7.82 | 21.78 | 1.48 | phenylalanine ammonia-lyase, putative, expressed |
| 07g41410 | 13.79 | 38.37 | 1.48 | EGG APPARATUS-1, putative, expressed |
| 01g18240 | 4.94 | 13.69 | 1.47 | MYB family transcription factor, putative, expressed |
| 05g49140 | 8.03 | 22.19 | 1.47 | CGMC_MAPKCMGC_2.8 - CGMC includes CDA, MAPK, GSK3, and CLKC kinases, expressed |
| 01g11650 | 12.76 | 35.26 | 1.47 | GDSL-like lipase/acylhydrolase, putative, expressed |
| 08g44590 | 3.91 | 10.78 | 1.46 | gibberellin 20 oxidase 2, putative, expressed |
| 01g54860 | 5.97 | 16.38 | 1.46 | enoyl-CoA hydratase/isomerase family protein, putative, expressed |
| 09g32320 | 5.97 | 16.38 | 1.46 | growth regulator related protein, putative, expressed |
| 03g43684 | 7.41 | 20.33 | 1.46 | KIP1, putative, expressed |
| 08g33740 | 9.68 | 26.55 | 1.46 | CSLA11 - cellulose synthase-like family A, expressed |
| 02g49920 | 12.97 | 35.47 | 1.45 | 3-ketoacyl-CoA synthase, putative, expressed |
| 01g16980 | 4.12 | 11.2 | 1.44 | expressed protein |
| 07g49400 | 112.83 | 306.53 | 1.44 | OsAPx2 - Cytosolic Ascorbate Peroxidase encoding gene 4,5,6,8 |
| 09g38790 | 6.59 | 17.84 | 1.44 | ZOS9-19 - C2H2 zinc finger protein, expressed |
| **Table S3. (Continued)** | | | | |
| **Gene** | **TPM-SY** | **TPM-FU** | **log_2_(FU/SY)** | **Description** |
| 01g03340 | 103.77 | 280.4 | 1.43 | BBTI4 - Bowman-Birk type bran trypsin inhibitor precursor |
| 01g25189 | 5.15 | 13.9 | 1.43 | delta14-sterol reductase, putative, expressed |
| 07g29750 | 5.15 | 13.9 | 1.43 | glycosyl hydrolases family 16, putative, expressed |
| 09g12290 | 55.59 | 149.53 | 1.43 | bifunctional aspartokinase/homoserine dehydrogenase |
| 01g62060 | 23.06 | 62.01 | 1.43 | plant-specific domain TIGR01589 family protein, expressed |
| 11g31540 | 6.18 | 16.59 | 1.42 | BR INSENSITIVE 1-associated receptor kinase 1 precursor |
| 06g50950 | 7.21 | 19.29 | 1.42 | GDSL-like lipase/acylhydrolase, putative, expressed |
| 07g48200 | 7.21 | 19.29 | 1.42 | B3 DNA binding domain containing protein, putative, expressed |
| 12g43450 | 17.09 | 45.63 | 1.42 | thaumatin family domain containing protein, expressed |
| 12g43640 | 4.74 | 12.65 | 1.42 | receptor-like protein kinase HAIKU2 precursor, putative, expressed |
| 07g13770 | 13.38 | 35.67 | 1.41 | UDP-glucoronosyl and glucosyl transferase domain containing protein |
| 09g31410 | 5.76 | 15.35 | 1.41 | Os9bglu29 - beta-glucosidase homologue |
| 03g15020 | 15.03 | 40.03 | 1.41 | beta-galactosidase precursor, putative, expressed |
| 12g26290 | 43.85 | 116.77 | 1.41 | alpha-DOX2, putative, expressed |
| 02g58214 | 14 | 37.12 | 1.41 | expressed protein |
| 05g30500 | 57.85 | 153.27 | 1.41 | expressed protein |
| 05g05680 | 5.56 | 14.73 | 1.41 | 1-aminocyclopropane-1-carboxylate oxidase, putative, expressed |
| 10g21230 | 5.56 | 14.73 | 1.41 | ATP synthase C chain, putative, expressed |
| 09g20940 | 7.21 | 19.08 | 1.40 | expressed protein |
| 11g24070 | 4.94 | 13.07 | 1.40 | LTPL10 - Protease inhibitor/seed storage/LTP family protein |
| 02g26370 | 6.59 | 17.42 | 1.40 | wiskott-Aldrich syndrome protein family member 2 |
| 02g05470 | 12.56 | 33.18 | 1.40 | CCT motif family protein, expressed |
| 05g37930 | 4.32 | 11.41 | 1.40 | expressed protein |
| 12g34850 | 5.35 | 14.1 | 1.40 | Fibronectin type III domain containing protein, expressed |
| 07g38830 | 4.74 | 12.44 | 1.39 | hydrolase, alpha/beta fold family domain containing protein |
| 12g35270 | 4.74 | 12.44 | 1.39 | expressed protein |
| 06g46950 | 14.41 | 37.75 | 1.39 | EF hand family protein, putative, expressed |
| 07g01990 | 14.21 | 37.12 | 1.39 | expressed protein |
| 04g39900 | 5.56 | 14.52 | 1.38 | Os4bglu13 - beta-glucosidase homologue |
| 08g30340 | 7 | 18.25 | 1.38 | PAS2, putative, expressed |
| 07g37100 | 11.94 | 31.11 | 1.38 | nucleoside transporter, putative, expressed |
| 06g01760 | 14.21 | 36.92 | 1.38 | ligA, putative, expressed |
| 07g48980 | 15.65 | 40.65 | 1.38 | nicotianamine synthase, putative, expressed |
| 03g13200 | 4.74 | 12.24 | 1.37 | peroxidase precursor, putative, expressed |
| 04g42030 | 4.74 | 12.24 | 1.37 | expressed protein |
| 10g42420 | 11.74 | 30.28 | 1.37 | GTPase-activating protein, putative, expressed |
| 12g02470 | 15.85 | 40.86 | 1.37 | WRKY65-Superfamily of TFs having WRKY and zinc finger domains |
| 04g10460 | 5.15 | 13.27 | 1.37 | amidase, putative, expressed |
| 05g12400 | 8.24 | 21.15 | 1.36 | BURP domain containing protein, expressed |
| 06g11660 | 13.38 | 34.22 | 1.35 | phosphate-induced protein 1 domain containing protein |
| 01g50690 | 5.76 | 14.73 | 1.35 | WD domain, G-beta repeat domain containing protein, expressed |
| **Table S3. (Continued)** | | | | |
| **Gene** | **TPM-SY** | **TPM-FU** | **log_2_(FU/SY)** | **Description** |
| 01g71340 | 23.88 | 60.98 | 1.35 | glycosyl hydrolases family 17, putative, expressed |
| 05g01560 | 18.53 | 47.29 | 1.35 | vacuolar ATP synthase, putative, expressed |
| 09g27580 | 16.26 | 41.48 | 1.35 | potassium transporter, putative, expressed |
| 04g39880 | 9.68 | 24.68 | 1.35 | Os4bglu12 - beta-glucosidase, exo-beta-glucanase, expressed |
| 12g13130 | 8.24 | 20.95 | 1.35 | ZOS12-03 - C2H2 zinc finger protein, expressed |
| 03g10140 | 11.12 | 28.21 | 1.34 | ZOS3-04 - C2H2 zinc finger protein, expressed |
| 02g17390 | 26.35 | 66.78 | 1.34 | 3-hydroxyacyl-CoA dehydrogenase, putative, expressed |
| 02g14480 | 5.76 | 14.52 | 1.33 | receptor-like kinase, putative, expressed |
| 05g09440 | 5.35 | 13.48 | 1.33 | NADP-dependent malic enzyme, chloroplast precursor |
| 07g12340 | 9.47 | 23.85 | 1.33 | NAC domain-containing protein 67, putative, expressed |
| 12g36210 | 114.88 | 289.32 | 1.33 | inhibitor I family protein, putative, expressed |
| 11g10050 | 19.56 | 49.15 | 1.33 | G-protein alpha subunit, putative, expressed |
| 01g49219 | 14.21 | 35.67 | 1.33 | expressed protein |
| 06g05740 | 10.5 | 26.34 | 1.33 | expressed protein |
| 09g17360 | 10.09 | 25.3 | 1.33 | expressed protein |
| 04g49210 | 15.65 | 39.2 | 1.32 | naringenin,2-oxoglutarate 3-dioxygenase, putative, expressed |
| 01g17470 | 5.56 | 13.9 | 1.32 | plastocyanin-like domain containing protein, putative, expressed |
| 03g28990 | 5.56 | 13.9 | 1.32 | zinc finger family protein, putative, expressed |
| 02g11720 | 5.15 | 12.86 | 1.32 | lipase, putative, expressed |
| 03g17540 | 5.15 | 12.86 | 1.32 | TBC domain containing protein, expressed |
| 06g28820 | 9.06 | 22.61 | 1.32 | cycloartenol synthase, putative |
| 03g04060 | 7.41 | 18.46 | 1.32 | CHIT16 - Chitinase family protein precursor, expressed |
| 02g57160 | 7 | 17.42 | 1.32 | ELMO/CED-12 family protein, putative, expressed |
| 04g49410 | 8.85 | 21.78 | 1.30 | expansin precursor, putative, expressed |
| 03g20670 | 22.85 | 56.2 | 1.30 | ELMO/CED-12 family protein, putative, expressed |
| 04g33390 | 14 | 34.43 | 1.30 | prephenate dehydratase domain containing protein, expressed |
| 01g68050 | 7 | 17.21 | 1.30 | transmembrane amino acid transporter protein, putative, expressed |
| 07g48020 | 21.62 | 53.09 | 1.30 | peroxidase precursor, putative, expressed |
| 03g45300 | 6.59 | 16.18 | 1.30 | transposon protein, putative, unclassified, expressed |
| 08g37840 | 17.91 | 43.97 | 1.30 | phosphate-induced protein 1 domain containing protein |
| 03g04680 | 20.38 | 49.98 | 1.29 | cytochrome P450, putative, expressed |
| 06g43600 | 5.35 | 13.07 | 1.29 | LTPL129-Protease inhibitor/seed storage/LTP family protein precursor |
| 04g43490 | 8.85 | 21.57 | 1.29 | CK1_CaseinKinase_1.7 - CK1 includes the casein kinase 1 kinases |
| 01g11860 | 7.41 | 18.04 | 1.28 | DJ-1 family protein, putative, expressed |
| 05g40890 | 5.97 | 14.52 | 1.28 | expressed protein |
| 01g37280 | 7 | 17.01 | 1.28 | expressed protein |
| 02g45710 | 8.03 | 19.5 | 1.28 | zinc finger, C3HC4 type domain containing protein, expressed |
| 02g06360 | 5.56 | 13.48 | 1.28 | CPn_0526/CP_0226/CPj0526/CpB0547 |
| 04g01690 | 5.56 | 13.48 | 1.28 | pyridoxal-dependent decarboxylase protein, putative, expressed |
| 09g12660 | 7.62 | 18.46 | 1.28 | glucose-1-phosphate adenylyltransferase large subunit |
| 09g36680 | 116.53 | 282.06 | 1.28 | ribonuclease T2 family domain containing protein, expressed |
| **Table S3. (Continued)** | | | | |
| **Gene** | **TPM-SY** | **TPM-FU** | **log_2_(FU/SY)** | **Description** |
| 08g10310 | 11.32 | 27.38 | 1.27 | SHR5-receptor-like kinase, putative, expressed |
| 08g34280 | 52.5 | 126.93 | 1.27 | cinnamoyl-CoA reductase, putative, expressed |
| 11g18570 | 24.29 | 58.69 | 1.27 | cytochrome P450, putative, expressed |
| 01g59990 | 9.06 | 21.78 | 1.27 | ribosomal protein L24, putative, expressed |
| 05g49160 | 10.29 | 24.68 | 1.26 | expressed protein |
| 12g03200 | 30.06 | 71.97 | 1.26 | MATE efflux family protein, putative, expressed |
| 12g31450 | 8.24 | 19.7 | 1.26 | plastid division regulator MinE, putative, expressed |
| 02g13380 | 11.12 | 26.55 | 1.26 | early nodulin 93 ENOD93 protein, putative, expressed |
| 06g34730 | 16.68 | 39.82 | 1.26 | expressed protein |
| 03g03034 | 8.44 | 20.12 | 1.25 | flavonol synthase/flavanone 3-hydroxylase, putative, expressed |
| 08g37370 | 22.65 | 53.92 | 1.25 | mitochondrial carrier protein, putative, expressed |
| 07g06830 | 9.68 | 23.02 | 1.25 | gibberellin receptor GID1L2, putative, expressed |
| 08g44450 | 30.27 | 71.97 | 1.25 | L1P family of ribosomal proteins domain containing protein |
| 07g18120 | 6.38 | 15.14 | 1.25 | aldehyde oxidase, putative, expressed |
| 05g38180 | 11.74 | 27.79 | 1.24 | Yip1 domain containing protein, expressed |
| 11g42220 | 8.24 | 19.5 | 1.24 | laccase precursor protein, putative, expressed |
| 01g17050 | 5.97 | 14.1 | 1.24 | VQ domain containing protein, putative, expressed |
| 05g06140 | 12.97 | 30.49 | 1.23 | lipase, putative, expressed |
| 03g49440 | 37.68 | 88.56 | 1.23 | phosphatase, putative, expressed |
| 02g19860 | 8.03 | 18.87 | 1.23 | pre-mRNA-splicing factor ATP-dependent RNA helicase |
| 02g22374 | 6.18 | 14.52 | 1.23 | expressed protein |
| 02g06890 | 16.06 | 37.54 | 1.22 | OTU-like cysteine protease family protein, putative, expressed |
| 11g46000 | 30.68 | 71.55 | 1.22 | von Willebrand factor type A domain containing protein |
| 07g07410 | 6.59 | 15.35 | 1.22 | oxidoreductase, 2OG-Fe oxygenase family protein |
| 10g37850 | 19.56 | 45.42 | 1.22 | armadillo, putative, expressed |
| 01g15320 | 28.82 | 66.78 | 1.21 | RALFL9 - Rapid ALkalinization Factor family protein precursor |
| 08g44210 | 32.94 | 76.32 | 1.21 | dihydroneopterin aldolase, putative, expressed |
| 05g35290 | 28.21 | 65.33 | 1.21 | phenylalanine ammonia-lyase, putative, expressed |
| 02g26400 | 6.18 | 14.31 | 1.21 | nuclease, EndA/NucM family protein, expressed |
| 09g31458 | 7.62 | 17.63 | 1.21 | expressed protein |
| 03g52340 | 6.38 | 14.73 | 1.21 | endoplasmic oxidoreductin-1 precursor, putative, expressed |
| 10g39430 | 9.26 | 21.36 | 1.21 | expressed protein |
| 03g45770 | 11.12 | 25.51 | 1.20 | expressed protein |
| 09g26400 | 17.91 | 41.06 | 1.20 | zinc finger, C3HC4 type domain containing protein, expressed |
| 05g32580 | 8.24 | 18.87 | 1.20 | glycine-rich protein, putative, expressed |
| 02g55060 | 40.77 | 93.33 | 1.19 | cytochrome b5-like Heme/Steroid binding domain containing protein |
| 01g05140 | 7 | 15.97 | 1.19 | expressed protein |
| 01g70110 | 14 | 31.94 | 1.19 | No apical meristem protein, putative, expressed |
| 05g35110 | 7.82 | 17.84 | 1.19 | OsFBL22 - F-box domain and LRR containing protein, expressed |
| 04g38530 | 11.74 | 26.75 | 1.19 | aldose 1-epimerase, putative, expressed |
| 01g40200 | 6.38 | 14.52 | 1.19 | XIK, putative, expressed |
| **Table S3. (Continued)** | | | | |
| **Gene** | **TPM-SY** | **TPM-FU** | **log_2_(FU/SY)** | **Description** |
| 06g16350 | 6.38 | 14.52 | 1.19 | peroxidase precursor, putative, expressed |
| 06g39906 | 29.03 | 65.95 | 1.18 | homeobox domain containing protein, expressed |
| 04g10350 | 31.09 | 70.52 | 1.18 | 1-aminocyclopropane-1-carboxylate oxidase homolog 2 |
| 03g64050 | 9.06 | 20.53 | 1.18 | receptor protein kinase, putative, expressed |
| 05g05600 | 9.06 | 20.53 | 1.18 | ATA15 protein, putative, expressed |
| 03g46440 | 11.74 | 26.55 | 1.18 | BTBA4 - Bric-a-Brac,Tramtrack, Broad Complex BTB domain with Ankyrin repeat region |
| 03g59310 | 79.47 | 179.61 | 1.18 | ribosomal protein, putative, expressed |
| 03g21720 | 48.38 | 108.88 | 1.17 | vacuolar-sorting receptor precursor, putative, expressed |
| 04g45290 | 8.03 | 18.04 | 1.17 | glycosyl hydrolases, putative, expressed |
| 10g40934 | 18.74 | 42.1 | 1.17 | flavonol synthase/flavanone 3-hydroxylase, putative, expressed |
| 03g43720 | 10.91 | 24.47 | 1.17 | transporter family protein, putative, expressed |
| 09g28910 | 9.26 | 20.74 | 1.16 | carbonic anhydrase, chloroplast precursor, putative, expressed |
| 03g60260 | 7.41 | 16.59 | 1.16 | ANT1, putative, expressed |
| 12g41600 | 8.44 | 18.87 | 1.16 | OsSAUR57 - Auxin-responsive SAUR gene family member |
| 02g46850 | 11.32 | 25.3 | 1.16 | oligopeptide transporter, putative, expressed |
| 12g36670 | 9.47 | 21.15 | 1.16 | F-box/LRR-repeat protein 3, putative, expressed |
| 04g43820 | 7.62 | 17.01 | 1.16 | expressed protein |
| 09g31486 | 7.62 | 17.01 | 1.16 | DnaK family protein, putative, expressed |
| 08g44850 | 46.74 | 104.11 | 1.16 | C2 domain containing protein, putative, expressed |
| 08g30060 | 11.74 | 26.13 | 1.15 | proton pump interactor, putative, expressed |
| 01g53600 | 7 | 15.55 | 1.15 | farnesyltransferase subunit beta, putative, expressed |
| 01g14932 | 12.15 | 26.96 | 1.15 | NAK-like ser/thr protein kinase, putative, expressed |
| 05g48870 | 11.32 | 25.1 | 1.15 | auxin response factor 15, putative, expressed |
| 01g47080 | 13.59 | 30.07 | 1.15 | pyruvate kinase, putative, expressed |
| 09g36860 | 12.97 | 28.62 | 1.14 | acyl carrier protein, putative, expressed |
| 04g53502 | 7.62 | 16.8 | 1.14 | expressed protein |
| 06g21270 | 61.56 | 135.43 | 1.14 | glycine rich protein family protein, putative, expressed |
| 05g12630 | 402.09 | 884.55 | 1.14 | expressed protein |
| 07g39510 | 9.06 | 19.91 | 1.14 | yippee zinc-binding protein, putative, expressed |
| 03g22810 | 25.32 | 55.58 | 1.13 | copper/zinc superoxide dismutase, putative, expressed |
| 12g02250 | 11.74 | 25.72 | 1.13 | STE_PAK_Ste20_Slob_Wnk.3 - STE kinases include homologs to sterile 7, sterile 11 and sterile 20 from yeast, expressed |
| 01g42410 | 13.38 | 29.24 | 1.13 | pleiotropic drug resistance protein, putative, expressed |
| 04g41640 | 38.29 | 83.58 | 1.13 | HEV2 - Hevein family protein precursor, expressed |
| 07g29770 | 8.65 | 18.87 | 1.13 | zinc finger protein, putative, expressed |
| 03g10030 | 8.85 | 19.29 | 1.12 | membrane associated DUF588 domain containing protein |
| 06g41360 | 7.82 | 17.01 | 1.12 | phosphoribosyl transferase, putative, expressed |
| 12g03810 | 11.74 | 25.51 | 1.12 | CAMK_KIN1/SNF1/Nim1_like.37 - CAMK includes calcium/calmodulin depedent protein kinases, expressed |
| 05g08640 | 19.97 | 43.35 | 1.12 | transferase family protein, putative, expressed |
| **Table S3. (Continued)** | | | | |
| **Gene** | **TPM-SY** | **TPM-FU** | **log_2_(FU/SY)** | **Description** |
| 01g55100 | 9.68 | 20.95 | 1.11 | slTCP3, putative, expressed |
| 11g10520 | 28.41 | 61.39 | 1.11 | dehydrogenase, putative, expressed |
| 02g48150 | 12.97 | 28 | 1.11 | expressed protein |
| 02g39840 | 10.5 | 22.61 | 1.11 | eukaryotic initiation factor iso-4F subunit p82-34, putative, expressed |
| 07g44004 | 10.5 | 22.61 | 1.11 | expressed protein |
| 01g12320 | 13.59 | 29.24 | 1.11 | GDSL-like lipase/acylhydrolase, putative, expressed |
| 07g27490 | 14 | 30.07 | 1.10 | Regulator of chromosome condensation domain containing protein |
| 03g04660 | 34.38 | 73.83 | 1.10 | cytochrome P450 86A1, putative, expressed |
| 02g53530 | 13.59 | 29.04 | 1.10 | ZOS2-17 - C2H2 zinc finger protein, expressed |
| 09g29460 | 52.71 | 112.41 | 1.09 | homeobox associated leucine zipper, putative, expressed |
| 05g13580 | 21.41 | 45.63 | 1.09 | OsCML18 - Calmodulin-related calcium sensor protein, expressed |
| 01g02010 | 14.41 | 30.69 | 1.09 | expressed protein |
| 11g03970 | 12.76 | 27.17 | 1.09 | CAMK_KIN1/SNF1/Nim1_like.5 - CAMK includes calcium/calmodulin depedent protein kinases, expressed |
| 02g04250 | 18.12 | 38.58 | 1.09 | glycosyltransferase, putative, expressed |
| 02g17360 | 13.18 | 28 | 1.09 | PPR repeat domain containing protein, putative, expressed |
| 02g01500 | 7.82 | 16.59 | 1.09 | 2-oxo acid dehydrogenases acyltransferase domain containing protein |
| 01g59970 | 11.74 | 24.89 | 1.08 | expressed protein |
| 03g53800 | 168.21 | 356.52 | 1.08 | periplasmic beta-glucosidase precursor, putative, expressed |
| 04g49160 | 10.09 | 21.36 | 1.08 | zinc finger, C3HC4 type domain containing protein, expressed |
| 05g49890 | 140.41 | 296.58 | 1.08 | ras-related protein, putative, expressed |
| 08g02230 | 31.91 | 67.4 | 1.08 | FAD-binding and arabino-lactone oxidase domains containing protein |
| 12g37650 | 193.94 | 409.61 | 1.08 | DUF538 domain containing protein, putative, expressed |
| 01g35050 | 22.65 | 47.7 | 1.07 | early-responsive to dehydration protein-related, putative, expressed |
| 06g49760 | 18.12 | 38.16 | 1.07 | invertase/pectin methylesterase inhibitor family protein |
| 08g07430 | 11.74 | 24.68 | 1.07 | expressed protein |
| 03g60509 | 23.68 | 49.78 | 1.07 | expressed protein |
| 03g32314 | 40.97 | 86.07 | 1.07 | allene oxide cyclase 4, chloroplast precursor, putative, expressed |
| 03g61920 | 9.68 | 20.33 | 1.07 | electron transfer flavoprotein subunit alpha, mitochondrial precursor |
| 07g13234 | 9.68 | 20.33 | 1.07 | expressed protein |
| 04g01710 | 12.15 | 25.51 | 1.07 | cysteine proteinase At4g11310 precursor, putative, expressed |
| 07g36630 | 14.62 | 30.69 | 1.07 | CSLF8-cellulose synthase-like family F; beta1,3;1,4 glucan synthase |
| 07g25810 | 16.26 | 34.01 | 1.06 | retrotransposon protein, putative, unclassified, expressed |
| 03g51080 | 78.24 | 163.43 | 1.06 | glutamate decarboxylase, putative, expressed |
| 06g03660 | 22.85 | 47.7 | 1.06 | peroxisomal biogenesis factor 11, putative, expressed |
| 10g08550 | 71.24 | 148.7 | 1.06 | enolase, putative, expressed |
| 02g13060 | 154.83 | 323.13 | 1.06 | expressed protein |
| 05g48690 | 11.53 | 24.06 | 1.06 | transcription factor like protein, putative, expressed |
| 06g34440 | 20.38 | 42.52 | 1.06 | dnaJ domain containing protein, expressed |
| 04g38914 | 24.09 | 50.19 | 1.06 | expressed protein |
| 05g13940 | 684.98 | 1426.3 | 1.06 | retrotransposon protein, putative, unclassified, expressed |
| **Table S3. (Continued)** | | | | |
| **Gene** | **TPM-SY** | **TPM-FU** | **log_2_(FU/SY)** | **Description** |
| 01g19940 | 24.91 | 51.85 | 1.06 | expressed protein |
| 07g11290 | 16.47 | 34.22 | 1.05 | expressed protein |
| 10g42950 | 10.29 | 21.36 | 1.05 | cyclin-dependent kinase E-1, putative, expressed |
| 05g09020 | 13.79 | 28.62 | 1.05 | WRKY67-Superfamily of TFs having WRKY and zinc finger domains |
| 01g65680 | 10.91 | 22.61 | 1.05 | 4,5-DOPA dioxygenase extradiol, putative, expressed |
| 03g57349 | 56.62 | 117.18 | 1.05 | expressed protein |
| 08g40560 | 12.15 | 25.1 | 1.05 | ZOS8-11 - C2H2 zinc finger protein, expressed |
| 06g01934 | 12.35 | 25.51 | 1.05 | homeobox domain containing protein, expressed |
| 03g01960 | 34.38 | 70.93 | 1.04 | expressed protein |
| 10g38740 | 17.29 | 35.67 | 1.04 | glutathione S-transferase, putative, expressed |
| 11g47809 | 82.15 | 169.44 | 1.04 | metallothionein, putative, expressed |
| 06g03640 | 17.91 | 36.92 | 1.04 | BAG domain containing protein, expressed |
| 07g04990 | 89.56 | 184.17 | 1.04 | oxidoreductase, aldo/keto reductase family protein, putative |
| 03g03590 | 11.12 | 22.81 | 1.04 | transporter, monovalent cation:proton antiporter-2 family |
| 01g61580 | 18.12 | 37.12 | 1.03 | selT-like protein precursor, putative, expressed |
| 04g34940 | 12.56 | 25.72 | 1.03 | protein of unknown function domain containing protein, expressed |
| 01g71770 | 21.21 | 43.35 | 1.03 | RNA recognition motif containing protein, putative, expressed |
| 02g18830 | 14.21 | 29.04 | 1.03 | ribosome biogenesis protein NEP1, putative, expressed |
| 03g49720 | 15.03 | 30.69 | 1.03 | PAP fibrillin family domain containing protein, expressed |
| 05g47560 | 23.68 | 48.32 | 1.03 | serine/threonine-protein kinase SNT7, chloroplast precursor |
| 02g39560 | 9.88 | 20.12 | 1.03 | non-receptor tyrosine kinase spore lysis A, putative, expressed |
| 02g29480 | 11.12 | 22.61 | 1.02 | RNA-binding protein-like, putative, expressed |
| 03g29190 | 11.12 | 22.61 | 1.02 | PDI, putative, expressed |
| 01g09540 | 88.94 | 180.64 | 1.02 | HAD superfamily phosphatase, putative, expressed |
| 11g02424 | 70.21 | 142.28 | 1.02 | LTPL9 - Protease inhibitor/seed storage/LTP family protein precursor |
| 02g06290 | 10.29 | 20.74 | 1.01 | rhodanese-like domain containing protein, putative, expressed |
| 01g58114 | 15.85 | 31.94 | 1.01 | expressed protein |
| 03g21830 | 15.03 | 30.28 | 1.01 | appr-1-p processing enzyme family protein, putative, expressed |
| 04g35220 | 15.24 | 30.69 | 1.01 | expressed protein |
| 06g03860 | 11.74 | 23.64 | 1.01 | uncharacterized membrane protein, putative, expressed |
| 08g20544 | 11.74 | 23.64 | 1.01 | expressed protein |
| 01g74020 | 187.97 | 377.67 | 1.01 | MYB family transcription factor, putative, expressed |
| 01g65380 | 30.06 | 60.35 | 1.01 | patellin protein, putative, expressed |
| 08g17600 | 20.18 | 40.44 | 1.00 | SNARE domain containing protein, putative, expressed |
| 11g36340 | 33.15 | 66.37 | 1.00 | lymphoid organ expressed yellow head virus receptor protein |
| 07g39560 | 43.03 | 86.07 | 1.00 | RNA recognition motif containing protein, putative, expressed |
| 12g33130 | 13.18 | 0.41 | -5.01 | expressed protein |
| 02g22020 | 11.74 | 0.41 | -4.84 | MYB family transcription factor, putative, expressed |
| 09g38450 | 5.76 | 0.41 | -3.81 | expressed protein |
| 02g11705 | 5.15 | 0.41 | -3.65 | expressed protein |
| 01g63500 | 4.94 | 0.41 | -3.59 | expressed protein |
| **Table S3. (Continued)** | | | | |
| **Gene** | **TPM-SY** | **TPM-FU** | **log_2_(FU/SY)** | **Description** |
| 05g05460 | 4.74 | 0.41 | -3.53 | EF hand family protein, putative, expressed |
| 04g15840 | 4.12 | 0.41 | -3.33 | expansin precursor, putative, expressed |
| 06g22460 | 4.12 | 0.41 | -3.33 | disease resistance protein RPM1, putative, expressed |
| 04g08630 | 28.41 | 3.53 | -3.01 | expressed protein |
| 09g31130 | 11.32 | 1.45 | -2.96 | citrate transporter, putative, expressed |
| 06g50340 | 4.74 | 0.62 | -2.93 | receptor protein kinase CLAVATA1 precursor, putative, expressed |
| 12g05890 | 6.59 | 1.04 | -2.66 | expressed protein |
| 09g19650 | 9.06 | 1.45 | -2.64 | 3-ketoacyl-CoA synthase precursor, putative, expressed |
| 07g06860 | 11.53 | 1.87 | -2.62 | gibberellin receptor GID1L2, putative, expressed |
| 10g10130 | 26.35 | 5.18 | -2.35 | OsWAK112d - OsWAK receptor-like protein kinase, expressed |
| 09g19570 | 6.18 | 1.24 | -2.32 | AGAP009532-PA, putative, expressed |
| 04g06879 | 8.24 | 1.66 | -2.31 | hypothetical protein |
| 06g33390 | 7.82 | 1.66 | -2.24 | avr9/Cf-9 rapidly elicited protein 194, putative, expressed |
| 08g37390 | 8.24 | 1.87 | -2.14 | cyclin, putative, expressed |
| 03g44290 | 12.56 | 2.9 | -2.11 | expansin precursor, putative, expressed |
| 08g41489 | 14 | 3.32 | -2.08 | expressed protein |
| 04g17479 | 10.09 | 2.49 | -2.02 | expressed protein |
| 07g23520 | 88.32 | 22.4 | -1.98 | expressed protein |
| 03g45760 | 9.68 | 2.49 | -1.96 | expressed protein |
| 06g47770 | 8.85 | 2.28 | -1.96 | expressed protein |
| 03g02860 | 12.76 | 3.32 | -1.94 | heavy metal-associated domain containing protein, expressed |
| 02g11790 | 32.74 | 8.71 | -1.91 | OsFBK4 - F-box domain and kelch repeat containing protein |
| 03g23970 | 15.44 | 4.15 | -1.90 | diphthine synthase, putative, expressed |
| 03g50280 | 11.53 | 3.11 | -1.89 | GLTP domain containing protein, putative, expressed |
| 08g10010 | 31.29 | 8.5 | -1.88 | acyl-desaturase, chloroplast precursor, putative, expressed |
| 03g47940 | 29.44 | 8.09 | -1.86 | GDSL-like lipase/acylhydrolase, putative, expressed |
| 06g45100 | 13.18 | 3.73 | -1.82 | FAD binding domain of DNA photolyase domain containing protein |
| 06g06180 | 8.03 | 2.28 | -1.82 | transferase family protein, putative, expressed |
| 08g03310 | 51.06 | 14.52 | -1.81 | zinc finger family protein, putative, expressed |
| 12g06660 | 10.91 | 3.11 | -1.81 | actin, putative, expressed |
| 02g13660 | 45.91 | 13.27 | -1.79 | meiosis 5, putative, expressed |
| 09g37650 | 8.44 | 2.49 | -1.76 | flavin-containing monooxygenase family protein |
| 10g01570 | 12.76 | 3.94 | -1.70 | C-5 cytosine-specific DNA methylase, putative, expressed |
| 04g51454 | 16.47 | 5.18 | -1.67 | expressed protein |
| 03g15790 | 9.06 | 2.9 | -1.64 | ZOS3-08 - C2H2 zinc finger protein, expressed |
| 09g24840 | 9.06 | 2.9 | -1.64 | GASR10-GA-regulated GASA/GAST/Snakin family protein precursor |
| 02g16880 | 9.68 | 3.11 | -1.64 | DUF502 domain containing protein |
| 03g11540 | 19.35 | 6.22 | -1.64 | RPA1B - Putative single-stranded DNA binding complex subunit 1 |
| 01g74670 | 16.06 | 5.18 | -1.63 | expressed protein |
| 02g15594 | 26.77 | 8.71 | -1.62 | protein phosphatase 2C, putative, expressed |
| 01g10400 | 68.15 | 22.19 | -1.62 | expressed protein |
| **Table S3. (Continued)** | | | | |
| **Gene** | **TPM-SY** | **TPM-FU** | **log_2_(FU/SY)** | **Description** |
| 07g24090 | 9.47 | 3.11 | -1.61 | mTERF domain containing protein, expressed |
| 07g31650 | 11.32 | 3.73 | -1.60 | expressed protein |
| 01g50410 | 10.71 | 3.53 | -1.60 | STE_MEKK_ste11_MAP3K.6 - STE kinases include homologs to sterile 7, sterile 11 and sterile 20 from yeast, expressed |
| 08g44360 | 29.24 | 9.75 | -1.58 | male sterility protein 2, putative, expressed |
| 05g48150 | 9.88 | 3.32 | -1.57 | transposon protein, putative, unclassified, expressed |
| 06g50330 | 18.32 | 6.22 | -1.56 | senescence-associated protein, putative, expressed |
| 08g03370 | 17.09 | 5.81 | -1.56 | Divergent PAP2 family domain containing protein, expressed |
| 01g36460 | 10.91 | 3.73 | -1.55 | MYB family transcription factor, putative, expressed |
| 03g04070 | 10.29 | 3.53 | -1.54 | no apical meristem protein, putative, expressed |
| 02g42380 | 13.79 | 4.77 | -1.53 | TCP family transcription factor, putative, expressed |
| 12g09720 | 65.88 | 22.81 | -1.53 | dirigent, putative |
| 10g26060 | 16.68 | 5.81 | -1.52 | glutelin, putative, expressed |
| 08g36480 | 75.56 | 26.55 | -1.51 | nitrate reductase, putative, expressed |
| 02g45930 | 162.86 | 57.45 | -1.50 | expressed protein |
| 02g08220 | 15.24 | 5.39 | -1.50 | expressed protein |
| 11g40540 | 17.5 | 6.22 | -1.49 | transporter family protein, putative, expressed |
| 10g39740 | 52.5 | 18.87 | -1.48 | glutathione S-transferase, putative, expressed |
| 01g11710 | 37.47 | 13.48 | -1.47 | GDSL-like lipase/acylhydrolase, putative, expressed |
| 10g40810 | 11.53 | 4.15 | -1.47 | GATA zinc finger domain containing protein, expressed |
| 03g08520 | 12.97 | 4.77 | -1.44 | DUF581 domain containing protein, expressed |
| 11g41034 | 54.56 | 20.33 | -1.42 | expressed protein |
| 04g21350 | 48.59 | 18.25 | -1.41 | flowering promoting factor-like 1, putative, expressed |
| 03g49430 | 17.09 | 6.43 | -1.41 | pre-mRNA-splicing factor, putative, expressed |
| 01g43230 | 39.32 | 14.93 | -1.40 | expressed protein |
| 08g42700 | 13.59 | 5.18 | -1.39 | resistance protein, putative, expressed |
| 03g40310 | 12.97 | 4.98 | -1.38 | RNA recognition motif containing protein, putative, expressed |
| 12g39830 | 19.35 | 7.47 | -1.37 | cyclin, putative, expressed |
| 03g07100 | 20.38 | 7.88 | -1.37 | LTPL82-Protease inhibitor/seed storage/LTP family protein precursor |
| 07g48830 | 17.09 | 6.64 | -1.36 | glycosyl transferase 8 domain containing protein |
| 02g13910 | 14.21 | 5.6 | -1.34 | retrotransposon protein, putative, Ty1-copia subclass |
| 06g34400 | 14.21 | 5.6 | -1.34 | zinc finger, C3HC4 type domain containing protein, expressed |
| 09g25314 | 12.56 | 4.98 | -1.33 | cytochrome c oxidase copper chaperone, putative, expressed |
| 12g20390 | 26.15 | 10.37 | -1.33 | expressed protein |
| 02g52040 | 13.59 | 5.39 | -1.33 | phosphate-induced protein 1domain containing protein |
| 03g62670 | 30.06 | 12.03 | -1.32 | expressed protein |
| 06g50230 | 167.18 | 66.99 | -1.32 | expressed protein |
| 08g08250 | 13.38 | 5.39 | -1.31 | expressed protein |
| 12g13940 | 18.94 | 7.67 | -1.30 | DNA-binding storekeeper protein-related, putative, expressed |
| 07g07930 | 17.91 | 7.26 | -1.30 | LTPL78-Protease inhibitor/seed storage/LTP family protein precursor |
| 03g11560 | 17.71 | 7.26 | -1.29 | digalactosyldiacylglycerol synthase, chloroplast precursor |
| **Table S3. (Continued)** | | | | |
| **Gene** | **TPM-SY** | **TPM-FU** | **log_2_(FU/SY)** | **Description** |
| 05g02300 | 30.68 | 12.65 | -1.28 | Core histone H2A/H2B/H3/H4 domain containing protein |
| 04g33220 | 158.53 | 65.54 | -1.27 | SAM domain family protein, expressed |
| 03g02290 | 18.53 | 7.67 | -1.27 | kinesin motor domain containing protein |
| 06g06510 | 87.3 | 36.29 | -1.27 | histone H3, putative, expressed |
| 03g28080 | 70.82 | 29.45 | -1.27 | ring-H2 zinc finger protein, putative, expressed |
| 09g37240 | 18.74 | 7.88 | -1.25 | glutathione S-transferase, C-terminal domain containing protein |
| 03g24520 | 20.18 | 8.5 | -1.25 | Mo25, putative, expressed |
| 08g25820 | 16.68 | 7.05 | -1.24 | myb-like DNA-binding domain containing protein, expressed |
| 11g05550 | 15.65 | 6.64 | -1.24 | expressed protein |
| 02g03710 | 78.44 | 33.39 | -1.23 | UP-9A, putative, expressed |
| 04g52100 | 94.3 | 40.24 | -1.23 | peptidase, M24 family protein, putative, expressed |
| 02g46910 | 39.74 | 17.21 | -1.21 | glycosyl hydrolases family 16, putative, expressed |
| 08g01100 | 26.77 | 11.61 | -1.21 | HMG1/2, putative, expressed |
| 01g54700 | 24.71 | 10.78 | -1.20 | retrotransposon protein, putative, unclassified, expressed |
| 02g12750 | 25.53 | 11.2 | -1.19 | tetraspanin family protein, putative, expressed |
| 04g31760 | 33.97 | 14.93 | -1.19 | expressed protein |
| 10g28020 | 46.53 | 20.53 | -1.18 | OsPOP21 - Putative Prolyl Oligopeptidase homologue, expressed |
| 02g57980 | 21 | 9.33 | -1.17 | DEAD/DEAH box helicase, putative, expressed |
| 10g42190 | 29.85 | 13.27 | -1.17 | leucine rich repeat containing protein, expressed |
| 03g59040 | 23.27 | 10.37 | -1.17 | squalene synthetase, putative, expressed |
| 04g57190 | 16.26 | 7.26 | -1.16 | ubiquitin carboxyl-terminal hydrolase, family 1, putative, expressed |
| 10g27330 | 27.38 | 12.24 | -1.16 | glycerol-3-phosphate acyltransferase, putative, expressed |
| 01g16970 | 55.59 | 24.89 | -1.16 | glucosidase II beta subunit-like domain containing protein, expressed |
| 08g40090 | 22.24 | 9.96 | -1.16 | leucine rich repeat containing protein, expressed |
| 03g55030 | 24.5 | 10.99 | -1.16 | UDP-glucoronosyl and glucosyl transferase domain containing protein |
| 07g08500 | 17.09 | 7.67 | -1.16 | C-5 cytosine-specific DNA methylase, putative, expressed |
| 01g41565 | 36.44 | 16.38 | -1.15 | ATP-binding domain-containing protein, putative |
| 01g71170 | 44.68 | 20.12 | -1.15 | classical arabinogalactan protein 26 precursor, putative, expressed |
| 02g47150 | 15.65 | 7.05 | -1.15 | DNA topoisomerase 2, putative, expressed |
| 01g68140 | 26.15 | 11.82 | -1.15 | expressed protein |
| 03g63950 | 1706.4 | 774.01 | -1.14 | plastid-specific 30S ribosomal protein 1, chloroplast precursor |
| 03g55410 | 22.85 | 10.37 | -1.14 | peroxidase precursor, putative, expressed |
| 05g11320 | 412.18 | 187.07 | -1.14 | metallothionein-like protein 3B, putative, expressed |
| 08g30554 | 51.06 | 23.23 | -1.14 | expressed protein |
| 12g33120 | 1049 | 477.43 | -1.14 | expressed protein |
| 10g40614 | 38.5 | 17.63 | -1.13 | LTPL147-Protease inhibitor/seed storage/LTP family protein |
| 04g43650 | 15.85 | 7.26 | -1.13 | L-allo-threonine aldolase, putative, expressed |
| 03g06510 | 25.53 | 11.82 | -1.11 | KIP1, putative, expressed |
| 04g50970 | 203.83 | 94.57 | -1.11 | seed specific protein Bn15D1B, putative, expressed |
| 08g01040 | 18.32 | 8.5 | -1.11 | zinc finger, C3HC4 type domain containing protein, expressed |
| 03g63520 | 34.38 | 15.97 | -1.11 | Der1-like family domain containing protein, expressed |
| **Table S3. (Continued)** | | | | |
| **Gene** | **TPM-SY** | **TPM-FU** | **log_2_(FU/SY)** | **Description** |
| 08g31814 | 29.44 | 13.69 | -1.10 | OsAPRL4 adenosine 5'-phosphosulfate reductase-like OsAPRL4 |
| 02g46473 | 22.24 | 10.37 | -1.10 | expressed protein |
| 12g34500 | 36.85 | 17.21 | -1.10 | expressed protein |
| 04g49550 | 19.97 | 9.33 | -1.10 | RING-H2 finger protein ATL2A, putative, expressed |
| 04g46730 | 26.15 | 12.24 | -1.10 | thioesterase family protein, putative, expressed |
| 02g06370 | 19.77 | 9.33 | -1.08 | whirly transcription factor domain containing protein, expressed |
| 04g53950 | 23.68 | 11.2 | -1.08 | glycosyl hydrolases family 16 protein, protein, expressed |
| 01g63690 | 17.09 | 8.09 | -1.08 | hs1, putative, expressed |
| 08g37810 | 51.88 | 24.68 | -1.07 | transcription factor like protein, putative, expressed |
| 08g10510 | 37.06 | 17.63 | -1.07 | aminotransferase, putative, expressed |
| 09g38020 | 288.65 | 137.51 | -1.07 | Core histone H2A/H2B/H3/H4 domain containing protein |
| 02g45225 | 110.35 | 52.68 | -1.07 | expressed protein |
| 12g25120 | 72.06 | 34.43 | -1.07 | core histone H2A/H2B/H3/H4, putative, expressed |
| 03g47949 | 21.21 | 10.16 | -1.06 | HECT-domain domain containing protein, expressed |
| 03g13130 | 21.62 | 10.37 | -1.06 | ternary complex factor MIP1, putative, expressed |
| 05g31140 | 88.94 | 42.72 | -1.06 | glycosyl hydrolases family 17, putative, expressed |
| 02g49870 | 328.8 | 158.04 | -1.06 | expressed protein |
| 04g46830 | 192.3 | 92.91 | -1.05 | LTPL122-Protease inhibitor/seed storage/LTP family protein precursor |
| 02g57720 | 20.59 | 9.96 | -1.05 | aquaporin protein, putative, expressed |
| 07g02200 | 21.41 | 10.37 | -1.05 | plastocyanin-like domain containing protein, putative, expressed |
| 09g16510 | 43.24 | 20.95 | -1.05 | WRKY74-Superfamily of TFs having WRKY and zinc finger domains |
| 06g28480 | 22.65 | 10.99 | -1.04 | polygalacturonase inhibitor 1 precursor, putative, expressed |
| 03g41060 | 17.91 | 8.71 | -1.04 | GASR2- GA-regulated GASA/GAST/Snakin family protein precursor |
| 01g54400 | 69.8 | 34.01 | -1.04 | VQ domain containing protein, putative, expressed |
| 09g20590 | 19.56 | 9.54 | -1.04 | CD2-binding protein-related, putative, expressed |
| 10g36650 | 45.71 | 22.4 | -1.03 | actin, putative, expressed |
| 05g11810 | 78.24 | 38.37 | -1.03 | gibberellin 2-beta-dioxygenase 1, putative, expressed |
| 06g36390 | 48.59 | 23.85 | -1.03 | expressed protein |
| 04g44650 | 24.5 | 12.03 | -1.03 | ferredoxin-thioredoxin reductase, variable chain, putative, expressed |
| 11g29700 | 28.62 | 14.1 | -1.02 | expressed protein |
| 08g06230 | 32.74 | 16.18 | -1.02 | nucleolar GTP-binding protein 1, putative, expressed |
| 01g59920 | 157.71 | 77.98 | -1.02 | cysteine synthase, chloroplast precursor, putative, expressed |
| 02g17940 | 23.47 | 11.61 | -1.02 | leucoanthocyanidin dioxygenase, putative, expressed |
| 07g45130 | 25.12 | 12.44 | -1.01 | hypothetical protein |
| 04g11390 | 29.65 | 14.73 | -1.01 | expressed protein |
| 01g64270 | 218.03 | 108.68 | -1.00 | expressed protein |
| 06g40150 | 39.94 | 19.91 | -1.00 | AP2 domain containing protein, expressed |

**Table S3. (Continued)**

| **Gene** | **TPM-SY** | **TPM-FR** | **log_2_(FR/SY)** | **Description** |
| --- | --- | --- | --- | --- |
| 04g23940 | 2.47 | 41.1 | 4.06 | chalcone synthase, putative |
| 09g26210 | 0.82 | 7.38 | 3.17 | ZOS9-12 - C2H2 zinc finger protein, expressed |
| 08g04300 | 1.03 | 6.38 | 2.63 | fringe-related protein, putative, expressed |
| 03g43100 | 1.85 | 11.37 | 2.62 | expressed protein |
| 01g38610 | 5.15 | 27.53 | 2.42 | helix-loop-helix DNA-binding domain containing protein, expressed |
| 01g64110 | 1.24 | 6.58 | 2.41 | glycosyl hydrolase, putative, expressed |
| 04g52440 | 1.24 | 6.58 | 2.41 | aminotransferase, putative, expressed |
| 01g49710 | 1.44 | 7.18 | 2.32 | glutathione S-transferase, putative, expressed |
| 10g37160 | 1.65 | 8.18 | 2.31 | transposon protein, putative, unclassified, expressed |
| 03g55460 | 3.09 | 14.37 | 2.22 | expressed protein |
| 01g50940 | 1.65 | 7.18 | 2.12 | helix-loop-helix DNA-binding domain containing protein, expressed |
| 02g41670 | 2.47 | 10.38 | 2.07 | phenylalanine ammonia-lyase, putative, expressed |
| 02g33600 | 2.06 | 8.38 | 2.02 | VQ domain containing protein, putative |
| 07g01620 | 2.06 | 8.38 | 2.02 | dirigent, putative, expressed |
| 03g45770 | 11.12 | 44.49 | 2.00 | expressed protein |
| 03g04650 | 4.32 | 16.76 | 1.96 | cytochrome P450 protein, putative, expressed |
| 09g36700 | 21.41 | 82.6 | 1.95 | ribonuclease T2 family domain containing protein, expressed |
| 02g27340 | 4.74 | 18.16 | 1.94 | riboflavin biosynthesis protein ribD, putative, expressed |
| 05g35110 | 7.82 | 29.73 | 1.93 | OsFBL22 - F-box domain and LRR containing protein, expressed |
| 02g38080 | 4.32 | 16.36 | 1.92 | protein kinase domain containing protein, expressed |
| 11g34910 | 2.47 | 8.78 | 1.83 | expressed protein |
| 02g02780 | 2.47 | 8.58 | 1.80 | protein kinase family protein |
| 07g48200 | 7.21 | 24.94 | 1.79 | B3 DNA binding domain containing protein, putative, expressed |
| 08g28790 | 3.29 | 11.37 | 1.79 | dirigent, putative, expressed |
| 10g42630 | 3.71 | 12.77 | 1.78 | expressed protein |
| 06g07030 | 3.5 | 11.77 | 1.75 | AP2 domain containing protein, expressed |
| 01g49490 | 3.09 | 10.18 | 1.72 | expressed protein |
| 11g09979 | 4.53 | 14.57 | 1.69 | expressed protein |
| 01g07980 | 3.71 | 11.37 | 1.62 | ankyrin, putative, expressed |
| 04g41510 | 7.82 | 23.74 | 1.60 | serine/threonine-protein kinase GCN2, putative, expressed |
| 01g09540 | 88.94 | 260.18 | 1.55 | HAD superfamily phosphatase, putative, expressed |
| 06g16640 | 16.26 | 47.29 | 1.54 | carboxyl-terminal peptidase, putative, expressed |
| 01g72350 | 5.97 | 17.36 | 1.54 | amidohydrolase, putative, expressed |
| 01g37280 | 7 | 20.35 | 1.54 | expressed protein |
| 05g45210 | 6.79 | 19.55 | 1.53 | respiratory burst oxidase, putative, expressed |
| 02g38240 | 5.35 | 15.16 | 1.50 | rhodanese family protein, putative, expressed |
| 07g32680 | 5.15 | 14.57 | 1.50 | retrotransposon protein, putative, unclassified, expressed |
| 01g11860 | 7.41 | 20.95 | 1.50 | DJ-1 family protein, putative, expressed |
| 03g58300 | 5.76 | 16.16 | 1.49 | indole-3-glycerol phosphate lyase, chloroplast precursor |
| 03g02190 | 10.91 | 30.53 | 1.48 | protein kinase domain containing protein, expressed |
| 03g13200 | 4.74 | 13.17 | 1.47 | peroxidase precursor, putative, expressed |
| **Table S3. (Continued)** | | | | |
| **Gene** | **TPM-SY** | **TPM-FR** | **log_2_(FR/SY)** | **Description** |
| 02g11720 | 5.15 | 14.17 | 1.46 | lipase, putative, expressed |
| 03g20090 | 5.97 | 16.36 | 1.45 | MYB family transcription factor, putative, expressed |
| 06g34440 | 20.38 | 55.47 | 1.44 | dnaJ domain containing protein, expressed |
| 08g14195 | 15.85 | 43.1 | 1.44 | expressed protein |
| 08g01110 | 5.97 | 16.16 | 1.44 | expressed protein |
| 04g53502 | 7.62 | 20.55 | 1.43 | expressed protein |
| 08g01150 | 4.53 | 12.17 | 1.43 | DTA2, putative, expressed |
| 05g45300 | 5.56 | 14.76 | 1.41 | enoyl-CoA hydratase/isomerase family protein, putative, expressed |
| 11g17540 | 5.35 | 13.97 | 1.38 | retrotransposon protein, putative, Ty1-copia subclass, expressed |
| 10g11354 | 7.41 | 19.15 | 1.37 | MATE efflux family protein, putative, expressed |
| 05g46610 | 6.38 | 16.36 | 1.36 | myb-like DNA-binding domain containing protein, putative, expressed |
| 06g11190 | 5.56 | 14.17 | 1.35 | OsPOP13 - Putative Prolyl Oligopeptidase homologue, expressed |
| 10g36950 | 7.82 | 19.35 | 1.31 | DUF677 domain containing protein, putative, expressed |
| 09g31410 | 5.76 | 14.17 | 1.30 | Os9bglu29 - beta-glucosidase homologue |
| 05g13580 | 21.41 | 52.47 | 1.29 | OsCML18 - Calmodulin-related calcium sensor protein, expressed |
| 12g06490 | 7.82 | 19.15 | 1.29 | STE_PAK_Ste20_Slob_Wnk.6 - STE kinases include homologs to sterile 7, sterile 11 and sterile 20 from yeast, expressed |
| 07g04210 | 5.97 | 14.57 | 1.29 | Ser/Thr protein phosphatase family protein, putative |
| 08g06100 | 229.97 | 558.27 | 1.28 | O-methyltransferase, putative, expressed |
| 07g01904 | 18.53 | 44.89 | 1.28 | expressed protein |
| 01g45830 | 7.21 | 17.36 | 1.27 | sulfate transporter, putative, expressed |
| 08g40560 | 12.15 | 29.13 | 1.26 | ZOS8-11 - C2H2 zinc finger protein, expressed |
| 01g53294 | 7 | 16.76 | 1.26 | respiratory burst oxidase protein B, putative, expressed |
| 06g15170 | 11.94 | 28.53 | 1.26 | 3-ketoacyl-CoA synthase, putative, expressed |
| 06g51460 | 8.24 | 19.55 | 1.25 | white-brown complex homolog protein, putative, expressed |
| 03g64050 | 9.06 | 21.35 | 1.24 | receptor protein kinase, putative, expressed |
| 11g45990 | 18.32 | 42.9 | 1.23 | von Willebrand factor type A domain containing protein |
| 01g35050 | 22.65 | 52.67 | 1.22 | early-responsive to dehydration protein-related |
| 02g49920 | 12.97 | 30.13 | 1.22 | 3-ketoacyl-CoA synthase, putative, expressed |
| 07g26940 | 32.74 | 75.82 | 1.21 | ORM1, putative, expressed |
| 01g01710 | 65.68 | 151.84 | 1.21 | 1-deoxy-D-xylulose 5-phosphate reductoisomerase |
| 05g12400 | 8.24 | 18.95 | 1.20 | BURP domain containing protein, expressed |
| 03g25960 | 25.32 | 57.66 | 1.19 | RNA recognition motif containing protein, putative, expressed |
| 03g22800 | 7.82 | 17.76 | 1.18 | OsFBT5 - F-box and tubby domain containing protein, expressed |
| 06g13190 | 76.38 | 172.59 | 1.18 | expressed protein |
| 03g51610 | 45.09 | 101.56 | 1.17 | Inositol 1, 3, 4-trisphosphate 5/6-kinase, putative, expressed |
| 08g01140 | 9.88 | 21.95 | 1.15 | cytochrome b561, putative, expressed |
| 08g36170 | 7.82 | 17.36 | 1.15 | cytokinesis negative regulator RCP1, putative, expressed |
| 04g54240 | 8.65 | 19.15 | 1.15 | wound induced protein, putative, expressed |
| 06g03486 | 11.12 | 24.54 | 1.14 | expressed protein |
| 07g31300 | 68.77 | 151.64 | 1.14 | ATP synthase delta chain, mitochondrial precursor, putative, expressed |
| **Table S3. (Continued)** | | | | |
| **Gene** | **TPM-SY** | **TPM-FR** | **log_2_(FR/SY)** | **Description** |
| 11g39670 | 26.77 | 58.66 | 1.13 | seryl-tRNA synthetase, putative, expressed |
| 09g36680 | 116.53 | 254.79 | 1.13 | ribonuclease T2 family domain containing protein, expressed |
| 03g27840 | 12.35 | 26.94 | 1.13 | splicing factor, arginine/serine-rich 16, putative, expressed |
| 10g05980 | 56 | 121.91 | 1.12 | POEI13 - Pollen Ole e I allergen and extensin family protein precursor |
| 08g34280 | 52.5 | 114.13 | 1.12 | cinnamoyl-CoA reductase, putative, expressed |
| 10g28350 | 11.74 | 25.34 | 1.11 | 1,2-dihydroxy-3-keto-5-methylthiopentene dioxygenase protein |
| 03g19270 | 8.03 | 17.16 | 1.10 | universal stress protein domain containing protein |
| 12g29660 | 8.03 | 17.16 | 1.10 | DEAD-box ATP-dependent RNA helicase, putative, expressed |
| 03g49720 | 15.03 | 31.92 | 1.09 | PAP fibrillin family domain containing protein, expressed |
| 09g26460 | 11.12 | 23.54 | 1.08 | protein binding protein, putative, expressed |
| 03g22200 | 8.44 | 17.76 | 1.07 | nodulin MtN3 family protein, putative, expressed |
| 11g02440 | 9.68 | 20.35 | 1.07 | chalcone--flavonone isomerase, putative, expressed |
| 01g32770 | 44.47 | 93.18 | 1.07 | DUF260 domain containing protein, putative, expressed |
| 07g41090 | 10.5 | 21.95 | 1.06 | histone deacetylase, putative, expressed |
| 07g11290 | 16.47 | 34.32 | 1.06 | expressed protein |
| 10g35020 | 9.88 | 20.55 | 1.06 | glycosyltransferase, putative, expressed |
| 06g07969 | 89.56 | 184.96 | 1.05 | cytochrome b-c1 complex subunit 8, putative, expressed |
| 04g55850 | 9.47 | 19.55 | 1.05 | nuclease PA3, putative, expressed |
| 01g16910 | 11.12 | 22.95 | 1.05 | outer mitochondrial membrane porin, putative, expressed |
| 02g50040 | 16.06 | 33.12 | 1.04 | endoglucanase, putative, expressed |
| 07g16040 | 11.32 | 23.34 | 1.04 | erythronate-4-phosphate dehydrogenase domain containing protein |
| 07g03120 | 12.76 | 26.14 | 1.03 | expressed protein |
| 10g17260 | 10.91 | 22.35 | 1.03 | cytochrome P450, putative, expressed |
| 03g13274 | 11.32 | 23.14 | 1.03 | peptide transporter PTR2, putative, expressed |
| 12g44020 | 15.85 | 32.32 | 1.03 | Ser/Thr protein phosphatase family protein, putative, expressed |
| 06g46284 | 20.18 | 41.1 | 1.03 | glycosyl hydrolase, family 31, putative, expressed |
| 08g43470 | 26.97 | 54.67 | 1.02 | ER lumen protein retaining receptor, putative, expressed |
| 02g27360 | 21 | 42.5 | 1.02 | aspartic proteinase-like protein 2 precursor, putative, expressed |
| 02g51140 | 14.41 | 29.13 | 1.02 | N-rich protein, putative, expressed |
| 10g14011 | 9.88 | 19.95 | 1.01 | expressed protein |
| 01g15780 | 10.09 | 20.35 | 1.01 | glycosyl transferase, group 1 domain containing protein, expressed |
| 05g47530 | 8.65 | 0.4 | -4.43 | Divergent PAP2 family domain containing protein, expressed |
| 02g22020 | 11.74 | 1 | -3.55 | MYB family transcription factor, putative, expressed |
| 01g73024 | 4.53 | 0.4 | -3.50 | expressed protein |
| 12g11990 | 4.53 | 0.4 | -3.50 | expressed protein |
| 08g33190 | 4.32 | 0.4 | -3.43 | linker histone H1 and H5 family protein, expressed |
| 07g44700 | 4.12 | 0.4 | -3.36 | expressed protein |
| 01g73000 | 9.88 | 1 | -3.30 | copine, putative, expressed |
| 08g08230 | 4.74 | 0.6 | -2.98 | kinesin-like protein, identical, putative, expressed |
| 08g35160 | 9.47 | 1.2 | -2.98 | heat shock protein DnaJ, putative, expressed |
| 06g06180 | 8.03 | 1.2 | -2.74 | transferase family protein, putative, expressed |
| **Table S3. (Continued)** | | | | |
| **Gene** | **TPM-SY** | **TPM-FR** | **log_2_(FR/SY)** | **Description** |
| 01g74330 | 5.97 | 1 | -2.58 | pectinacetylesterase domain containing protein, expressed |
| 01g23630 | 10.29 | 1.8 | -2.52 | transcription initiation factor IID, 18kD subunit family protein |
| 04g30240 | 13.59 | 2.39 | -2.51 | OsWAK60 - OsWAK receptor-like protein kinase, expressed |
| 08g41489 | 14 | 2.59 | -2.43 | expressed protein |
| 03g14140 | 5.97 | 1.2 | -2.31 | POEI16-Pollen Ole e I allergen and extensin family protein precursor |
| 09g06719 | 8.65 | 1.8 | -2.26 | expressed protein |
| 06g02340 | 7.62 | 1.6 | -2.25 | OsFBX183 - F-box domain containing protein, expressed |
| 12g33130 | 13.18 | 2.79 | -2.24 | expressed protein |
| 05g20954 | 6.38 | 1.4 | -2.19 | retrotransposon protein, putative, unclassified, expressed |
| 09g19650 | 9.06 | 2 | -2.18 | 3-ketoacyl-CoA synthase precursor, putative, expressed |
| 10g42960 | 8.03 | 1.8 | -2.16 | urea active transporter, putative, expressed |
| 05g19470 | 11.74 | 2.79 | -2.07 | expressed protein |
| 03g44290 | 12.56 | 2.99 | -2.07 | expansin precursor, putative, expressed |
| 10g10130 | 26.35 | 6.58 | -2.00 | OsWAK112d - OsWAK receptor-like protein kinase, expressed |
| 05g07680 | 7.82 | 2 | -1.97 | KIP1, putative, expressed |
| 08g05490 | 14.21 | 3.99 | -1.83 | tRNA synthetase, putative, expressed |
| 09g38670 | 9.68 | 2.79 | -1.79 | thioredoxin, putative, expressed |
| 11g37950 | 25.12 | 7.58 | -1.73 | WIP3 - Wound-induced protein precursor, expressed |
| 01g03680 | 27.38 | 8.38 | -1.71 | BBTI8 - Bowman-Birk type bran trypsin inhibitor precursor, expressed |
| 01g16880 | 9.06 | 2.79 | -1.70 | expressed protein |
| 02g47180 | 13.38 | 4.19 | -1.68 | WD repeat-containing protein, putative, expressed |
| 03g52860 | 28 | 8.78 | -1.67 | lipoxygenase, putative, expressed |
| 04g56430 | 40.56 | 12.97 | -1.64 | cysteine-rich receptor-like protein kinase, putative, expressed |
| 05g51830 | 88.12 | 28.33 | -1.64 | ZOS5-12 - C2H2 zinc finger protein, expressed |
| 01g72834 | 13.18 | 4.59 | -1.52 | RNA recognition motif containing protein, putative, expressed |
| 03g11540 | 19.35 | 6.78 | -1.51 | RPA1B -Putative single-stranded DNA binding complex subunit 1 |
| 01g71340 | 23.88 | 8.38 | -1.51 | glycosyl hydrolases family 17, putative, expressed |
| 03g46070 | 11.94 | 4.19 | -1.51 | thaumatin, putative, expressed |
| 06g06510 | 87.3 | 30.73 | -1.51 | histone H3, putative, expressed |
| 08g42700 | 13.59 | 4.79 | -1.50 | resistance protein, putative, expressed |
| 09g27830 | 41.18 | 14.57 | -1.50 | OsPDIL2-3 protein disulfide isomerase PDIL2-3, expressed |
| 11g02640 | 12.35 | 4.39 | -1.49 | expressed protein |
| 10g35070 | 14 | 4.99 | -1.49 | alpha-galactosidase precursor, putative, expressed |
| 01g11550 | 11.74 | 4.19 | -1.49 | TCP family transcription factor, putative, expressed |
| 04g44650 | 24.5 | 8.78 | -1.48 | ferredoxin-thioredoxin reductase, variable chain, putative, expressed |
| 02g57720 | 20.59 | 7.58 | -1.44 | aquaporin protein, putative, expressed |
| 02g15594 | 26.77 | 9.98 | -1.42 | protein phosphatase 2C, putative, expressed |
| 03g44900 | 18.74 | 7.18 | -1.38 | CCR4-NOT transcription factor, putative, expressed |
| 11g05080 | 15.44 | 5.99 | -1.37 | powdery mildew resistant protein 5, putative, expressed |
| 05g02300 | 30.68 | 11.97 | -1.36 | Core histone H2A/H2B/H3/H4 domain containing protein |
| 04g02680 | 14.82 | 5.79 | -1.36 | expressed protein |
| **Table S3. (Continued)** | | | | |
| **Gene** | **TPM-SY** | **TPM-FR** | **log_2_(FR/SY)** | **Description** |
| 04g50970 | 203.83 | 80.41 | -1.34 | seed specific protein Bn15D1B, putative, expressed |
| 05g31190 | 17.09 | 6.78 | -1.33 | expressed protein |
| 06g39120 | 21 | 8.38 | -1.33 | expressed protein |
| 03g47940 | 29.44 | 11.77 | -1.32 | GDSL-like lipase/acylhydrolase, putative, expressed |
| 01g72370 | 25.32 | 10.18 | -1.31 | helix-loop-helix DNA-binding domain containing protein |
| 12g20390 | 26.15 | 10.57 | -1.31 | expressed protein |
| 04g52640 | 13.79 | 5.59 | -1.30 | SHR5-receptor-like kinase, putative, expressed |
| 01g28450 | 29.03 | 11.77 | -1.30 | SCP-like extracellular protein, expressed |
| 03g02290 | 18.53 | 7.58 | -1.29 | kinesin motor domain containing protein, putative, expressed |
| 11g32280 | 18.32 | 7.58 | -1.27 | acetyltransferase, GNAT family, putative, expressed |
| 01g55950 | 18.74 | 7.78 | -1.27 | acetamidase, putative, expressed |
| 07g37140 | 14.41 | 5.99 | -1.27 | expressed protein |
| 10g41980 | 14.82 | 6.19 | -1.26 | RALFL26 - Rapid ALkalinization Factor RALF family protein precursor, expressed |
| 11g09160 | 48.18 | 20.35 | -1.24 | B3 DNA binding domain containing protein, expressed |
| 11g40970 | 15.03 | 6.38 | -1.24 | receptor-like protein kinase precursor, putative, expressed |
| 03g22950 | 18.74 | 7.98 | -1.23 | acyl carrier protein, putative, expressed |
| 12g43340 | 20.59 | 8.78 | -1.23 | actin-depolymerizing factor, putative, expressed |
| 02g32520 | 37.88 | 16.16 | -1.23 | ERD1 protein, chloroplast precursor, putative, expressed |
| 07g42310 | 17.71 | 7.58 | -1.22 | lung seven transmembrane domain containing protein, putative, expressed |
| 12g07160 | 14.41 | 6.19 | -1.22 | DUF869 domain containing protein |
| 08g32750 | 65.27 | 28.13 | -1.21 | bifunctional monodehydroascorbate reductase and carbonic anhydrasenectarin-3 precursor, putative, expressed |
| 02g17940 | 23.47 | 10.18 | -1.21 | leucoanthocyanidin dioxygenase, putative, expressed |
| 11g15040 | 16.47 | 7.18 | -1.20 | S-adenosyl-L-methionine:benzoic acid/salicylic acid carboxyl methyltransferase |
| 07g35560 | 27.18 | 11.97 | -1.18 | glucan endo-1,3-beta-glucosidase precursor, putative, expressed |
| 09g17740 | 122.09 | 54.67 | -1.16 | chlorophyll A-B binding protein, putative, expressed |
| 04g51454 | 16.47 | 7.38 | -1.16 | expressed protein |
| 09g32520 | 19.56 | 8.78 | -1.16 | 60S ribosomal protein L32, putative, expressed |
| 10g35190 | 18.12 | 8.18 | -1.15 | ZOS10-06 - C2H2 zinc finger protein, expressed |
| 02g06370 | 19.77 | 8.98 | -1.14 | whirly transcription factor domain containing protein, expressed |
| 06g10580 | 17.5 | 7.98 | -1.13 | cyclic nucleotide-gated ion channel, putative, expressed |
| 01g25920 | 179.74 | 82.8 | -1.12 | expressed protein |
| 06g19730 | 42 | 19.35 | -1.12 | HEAT repeat family protein, putative, expressed |
| 06g40120 | 39.94 | 18.56 | -1.11 | SPX domain containing protein, putative, expressed |
| 02g11840 | 123.74 | 57.66 | -1.10 | coatomer subunit beta-2, putative, expressed |
| 07g48830 | 17.09 | 7.98 | -1.10 | glycosyl transferase 8 domain containing protein, putative, expressed |
| 01g01410 | 17.5 | 8.18 | -1.10 | TKL_IRAK_C-LEC.1 - TKL_IRAK_C-LEC has homology to C-type lectin receptors, expressed |
| **Table S3. (Continued)** | | | | |
| **Gene** | **TPM-SY** | **TPM-FR** | **log_2_(FR/SY)** | **Description** |
| 03g58110 | 18.32 | 8.58 | -1.09 | uncharacterized protein At4g06744 precursor, putative, expressed |
| 01g41565 | 36.44 | 17.16 | -1.09 | ATP-binding domain-containing protein, putative |
| 02g29320 | 29.24 | 13.77 | -1.09 | expressed protein |
| 03g52390 | 38.5 | 18.16 | -1.08 | PIII1 - Proteinase inhibitor II family protein precursor, expressed |
| 11g41034 | 54.56 | 25.74 | -1.08 | expressed protein |
| 12g37260 | 58.27 | 27.53 | -1.08 | lipoxygenase 2.1, chloroplast precursor, putative, expressed |
| 08g30554 | 51.06 | 24.14 | -1.08 | expressed protein |
| 02g10920 | 31.09 | 14.76 | -1.07 | zinc finger family protein, putative, expressed |
| 02g20360 | 19.56 | 9.38 | -1.06 | tyrosine aminotransferase, putative, expressed |
| 05g41990 | 42.41 | 20.35 | -1.06 | peroxidase precursor, putative, expressed |
| 02g52340 | 22.44 | 10.77 | -1.06 | OsMADS22 - MADS-box family gene with MIKCc type-box |
| 02g18410 | 33.15 | 16.16 | -1.04 | salt stress root protein RS1, putative, expressed |
| 11g09820 | 21.62 | 10.57 | -1.03 | hypothetical protein |
| 01g25880 | 41.18 | 20.15 | -1.03 | dephospho-CoA kinase, putative, expressed |
| 01g63870 | 19.56 | 9.58 | -1.03 | nucleobase-ascorbate transporter, putative, expressed |
| 08g26230 | 182 | 89.19 | -1.03 | expressed protein |
| 08g10010 | 31.29 | 15.36 | -1.03 | acyl-desaturase, chloroplast precursor, putative, expressed |
| 05g47670 | 24.29 | 11.97 | -1.02 | zinc finger, C3HC4 type domain containing protein, expressed |
| 02g46473 | 22.24 | 10.97 | -1.02 | expressed protein |
| 01g48910 | 20.18 | 9.98 | -1.02 | AMP-binding enzyme, putative, expressed |
| 07g48570 | 24.5 | 12.17 | -1.01 | expressed protein |
| 06g49640 | 20.79 | 10.38 | -1.00 | uncharacterized UPF0114 domain containing protein, expressed |
