# Supplementary material for: Altered Levels of Histone Deacetylase OsHDT1 Affect Differential Gene Expression Patterns in Hybrid Rice
Source: PLoS One. 2011 Jul 8;6(7):e21789. doi: 10.1371/journal.pone.0021789 (PMC3132746; doi:10.1371/journal.pone.0021789)
Supplement: Table S4 — Genes showing FU>SY63>FR expression. (DOCX) [file pone.0021789.s008.docx]

**Table S4.** Genes showing the FU>SY63>FR expression profile

| **Gene**  **(LOC_Os)** | **TPM**  **-FR** | **TPM**  **-SY** | **TPM**  **-FU** | **log_2_**  **(FR/SY)** | **log_2_**  **(FU/SY)** | **Description** |
| --- | --- | --- | --- | --- | --- | --- |
| 01g71340 | 8.38 | 23.88 | 60.98 | -1.51 | 1.35 | glycosyl hydrolases family 17 |
| 03g52860 | 8.78 | 28 | 176.9 | -1.67 | 2.66 | lipoxygenase |
| 05g51830 | 28.33 | 88.12 | 1574 | -1.64 | 4.16 | ZOS5-12 - C2H2 zinc finger protein (*OsHDT1*) |
| 12g37260 | 27.53 | 58.27 | 474.7 | -1.08 | 3.03 | lipoxygenase 2.1, chloroplast precursor |
