# Supplementary material for: Altered Levels of Histone Deacetylase OsHDT1 Affect Differential Gene Expression Patterns in Hybrid Rice
Source: PLoS One. 2011 Jul 8;6(7):e21789. doi: 10.1371/journal.pone.0021789 (PMC3132746; doi:10.1371/journal.pone.0021789)
Supplement: Table S5 — Differentially expressed genes affected by OsHDT1 in SY63 background. (DOCX) [file pone.0021789.s009.docx]

**Table S5.** Genes showing above or below mid-parent expression affected by *OsHDT1* over-expression (FU) or RNAi (FR) in SY63 background

| **Gene**  **(LOC_Os)** | **TPM-FU** | **TPM-FR** | **TPM-SY** | **TPM-mid** | **log2(SY/mid)** | **log2(FU/SY)** | **log2(FR/SY)** | **Description** |
| --- | --- | --- | --- | --- | --- | --- | --- | --- |
| 09g27820 | 17.63 | 3.59 | 0.01 | 5.81 | -9.18 | 10.78 | 8.49 | 1-aminocyclopropane-1-carboxylate oxidase |
| 04g09920 | 3.32 |  | 0.01 | 5.11 | -9.00 | 8.38 |  | cytochrome P450 |
| 07g32710 | 51.85 | 22.55 | 0.01 | 5.01 | -8.97 | 12.34 | 11.14 | retrotransposon protein |
| 04g28234 | 6.22 |  | 0.01 | 4.21 | -8.72 | 9.28 |  | Rf1, mitochondrial precursor |
| 06g06470 | 4.77 |  | 0.01 | 3.51 | -8.46 | 8.90 |  | U-box domain containing heat shock protein |
| 07g05365 | 5.39 |  | 0.01 | 3.41 | -8.41 | 9.07 |  | photosystem II 10 kDa polypeptide |
| 10g42040 | 13.07 |  | 1.03 | 23.65 | -4.52 | 3.67 |  | expressed protein |
| 07g03730 | 99.14 |  | 8.65 | 155.3 | -4.17 | 3.52 |  | SCP-like extracellular protein |
| 11g31540 | 16.59 |  | 6.18 | 70.75 | -3.52 | 1.42 |  | BR1-associated receptor kinase 1 precursor |
| 07g24830 | 45.84 |  | 0.62 | 7.01 | -3.50 | 6.21 |  | thionin-like peptide |
| 01g16170 | 9.96 |  | 1.44 | 14.53 | -3.33 | 2.79 |  | PQ loop repeat domain containing protein |
| 06g36560 | 38.78 |  | 11.74 | 93.89 | -3.00 | 1.72 |  | inositol oxygenase |
| 11g31470 | 14.1 |  | 1.24 | 9.62 | -2.96 | 3.51 |  | expressed protein |
| 06g07030 | 14.52 | 11.77 | 3.5 | 22.05 | -2.66 | 2.05 | 1.75 | AP2 domain containing protein |
| 01g15270 | 531.8 |  | 156.1 | 979.2 | -2.65 | 1.77 |  | expressed protein |
| 07g01560 | 12.65 |  | 3.29 | 19.54 | -2.57 | 1.94 |  | transporter family protein |
| 02g29210 | 12.44 |  | 1.65 | 9.72 | -2.56 | 2.91 |  | ankyrin |
| 03g04060 | 18.46 |  | 7.41 | 42.59 | -2.52 | 1.32 |  | CHIT16-Chitinase family protein precursor |
| 11g09979 | 12.65 | 14.57 | 4.53 | 24.55 | -2.44 | 1.48 | 1.69 | expressed protein |
| 12g26940 | 6.43 |  | 1.44 | 7.62 | -2.40 | 2.16 |  | CHASE domain containing protein |
| 03g10320 | 10.37 |  | 1.65 | 8.52 | -2.37 | 2.65 |  | expressed protein |
| 01g38610 | 31.32 | 27.53 | 5.15 | 24.45 | -2.25 | 2.60 | 2.42 | helix-loop-helix domain containing protein |
| 05g05390 | 9.75 |  | 3.09 | 14.43 | -2.22 | 1.66 |  | expressed protein |
| 01g67770 | 6.43 |  | 1.44 | 6.61 | -2.20 | 2.16 |  | two-component response regulator |
| 03g45770 | 25.51 | 44.49 | 11.12 | 48.1 | -2.11 | 1.20 | 2.00 | expressed protein |
| 08g37370 | 53.92 |  | 22.65 | 97.4 | -2.10 | 1.25 |  | mitochondrial carrier protein |
| 06g07070 | 11.82 |  | 3.09 | 13.13 | -2.09 | 1.94 |  | BR1-associated receptor kinase 1 precursor |
| 03g20600 | 11.61 |  | 1.65 | 6.91 | -2.07 | 2.81 |  | expressed protein |
| 04g38530 | 26.75 |  | 11.74 | 47.3 | -2.01 | 1.19 |  | aldose 1-epimerase |
| 11g15040 |  | 7.18 | 16.47 | 66.04 | -2.00 |  | -1.20 | benzoate carboxyl methyltransferase |
| 09g17360 | 25.3 |  | 10.09 | 39.28 | -1.96 | 1.33 |  | expressed protein |
| 06g03486 | 41.06 | 24.54 | 11.12 | 42.19 | -1.92 | 1.88 | 1.14 | expressed protein |
| 02g54860 | 15.35 |  | 2.88 | 10.82 | -1.91 | 2.41 |  | ankyrin repeat-rich protein |
| 11g34910 | 11.2 | 8.78 | 2.47 | 9.12 | -1.88 | 2.18 | 1.83 | expressed protein |
| 05g12400 | 21.15 | 18.95 | 8.24 | 30.26 | -1.88 | 1.36 | 1.20 | BURP domain containing protein |
| 02g49920 | 35.47 | 30.13 | 12.97 | 47.4 | -1.87 | 1.45 | 1.22 | 3-ketoacyl-CoA synthase |
| 03g43510 | 14.93 |  | 3.71 | 12.73 | -1.78 | 2.01 |  | expressed protein |
| 01g11860 | 18.04 | 20.95 | 7.41 | 24.85 | -1.75 | 1.28 | 1.50 | DJ-1 family protein |
| 05g51830 | 1574 | 28.33 | 88.12 | 294.3 | -1.74 | 4.16 | -1.64 | ZOS5-12 - C2H2 zinc finger protein |
| 12g35270 | 12.44 |  | 4.74 | 15.73 | -1.73 | 1.39 |  | expressed protein |
| **Table S5. (Continued)** | | | | | | | | |
| **Gene**  **(LOC_Os)** | **TPM-FU** | **TPM-FR** | **TPM-SY** | **TPM-mid** | **log2(SY/mid)** | **log2(FU/SY)** | **log2(FR/SY)** | **Description** |
| 12g43640 | 12.65 |  | 4.74 | 15.63 | -1.72 | 1.42 |  | receptor-like protein kinase 5 precursor |
| 03g58980 | 12.44 |  | 3.91 | 12.83 | -1.71 | 1.67 |  | Cupin domain containing protein |
| 01g37280 | 17.01 | 20.35 | 7 | 22.85 | -1.71 | 1.28 | 1.54 | expressed protein |
| 05g05600 | 20.53 |  | 9.06 | 29.56 | -1.71 | 1.18 |  | ATA15 protein |
| 08g26230 |  | 89.19 | 182 | 593.2 | -1.70 |  | -1.03 | expressed protein |
| 03g29190 | 22.61 |  | 11.12 | 34.97 | -1.65 | 1.02 |  | PDI |
| 12g31880 | 11.2 |  | 3.09 | 9.62 | -1.64 | 1.86 |  | translation initiation factor |
| 08g10310 | 27.38 |  | 11.32 | 34.87 | -1.62 | 1.27 |  | SHR5-receptor-like kinase |
| 11g17540 | 18.67 | 13.97 | 5.35 | 16.43 | -1.62 | 1.80 | 1.38 | retrotransposon protein |
| 01g71340 | 60.98 | 8.38 | 23.88 | 72.35 | -1.60 | 1.35 | -1.51 | glycosyl hydrolases family 17 |
| 03g18130 | 66.78 |  | 12.35 | 37.18 | -1.59 | 2.43 |  | asparagine synthetase |
| 07g38830 | 12.44 |  | 4.74 | 13.93 | -1.56 | 1.39 |  | hydrolase, alpha/beta fold family protein |
| 01g67190 | 10.78 |  | 3.71 | 10.82 | -1.54 | 1.54 |  | ribonuclease T2 family protein |
| 12g38180 | 35.05 |  | 10.71 | 30.06 | -1.49 | 1.71 |  | heat shock cognate 70 kDa protein 2 |
| 02g48150 | 28 |  | 12.97 | 35.87 | -1.47 | 1.11 |  | expressed protein |
| 08g43040 | 16.18 |  | 3.91 | 10.72 | -1.46 | 2.05 |  | transferase family protein |
| 01g42410 | 29.24 |  | 13.38 | 36.68 | -1.45 | 1.13 |  | pleiotropic drug resistance protein |
| 03g13200 | 12.24 | 13.17 | 4.74 | 12.93 | -1.45 | 1.37 | 1.47 | peroxidase precursor |
| 01g03330 | 30.9 |  | 6.38 | 17.24 | -1.43 | 2.28 |  | BBTI3-Bowman-Birk type bran trypsin inhibitor precursor |
| 01g35050 | 47.7 | 52.67 | 22.65 | 60.62 | -1.42 | 1.07 | 1.22 | early-responsive to dehydration protein |
| 11g42220 | 19.5 |  | 8.24 | 22.05 | -1.42 | 1.24 |  | laccase precursor protein |
| 01g24710 | 134.4 |  | 28.21 | 75.25 | -1.42 | 2.25 |  | jacalin-like lectin domain containing protein |
| 08g42700 | 5.18 | 4.79 | 13.59 | 35.77 | -1.40 | -1.39 | -1.50 | resistance protein |
| 06g08140 | 15.14 |  | 5.15 | 13.43 | -1.38 | 1.56 |  | protein phosphatase 2C |
| 03g13274 |  | 23.14 | 11.32 | 28.96 | -1.36 |  | 1.03 | peptide transporter PTR2 |
| 01g17470 | 13.9 |  | 5.56 | 14.13 | -1.35 | 1.32 |  | plastocyanin-like domain containing protein |
| 07g07410 | 15.35 |  | 6.59 | 16.63 | -1.34 | 1.22 |  | oxidoreductase, 2OG-Fe oxygenase protein |
| 08g36170 |  | 17.36 | 7.82 | 19.64 | -1.33 |  | 1.15 | cytokinesis negative regulator RCP1 |
| 01g13420 | 17.84 |  | 5.76 | 14.43 | -1.32 | 1.63 |  | SOUL heme-binding protein |
| 03g20670 | 56.2 |  | 22.85 | 56.92 | -1.32 | 1.30 |  | ELMO/CED-12 family protein |
| 01g01710 |  | 151.8 | 65.68 | 161.6 | -1.30 |  | 1.21 | 1-deoxy-D-xylulose 5-phosphate reductoisomerase, chloroplast precursor |
| 11g10520 | 61.39 |  | 28.41 | 69.44 | -1.29 | 1.11 |  | dehydrogenase |
| 05g48870 | 25.1 |  | 11.32 | 27.66 | -1.29 | 1.15 |  | auxin response factor 15 |
| 05g45210 | 21.57 | 19.55 | 6.79 | 16.33 | -1.27 | 1.67 | 1.53 | respiratory burst oxidase |
| 01g32770 |  | 93.18 | 44.47 | 106.8 | -1.26 |  | 1.07 | DUF260 domain containing protein |
| 07g01990 | 37.12 |  | 14.21 | 33.77 | -1.25 | 1.39 |  | expressed protein |
| 09g31130 | 1.45 |  | 11.32 | 26.86 | -1.25 | -2.96 |  | citrate transporter |
| 09g38790 | 17.84 |  | 6.59 | 15.63 | -1.25 | 1.44 |  | ZOS9-19 - C2H2 zinc finger protein |
| 03g10140 | 28.21 |  | 11.12 | 26.35 | -1.24 | 1.34 |  | ZOS3-04 - C2H2 zinc finger protein |
| **Table S5. (Continued)** | | | | | | | | |
| **Gene**  **(LOC_Os)** | **TPM-FU** | **TPM-FR** | **TPM-SY** | **TPM-mid** | **log2(SY/mid)** | **log2(FU/SY)** | **log2(FR/SY)** | **Description** |
| 02g14480 | 14.52 |  | 5.76 | 13.63 | -1.24 | 1.33 |  | receptor-like kinase |
| 01g03320 | 466.9 |  | 53.53 | 126.3 | -1.24 | 3.12 |  | BBTI2-Bowman-Birk type bran trypsin inhibitor precursor |
| 08g32750 |  | 28.13 | 65.27 | 153.0 | -1.23 |  | -1.21 | carbonic anhydrase precursor |
| 05g46610 |  | 16.36 | 6.38 | 14.83 | -1.22 |  | 1.36 | myb-like DNA-binding domain protein |
| 01g71670 | 21.98 |  | 7.82 | 18.04 | -1.21 | 1.49 |  | glycosyl hydrolases family 17 |
| 10g40934 | 42.1 |  | 18.74 | 42.79 | -1.19 | 1.17 |  | flavonol synthase/flavanone 3-hydroxylase |
| 01g17050 | 14.1 |  | 5.97 | 13.63 | -1.19 | 1.24 |  | VQ domain containing protein |
| 10g39430 | 21.36 |  | 9.26 | 21.14 | -1.19 | 1.21 |  | expressed protein |
| 03g22800 |  | 17.76 | 7.82 | 17.84 | -1.19 |  | 1.18 | F-box and tubby domain containing protein |
| 12g31450 | 19.7 |  | 8.24 | 18.54 | -1.17 | 1.26 |  | plastid division regulator MinE |
| 10g42020 | 64.71 |  | 17.5 | 39.18 | -1.16 | 1.89 |  | RALFL29 - Rapid ALkalinization Factor |
| 01g70240 | 25.92 |  | 7.62 | 17.04 | -1.16 | 1.77 |  | expressed protein |
| 12g36670 | 21.15 |  | 9.47 | 21.04 | -1.15 | 1.16 |  | F-box/LRR-repeat protein 3 |
| 10g11354 |  | 19.15 | 7.41 | 16.43 | -1.15 |  | 1.37 | MATE efflux family protein |
| 06g01934 | 25.51 |  | 12.35 | 27.36 | -1.15 | 1.05 |  | homeobox domain containing protein |
| 03g03034 | 20.12 |  | 8.44 | 18.64 | -1.14 | 1.25 |  | flavonol synthase/flavanone 3-hydroxylase |
| 06g28820 | 22.61 |  | 9.06 | 19.94 | -1.14 | 1.32 |  | cycloartenol synthase |
| 04g43820 | 17.01 |  | 7.62 | 16.53 | -1.12 | 1.16 |  | expressed protein |
| 07g13234 | 20.33 |  | 9.68 | 20.94 | -1.11 | 1.07 |  | expressed protein |
| 09g31486 | 17.01 |  | 7.62 | 16.33 | -1.10 | 1.16 |  | DnaK family protein |
| 08g01140 |  | 21.95 | 9.88 | 21.14 | -1.10 |  | 1.15 | cytochrome b561 |
| 03g03590 | 22.81 |  | 11.12 | 23.45 | -1.08 | 1.04 |  | transporter, proton antiporter-2 family |
| 04g43800 | 21.78 |  | 7.82 | 16.43 | -1.07 | 1.48 |  | phenylalanine ammonia-lyase |
| 09g26400 | 41.06 |  | 17.91 | 37.58 | -1.07 | 1.20 |  | zinc finger, C3HC4 type domain protein |
| 07g12340 | 23.85 |  | 9.47 | 19.84 | -1.07 | 1.33 |  | NAC domain-containing protein 67 |
| 01g11650 | 35.26 |  | 12.76 | 26.55 | -1.06 | 1.47 |  | GDSL-like lipase/acylhydrolase |
| 06g03860 | 23.64 |  | 11.74 | 23.55 | -1.00 | 1.01 |  | uncharacterized membrane protein |
| 02g05470 | 33.18 |  | 12.56 | 25.15 | -1.00 | 1.40 |  | CCT motif family protein |
| 09g38450 | 0.41 |  | 5.76 | 0.01 | 9.17 | -3.81 |  | expressed protein |
| 04g01690 | 13.48 |  | 5.56 | 0.01 | 9.12 | 1.28 |  | pyridoxal-dependent decarboxylase protein |
| 12g27220 | 25.92 |  | 3.71 | 0.01 | 8.54 | 2.80 |  | transferase family protein |
| 03g07250 | 0.01 |  | 3.09 | 0.01 | 8.27 | -8.27 |  | cytochrome P450 |
| 03g52860 | 176.9 | 8.78 | 28 | 0.5 | 5.81 | 2.66 | -1.67 | lipoxygenase |
| 11g10590 | 24.89 |  | 8.65 | 0.2 | 5.43 | 1.52 |  | hypothetical protein |
| 12g14440 | 1573 |  | 424.7 | 12.43 | 5.09 | 1.89 |  | Jacalin-like lectin domain protein |
| 01g03680 |  | 8.38 | 27.38 | 1 | 4.78 |  | -1.71 | BBTI8-Bowman-Birk type bran trypsin inhibitor precursor |
| 06g06510 | 36.29 | 30.73 | 87.3 | 4.11 | 4.41 | -1.27 | -1.51 | histone H3 |
| 04g15840 | 0.41 | 0.01 | 4.12 | 0.2 | 4.36 | -3.33 | -8.69 | expansin precursor |
|  |  |  |  |  |  |  |  |  |
| **Table S5. (Continued)** | | | | | | | | |
| **Gene**  **(LOC_Os)** | **TPM-FU** | **TPM-FR** | **TPM-SY** | **TPM-mid** | **log2(SY/mid)** | **log2(FU/SY)** | **log2(FR/SY)** | **Description** |
| 11g45990 | 67.4 | 42.9 | 18.32 | 0.9 | 4.35 | 1.88 | 1.23 | von Willebrand factor type A domain containing protein |
| 03g07100 | 7.88 |  | 20.38 | 1.2 | 4.09 | -1.37 |  | LTPL82-Protease inhibitor/seed storage/LTP family protein precursor |
| 01g63500 | 0.41 |  | 4.94 | 0.3 | 4.04 | -3.59 |  | expressed protein |
| 08g01100 | 11.61 |  | 26.77 | 1.7 | 3.98 | -1.21 |  | HMG1/2 |
| 03g02290 | 7.67 | 7.58 | 18.53 | 1.3 | 3.83 | -1.27 | -1.29 | kinesin motor domain containing protein |
| 10g01570 | 3.94 |  | 12.76 | 0.9 | 3.83 | -1.70 |  | C-5 cytosine-specific DNA methylase |
| 04g59260 | 28.83 |  | 8.44 | 0.6 | 3.81 | 1.77 |  | peroxidase precursor |
| 05g02300 | 12.65 | 11.97 | 30.68 | 2.51 | 3.61 | -1.28 | -1.36 | Histone H2A |
| 03g11540 | 6.22 | 6.78 | 19.35 | 1.6 | 3.6 | -1.64 | -1.51 | RPA1B-Single-stranded DNA binding complex subunit 1 |
| 09g37650 | 2.49 |  | 8.44 | 0.7 | 3.59 | -1.76 |  | flavin-containing monooxygenase protein |
| 02g47150 | 7.05 |  | 15.65 | 1.3 | 3.59 | -1.15 |  | DNA topoisomerase 2 |
| 12g09700 | 48.12 |  | 8.03 | 0.7 | 3.52 | 2.58 |  | Jacalin-like lectin domain protein |
| 08g07080 | 20.74 |  | 4.53 | 0.4 | 3.5 | 2.19 |  | terpene synthase |
| 12g36220 | 369 |  | 127.0 | 11.72 | 3.44 | 1.54 |  | inhibitor I family protein |
| 12g27350 | 121.1 |  | 19.56 | 1.9 | 3.36 | 2.63 |  | 10-deacetylbaccatin III 10-O-acetyltransferase |
| 07g23520 | 22.4 |  | 88.32 | 8.62 | 3.36 | -1.98 |  | expressed protein |
| 03g45760 | 2.49 |  | 9.68 | 1 | 3.28 | -1.96 |  | expressed protein |
| 12g02470 | 40.86 |  | 15.85 | 1.8 | 3.14 | 1.37 |  | OsWRKY65 |
| 05g06140 | 30.49 |  | 12.97 | 1.5 | 3.11 | 1.23 |  | Lipase |
| 05g31040 |  | 0.01 | 3.91 | 0.5 | 2.97 |  | -8.61 | cytokinin dehydrogenase precursor |
| 11g46000 | 71.55 |  | 30.68 | 4.01 | 2.94 | 1.22 |  | von Willebrand factor type A domain containing protein |
| 10g40810 | 4.15 |  | 11.53 | 1.7 | 2.76 | -1.47 |  | GATA zinc finger domain containing protein |
| 04g27670 | 29.24 |  | 7.82 | 1.2 | 2.7 | 1.90 |  | terpene synthase family protein |
| 10g37850 | 45.42 |  | 19.56 | 3.01 | 2.7 | 1.22 |  | Armadillo |
| 07g08500 | 7.67 |  | 17.09 | 2.71 | 2.66 | -1.16 |  | C-5 cytosine-specific DNA methylase |
| 03g44900 |  | 7.18 | 18.74 | 3.01 | 2.64 |  | -1.38 | CCR4-NOT transcription factor |
| 12g36210 | 289.3 |  | 114.9 | 18.54 | 2.63 | 1.33 |  | inhibitor I family protein |
| 03g62670 | 12.03 |  | 30.06 | 5.61 | 2.42 | -1.32 |  | expressed protein |
| 04g21350 | 18.25 |  | 48.59 | 9.32 | 2.38 | -1.41 |  | flowering promoting factor-like 1 |
| 02g52040 | 5.39 |  | 13.59 | 3.01 | 2.17 | -1.33 |  | phosphate-induced protein 1 |
| 04g02680 |  | 5.79 | 14.82 | 3.31 | 2.16 |  | -1.36 | expressed protein |
| 03g47940 | 8.09 | 11.77 | 29.44 | 6.61 | 2.16 | -1.86 | -1.32 | GDSL-like lipase/acylhydrolase |
| 05g30500 | 153.3 |  | 57.85 | 13.03 | 2.15 | 1.41 |  | expressed protein |
| 12g39830 | 7.47 |  | 19.35 | 4.61 | 2.07 | -1.37 |  | cyclin delta-3 |
| 02g22020 | 0.41 | 1 | 11.74 | 2.81 | 2.06 | -4.84 | -3.55 | MYB family transcription factor |
| **Table S5. (Continued)** | | | | | | | | |
| **Gene**  **(LOC_Os)** | **TPM-FU** | **TPM-FR** | **TPM-SY** | **TPM-mid** | **log2(SY/mid)** | **log2(FU/SY)** | **log2(FR/SY)** | **Description** |
| 01g11550 |  | 4.19 | 11.74 | 2.91 | 2.01 |  | -1.49 | TCP family transcription factor |
| 04g08630 | 3.53 |  | 28.41 | 7.42 | 1.94 | -3.01 |  | expressed protein |
| 11g37950 |  | 7.58 | 25.12 | 7.11 | 1.82 |  | -1.73 | WIP3 - Wound-induced protein precursor |
| 03g13130 | 10.37 |  | 21.62 | 6.31 | 1.78 | -1.06 |  | ternary complex factor MIP1 |
| 02g13660 | 13.27 |  | 45.91 | 13.43 | 1.77 | -1.79 |  | meiosis 5 |
| 03g44290 | 2.9 | 2.99 | 12.56 | 3.71 | 1.76 | -2.11 | -2.07 | expansin precursor |
| 01g72370 |  | 10.18 | 25.32 | 8.12 | 1.64 |  | -1.31 | helix-loop-helix domain containing protein |
| 10g39740 | 18.87 |  | 52.5 | 17.14 | 1.61 | -1.48 |  | glutathione S-transferase |
| 02g11840 |  | 57.66 | 123.7 | 40.68 | 1.6 |  | -1.10 | coatomer subunit beta-2 |
| 01g11710 | 13.48 |  | 37.47 | 12.33 | 1.6 | -1.47 |  | GDSL-like lipase/acylhydrolase |
| 02g15594 | 8.71 | 9.98 | 26.77 | 8.82 | 1.6 | -1.62 | -1.42 | protein phosphatase 2C |
| 08g10010 | 8.5 | 15.36 | 31.29 | 10.62 | 1.56 | -1.88 | -1.03 | acyl-desaturase, chloroplast precursor |
| 04g34170 | 191.0 |  | 42.62 | 14.93 | 1.51 | 2.16 |  | retrotransposon protein |
| 07g31840 | 14.52 |  | 14 | 4.91 | 1.51 | 2.05 |  | leucine-rich repeat family protein |
| 10g35070 |  | 4.99 | 14 | 4.91 | 1.51 |  | -1.49 | alpha-galactosidase precursor |
| 07g02200 | 10.37 |  | 21.41 | 7.52 | 1.51 | -1.05 |  | plastocyanin-like domain containing protein |
| 09g27830 |  | 14.57 | 41.18 | 14.53 | 1.5 |  | -1.50 | OsPDIL2-3 protein disulfide isomerase |
| 07g31650 | 3.73 |  | 11.32 | 4.01 | 1.5 | -1.60 |  | expressed protein |
| 12g06660 | 3.11 |  | 10.91 | 3.91 | 1.48 | -1.81 |  | Actin |
| 06g12455 | 34.43 |  | 11.74 | 4.21 | 1.48 | 1.55 |  | expressed protein |
| 05g11810 | 38.37 |  | 78.24 | 28.26 | 1.47 | -1.03 |  | gibberellin 2-beta-dioxygenase 1 |
| 03g06510 | 11.82 |  | 25.53 | 9.62 | 1.41 | -1.11 |  | KIP1 |
| 07g03120 |  | 26.14 | 12.76 | 4.81 | 1.41 |  | 1.03 | expressed protein |
| 03g41060 | 8.71 |  | 17.91 | 6.81 | 1.4 | -1.04 |  | Gibberellin-regulated protein precursor |
| 08g10510 | 17.63 |  | 37.06 | 14.13 | 1.39 | -1.07 |  | Aminotransferase |
| 12g20390 | 10.37 | 10.57 | 26.15 | 10.02 | 1.38 | -1.33 | -1.31 | expressed protein |
| 03g49430 | 6.43 |  | 17.09 | 6.61 | 1.37 | -1.41 |  | pre-mRNA-splicing factor |
| 02g08220 | 5.39 |  | 15.24 | 6.01 | 1.34 | -1.50 |  | expressed protein |
| 02g47180 |  | 4.19 | 13.38 | 5.31 | 1.33 |  | -1.68 | WD repeat-containing protein |
| 07g46920 | 77.57 |  | 25.32 | 10.12 | 1.32 | 1.62 |  | sex determination protein tasselseed-2 |
| 04g01710 | 25.51 |  | 12.15 | 5.01 | 1.28 | 1.07 |  | cysteine proteinase At4g11310 precursor |
| 12g07160 |  | 6.19 | 14.41 | 6.01 | 1.26 |  | -1.22 | DUF869 domain containing protein |
| 10g10130 | 5.18 | 6.58 | 26.35 | 11.12 | 1.24 | -2.35 | -2.00 | OsWAK receptor-like protein kinase |
| 09g37240 | 7.88 |  | 18.74 | 7.92 | 1.24 | -1.25 |  | glutathione S-transferase |
| 06g40150 | 19.91 |  | 39.94 | 16.93 | 1.24 | -1.00 |  | AP2 domain containing protein |
| 04g53950 | 11.2 |  | 23.68 | 10.32 | 1.2 | -1.08 |  | glycosyl hydrolases family 16 protein |
| 04g44650 | 12.03 | 8.78 | 24.5 | 10.72 | 1.19 | -1.03 | -1.48 | ferredoxin-thioredoxin reductase |
| 11g05080 |  | 5.99 | 15.44 | 6.81 | 1.18 |  | -1.37 | powdery mildew resistant protein 5 |
| 04g51454 | 5.18 | 7.38 | 16.47 | 7.42 | 1.15 | -1.67 | -1.16 | expressed protein |
| 08g25820 | 7.05 |  | 16.68 | 7.52 | 1.15 | -1.24 |  | myb-like DNA-binding domain protein |
| 05g02530 | 57.24 |  | 17.29 | 7.82 | 1.14 | 1.73 |  | glutathione S-transferase |
| **Table S5. (Continued)** | | | | | | | | |
| **Gene**  **(LOC_Os)** | **TPM-FU** | **TPM-FR** | **TPM-SY** | **TPM-mid** | **log2(SY/mid)** | **log2(FU/SY)** | **log2(FR/SY)** | **Description** |
| 12g43340 |  | 8.78 | 20.59 | 9.52 | 1.11 |  | -1.23 | actin-depolymerizing factor |
| 01g41565 | 16.38 | 17.16 | 36.44 | 17.24 | 1.08 | -1.15 | -1.09 | ATP-binding domain-containing protein |
| 07g07930 | 7.26 |  | 17.91 | 8.52 | 1.07 | -1.30 |  | LTPL78 - Protease inhibitor/seed storage/LTP family protein precursor |
| 05g13940 | 1426. |  | 685 | 327.2 | 1.07 | 1.06 |  | retrotransposon protein |
| 01g48910 |  | 9.98 | 20.18 | 9.82 | 1.04 |  | -1.02 | AMP-binding enzyme |
| 03g22950 |  | 7.98 | 18.74 | 9.12 | 1.04 |  | -1.23 | acyl carrier protein |
| 02g46910 | 17.21 |  | 39.74 | 19.34 | 1.04 | -1.21 |  | glycosyl hydrolases family 16 |
| 10g36650 | 22.4 |  | 45.71 | 22.45 | 1.03 | -1.03 |  | actin |
| 12g37260 | 474.7 | 27.53 | 58.27 | 28.96 | 1.01 | 3.03 | -1.08 | lipoxygenase 2.1, chloroplast precursor |

Blank cells mean FDR>0.001 or |log_2_[FU(FR)/SY]|<1.
