# Supplementary material for: Altered Levels of Histone Deacetylase OsHDT1 Affect Differential Gene Expression Patterns in Hybrid Rice
Source: PLoS One. 2011 Jul 8;6(7):e21789. doi: 10.1371/journal.pone.0021789 (PMC3132746; doi:10.1371/journal.pone.0021789)
Supplement: Table S6 — Primers used in this study. (DOCX) [file pone.0021789.s010.docx]

**Table S6.** Primers used in this study

| **Name** | **Forward primer sequence (5’-3’)** | | **Reverse primer sequence (5’-3’)** | |
| --- | --- | --- | --- | --- |
| Vector construction | | |  | |
| HDT1 | GGTACCCCGATTCCGATGGAGTTCTG | | GGATCCTCACTTGGCGGGGTGCTTGG | |
| RNAi | ACTAGTGGTACCGGCTGCAGTGAATGACGATG | | GAGCTCGGATCCTCACTTGGCGGGGTGCTTGG | |
| Expression analysis | | |  | |
| HDT | CGCTTTTTGCACCTTTCTCAG | | ACTTTGCCATTTGCCCTGG | |
| Hd3a | ­GCTCACTATCATCATCCAGCATG | | CCTTGCTCAGCTATTTAATTGCATAA | |
| RFT1 | TGGTGTTCGTGCTGTTCCA | | TTGTAGAGCTCGGCGAAGTTC | |
| Hd1 | TCAG CAACAGCATATCT TTCTCATCA | | TCTGGAATTTGGCTATACTATCACC | |
| Ehd1 | GGATGCAAGGAAATCATGGA | | AATCCCATCGGAAATCTTGG | |
| OsGI | TGGAGAAAGGTTGTGGATGC | | GATAGACGGCACTTCAGCAGAT | |
| Ghd7 | AAATCCGGTACGCGTCCAG | | GACATAGGTGGATGGCGGTG | |
| CCA | CCCATTCCAATTCGGTGAATC | | CGGACGTAAATCGCTCCAAGT | |
| LHY | TGCCCAATGGATAATGCACAG | | ACGGTGTGTTGGAACCACATG | |
| TOC | ACACCACAAACGTTGCTCCCT | | CGTTCCTTCCTTTTCAGCCTG | |
| 01g03320 | TCCAAGGCTCCAAGCAAAGTA | CGCCTGTTTGTTTCCATCG | |  |
| 01g03330 | TGCACACCATGCACTCAGAA | | TCTGGATGATCGAACGAACG | |
| 01g03360 | GCAGCTTTTGTGCATCTTGC | | CCACGCTTGCATTGCATATT | |
| 01g03680 | TGTCACAACCAGCCAGCTTAA | | TTAACGCTGATCGAGCGGT | |
| 01g04050 | TGTTCAAGTGCACCGACACCT | | AGCAATGGCTCGCGTTCAT | |
| 01g24710 | TTGGCATCTACGTCCACCCTT | | TCGGTGCAACACGTACACAGA | |
| 03g18130 | GTTTTCTCACCAATCGCCCAT | | TCTCGCACACGTACAACGACA | |
| 03g52860 | ACACGCTGCTCTACCCAAACA | | AACTACTCTTGTCCACGCTGGC | |
| 04g23940 | GCAAGAAGACAACGACGGAGA | | TACGTTGAGCGAGGTGGACAT | |
| 04g34170 | TCACGTTCCGACCAAAGGA | | AATTAAAGGCAGCGCAGCTC | |
| 06g24990 | TGCACCCCAAGAACGTCTACT | | CCATGCTGGTGTAGTTGCTCA | |
| 06g37150 | CAGCGAGGAAGAGCTTTGAGA | | TTCCCCTGCTCGACATTGA | |
| 07g03730 | GCGTCTTCATCACCTGCAACT | | CCGGATCAGCGTACGATAGTAG | |
| 07g48200 | CCATTTCAGCATGCACAAAGG | | TTGCAACACGAAGATGCGG | |
| 08g40170 | AAGTTTGGCCAGGAGTGAGCA | | TCAAGAGCATCAGCGTCGAGA | |
| 11g29380 | GCCAAGTTGAACGTGTTTCCC | | GGATTCGATATGCCTTACGGC | |
| 12g39830 | TGGAGTCCAAGATGAGCAACC | | TTGCAATGGTCGCTTGCTG | |
| ACTIN | TGCTATGTACGTC GCCATCCAG | | AATGAGTAACCACGCTCCGTCA | |
| CHIP assay analysis | | |  | |
| Hd1-1  Hd1-2 | TGAGAAGAGAAACGCTGAAGCA  ACAAGAGCCATGCGAGGTAGA | | TGTCAAGTCGAATCCAGCGAT  GCTTGCACCACATGGACAAGT | |
| Ehd1 | AATCTCGAAAAACCCGGTCA | | CATTTTCTAGAGGCGCTAGCG | |
| OsGI-1  OsGI-2 | TGAAAAATTCGGAGAAAGAACTCAG  CATCGGATAGATCAACGCGAG | | CCTATTCCATGCGTCCCAAA  TAAACGCCTCCACGAAGCA | |
| ACTIN | TGCGTGTCCTCTTCCTCATCT | | AAGGCGAATGAAGCGAAAAG | |
